# Supplementary material for: Microbial Precipitation of Pb(II) with Wild Strains of Paraclostridium bifermentans and Klebsiella pneumoniae Isolated from an Industrially Obtained Microbial Consortium
Source: Int J Mol Sci. 2022 Oct 14;23(20):12255. doi: 10.3390/ijms232012255 (PMC9603858; doi:10.3390/ijms232012255)
Supplement: Supplementary file 1 [file ijms-23-12255-s001.zip › ijms-1944492-supplementary.pdf]

Supplementary Materials: Microbial Precipitation of Pb(II) with Wild Strains *Paraclostridium bifermentans* and *Klebsiella pneumoniae* Isolated From an Industrially Obtained Microbial Consortium

Olga Neveling [1], Thato Ncube [1], Ziyanda Ngxongo [1], Evans M. Chirwa [1] and Hendrik G. Brink [1]*

1. Supplementary Figures

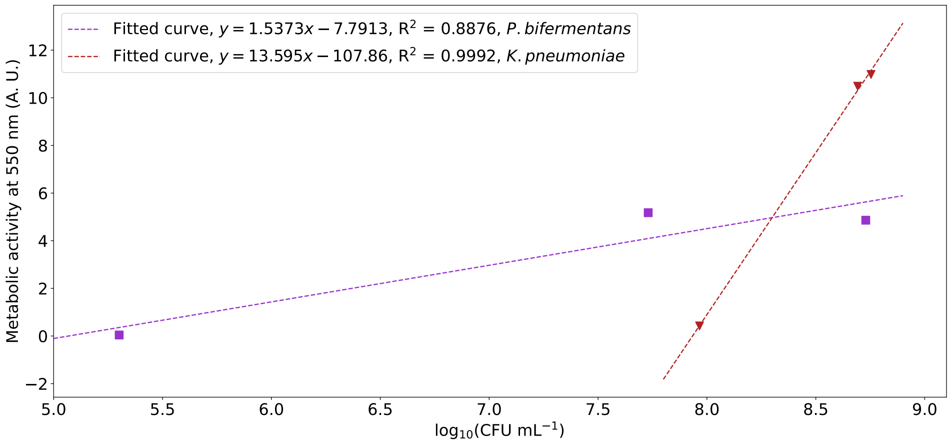

Figure S1. The relationship between metabolic activity and CFU count for *K. pneumoniae* and *P. bifermentans* with 80 mg L^{-1} initial concentration Pb(II).

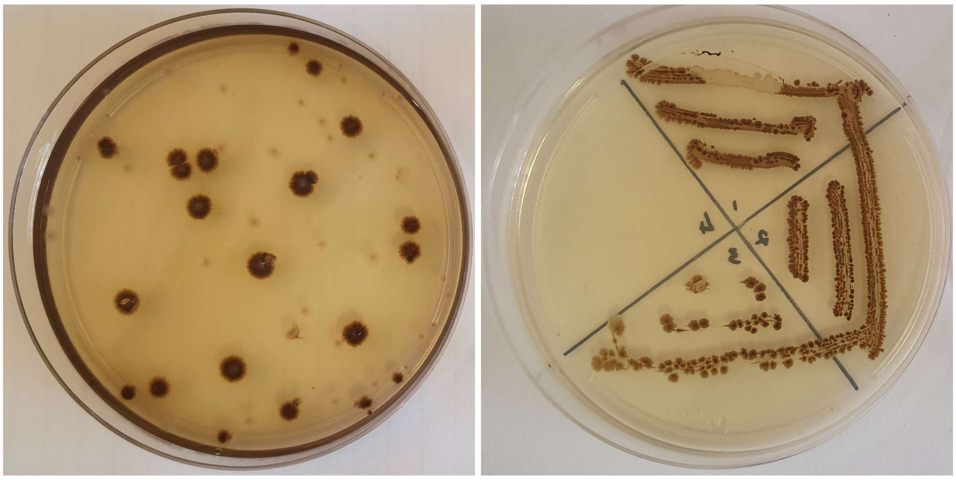

(a) **(b)**

Figure S2. The agar plates used in the isolation of *P. bifermentans* with (a) the initial spread plate of the consortium and (b) the final streak plate containing the single strain *P. bifermentans*.

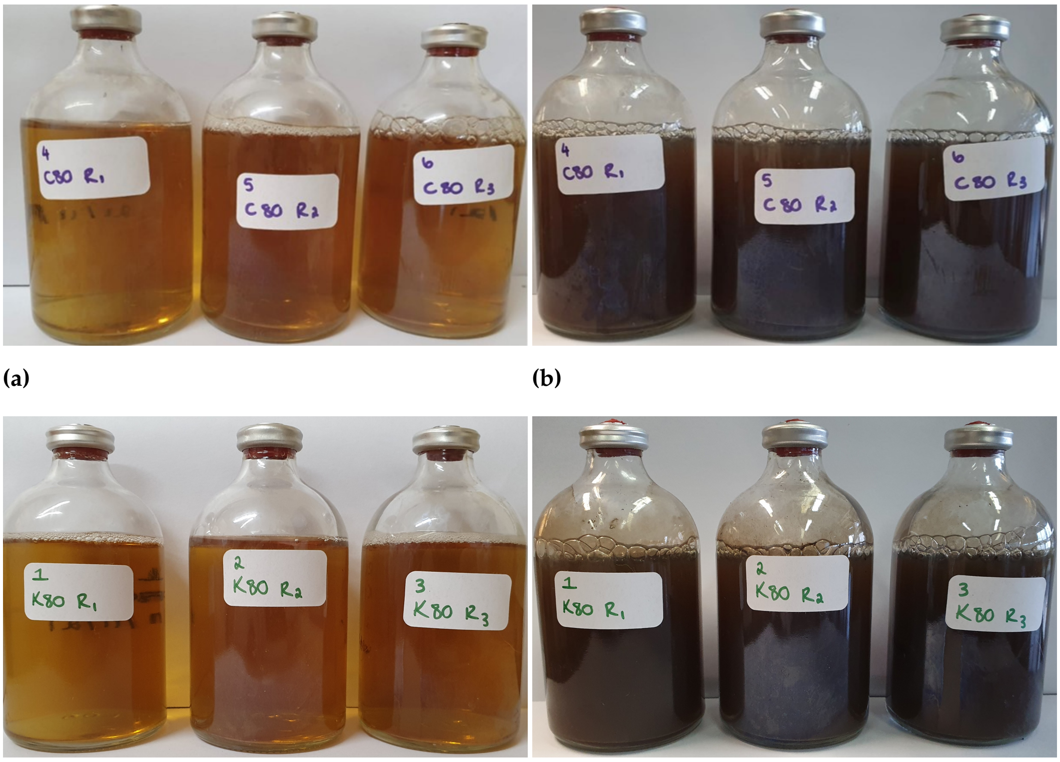

(a) **(b)** **(c)** **(d)**

Figure S3. The bioreactors of (a) *P. bifermentans* and (c) *K. pneumoniae* at 0 h and the rapid visual changes observed for (b) *P. bifermentans* and (d) *K. pneumoniae* after 17 h.

Table S1. The average percentage of Pb(II) removed from the reactors of each run at the final sampling time for the long duration study.

Strain	Time elapsed (h)	Pb(II) removed (%)
P. bifermentans	95	83.8
K. pneumoniae	93	100[1]
Microbial consortium	100	94.4

[1] Taken to be 100 % since values were too low to be detected by instrumentation

Table S2. The percentage removal of Pb(II) for each strain at varying initial Pb(II) concentrations at the final sampling time of 30 h.

Strain	Initial Pb(II) concentration (mg L^{-1})	Percentage of Pb(II) removed (%)
P. bifermentans	80	86.1 ± 4.61
P. bifermentans	250	55.6 ± 6.58
P. bifermentans	500	19.2 ± 32.5
K. pneumoniae	80	91.1 ± 0.302
K. pneumoniae	250	27.4 ± 12.4
K. pneumoniae	500	41.5 ± 6.59

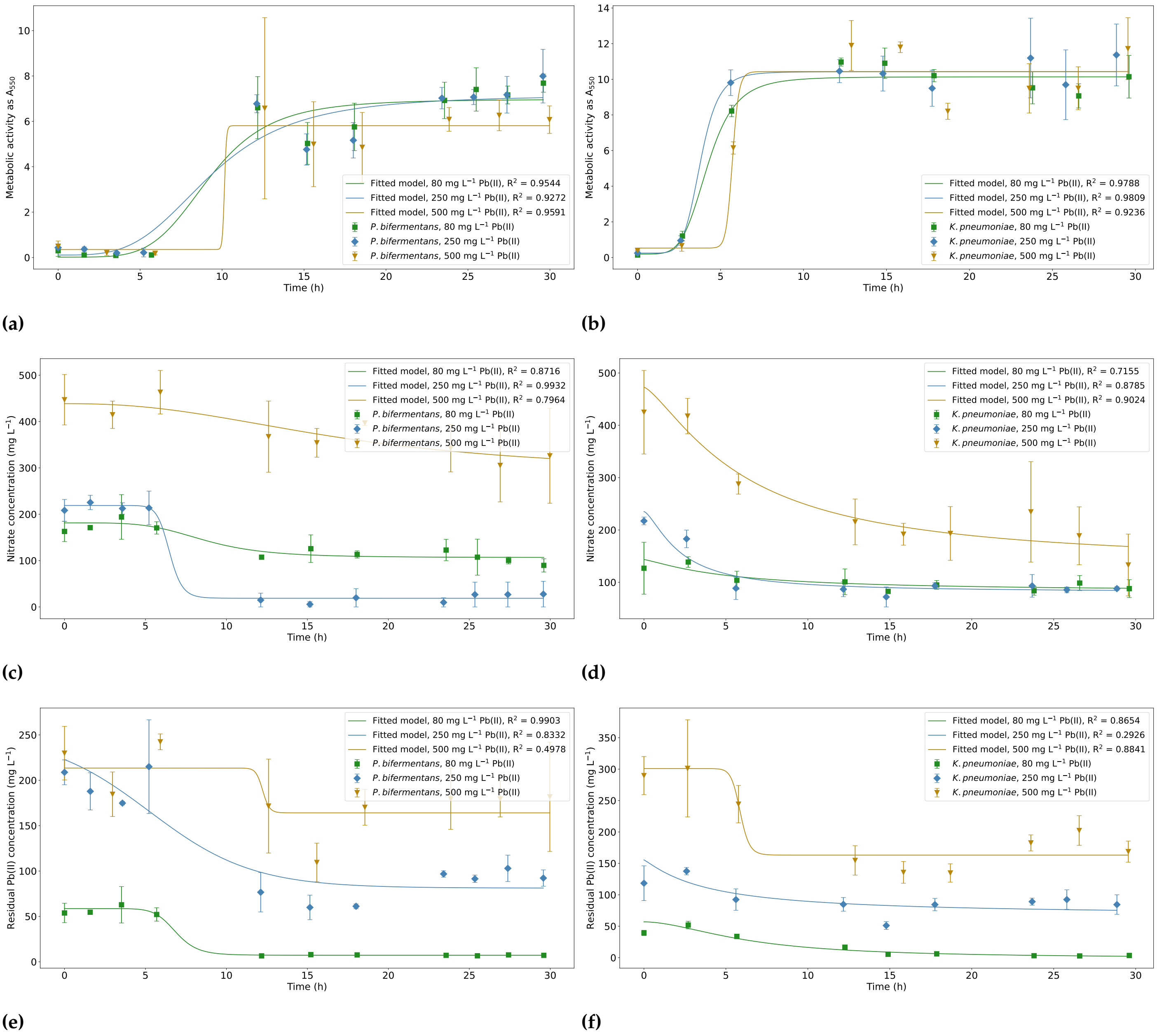

Figure S4. The fitted models using a four part sigmoidal curve for (a) metabolic activity of *P. bifermentans*, (b) metabolic activity of *K. pneumoniae*, (c) nitrate concentration of *P. bifermentans*, (d) nitrate concentration of *K. pneumoniae*, (e) residual Pb(II) concentration of *P. bifermentans* and (f) residual Pb(II) concentration of *K. pneumoniae*.

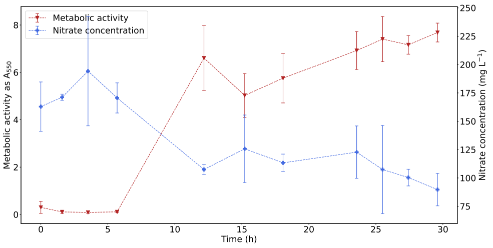

(a)

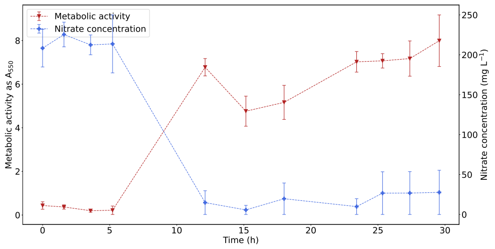

(b)

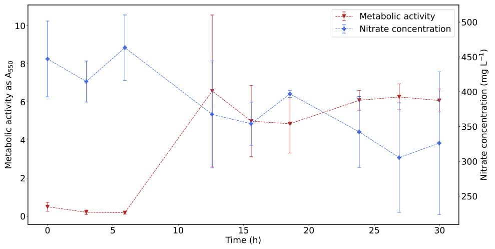

(c)

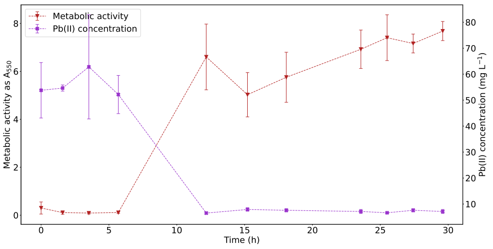

(d)

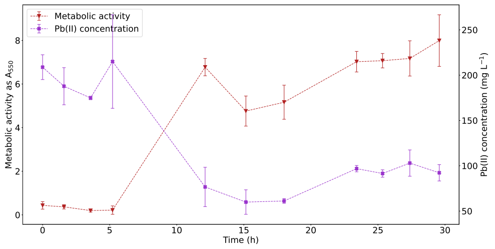

(e)

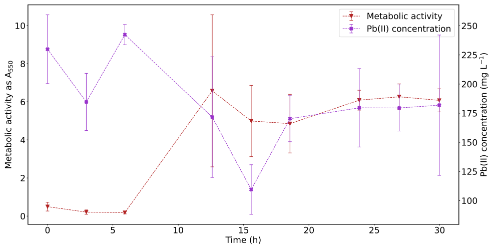

(f)

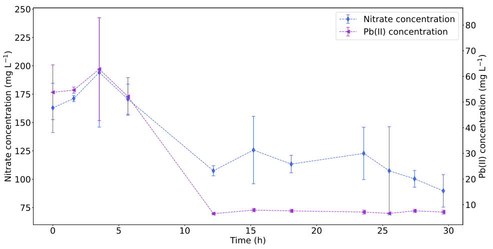

(g)

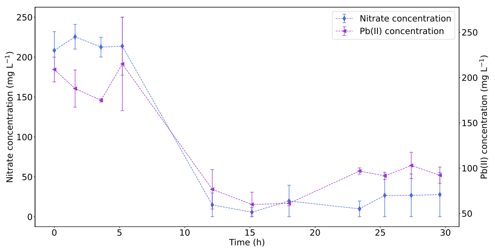

(h)

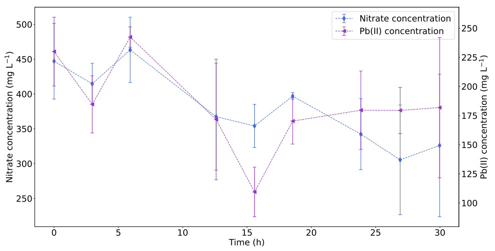

(i)

Figure S5. A comparison between metabolic activity at 550 nm and the nitrate concentration for (a) 80 mg L^{-1}, (b) 250 mg L^{-1}, (c) 500 mg L^{-1} initial Pb(II) concentration, a comparison between metabolic activity and Pb(II) concentration for (d) 80 mg L^{-1}, (e) 250 mg L^{-1}, (f) 500 mg L^{-1} initial Pb(II) concentration and a comparison between nitrate concentration and Pb(II) concentration of (g) 80 mg L^{-1}, (h) 250 mg L^{-1} and (i) 500 mg L^{-1} initial Pb(II) concentration for *P. bifermentans*.

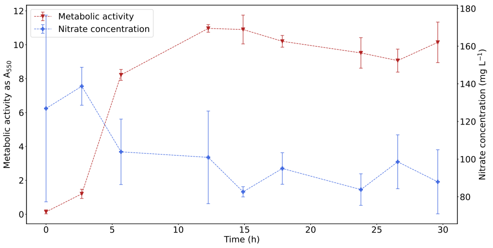

(a)

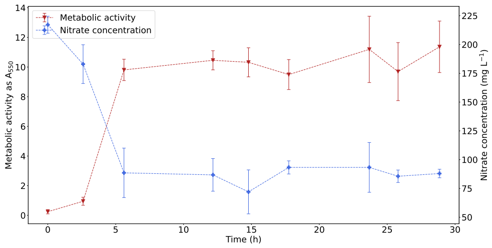

(b)

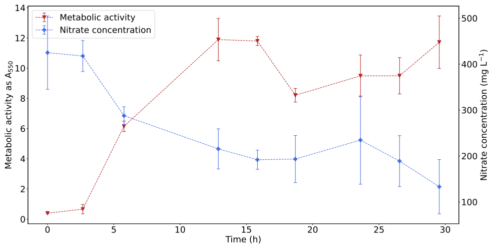

(c)

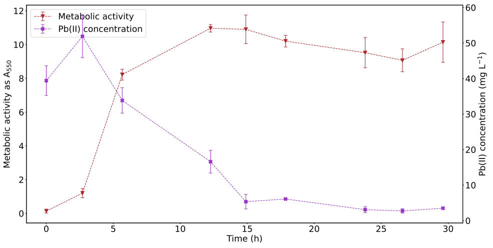

(d)

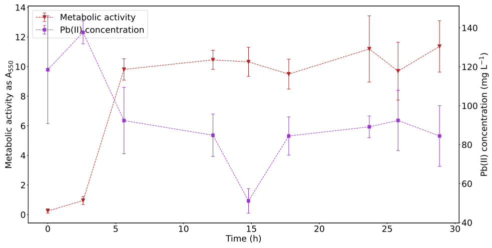

(e)

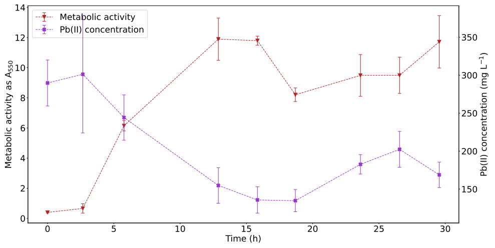

(f)

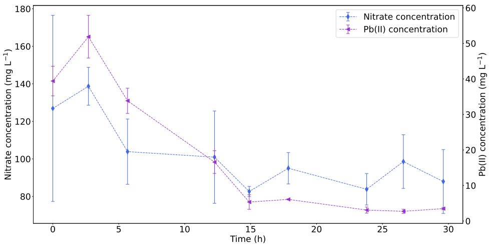

(g)

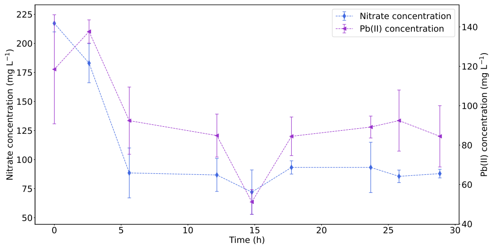

(h)

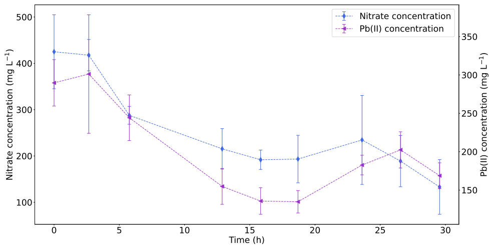

(i)

Figure S6. A comparison between metabolic activity at 550 nm and the nitrate concentration for (a) 80 mg L^{-1}, (b) 250 mg L^{-1}, (c) 500 mg L^{-1} initial Pb(II) concentration, a comparison between metabolic activity and Pb(II) concentration for (d) 80 mg L^{-1}, (e) 250 mg L^{-1}, (f) 500 mg L^{-1} initial Pb(II) concentration and a comparison between nitrate concentration and Pb(II) concentration of (g) 80 mg L^{-1}, (h) 250 mg L^{-1} and (i) 500 mg L^{-1} initial Pb(II) concentration for *K. pneumoniae*.

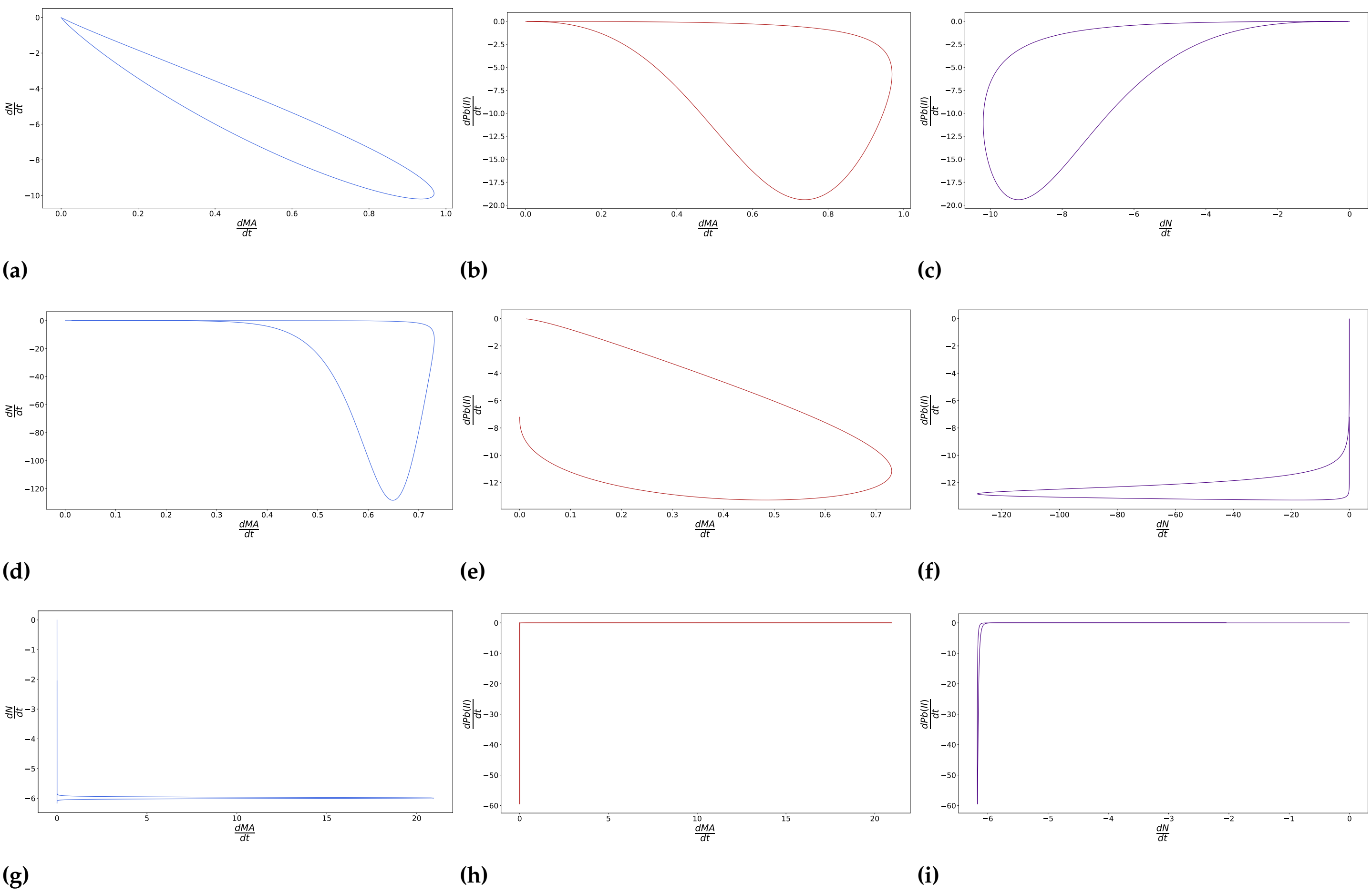

Figure S7. The comparison between the first derivative curves of *P. bifermentans* for (a) metabolic activity and nitrate concentration, (b) metabolic activity and lead concentration, (c) nitrate and lead concentration for 80 mg L^{-1} initial Pb(II) concentration, (d) metabolic activity and nitrate concentration, (e) metabolic activity and lead concentration, (f) nitrate and lead concentration for 250 mg L^{-1} initial Pb(II) concentration, (g) metabolic activity and nitrate concentration, (h) metabolic activity and lead concentration, (i) nitrate and lead concentration for 500 mg L^{-1} initial Pb(II) concentration.

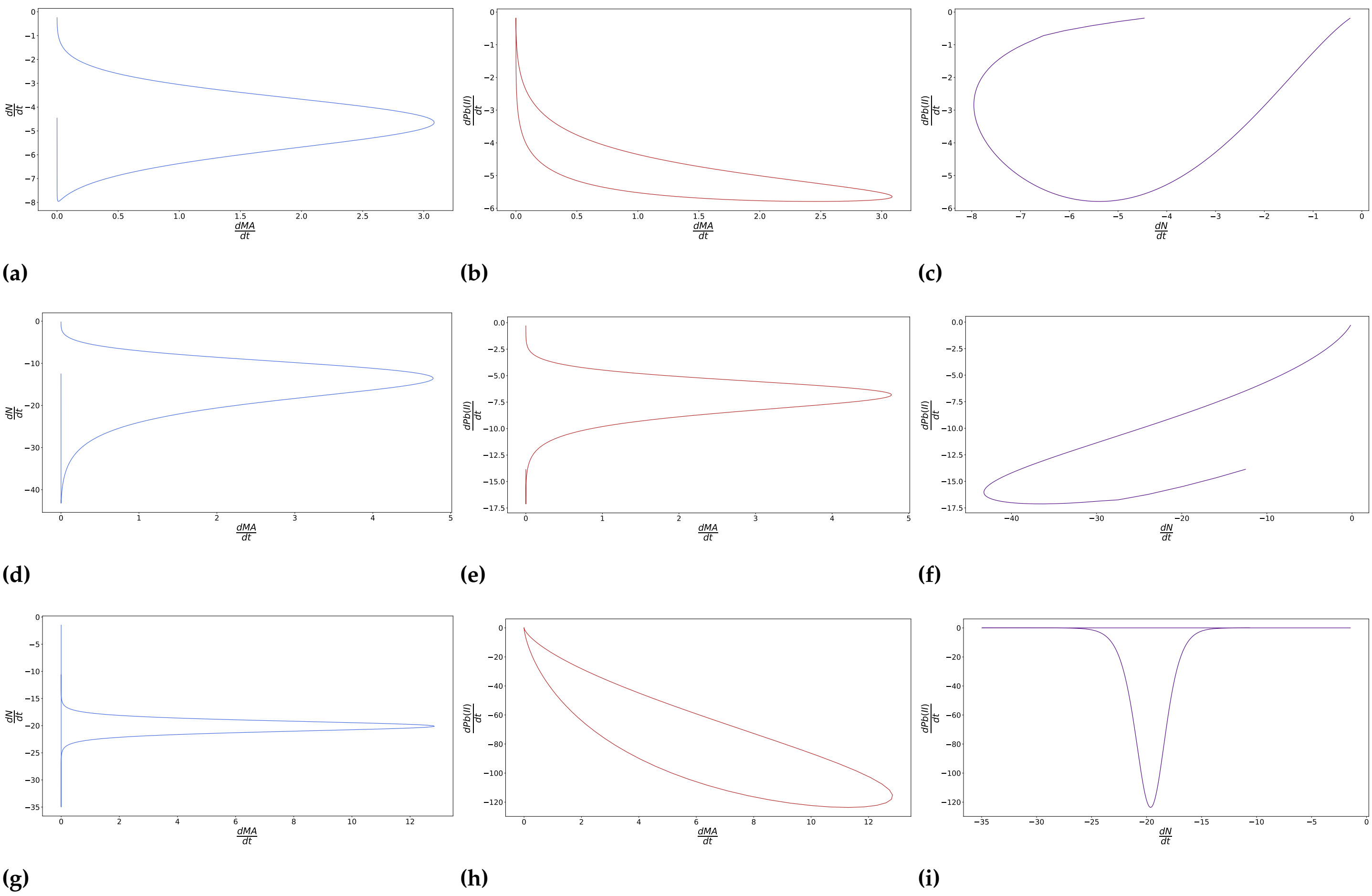

Figure S8. The comparison between the first derivative curves of *K. pneumoniae* for (a) metabolic activity and nitrate concentration, (b) metabolic activity and lead concentration, (c) nitrate and lead concentration for 80 mg L^{-1} initial Pb(II) concentration, (d) metabolic activity and nitrate concentration, (e) metabolic activity and lead concentration, (f) nitrate and lead concentration for 250 mg L^{-1} initial Pb(II) concentration, (g) metabolic activity and nitrate concentration, (h) metabolic activity and lead concentration, (i) nitrate and lead concentration for 500 mg L^{-1} initial Pb(II) concentration.

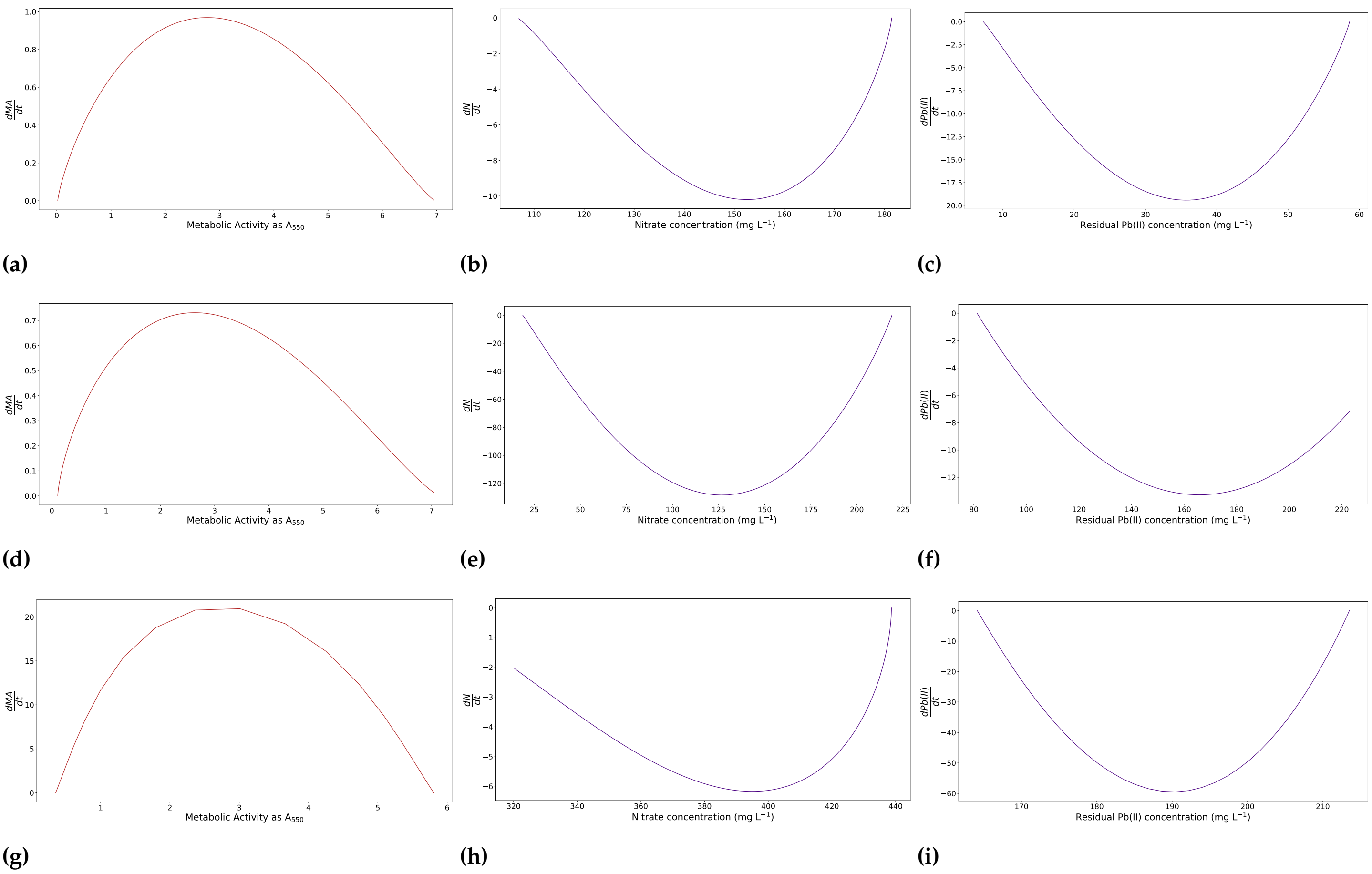

Figure S9. The comparison between the metabolic activity and the rate of change in metabolic activity for *P. bifermentans* with (a) 80 mg L^{-1}, (d) 250 mg L^{-1} and (g) 500 mg L^{-1} initial Pb(II) concentration. The comparison between the nitrate concentration and the rate of change in nitrate concentration for *P. bifermentans* with (b) 80 mg L^{-1}, (e) 250 mg L^{-1} and (h) 500 mg L^{-1} initial Pb(II) concentration. The comparison between the residual Pb(II) concentration and the rate of change in Pb(II) concentration for *P. bifermentans* with (c) 80 mg L^{-1}, (f) 250 mg L^{-1} and (i) 500 mg L^{-1} initial Pb(II) concentration

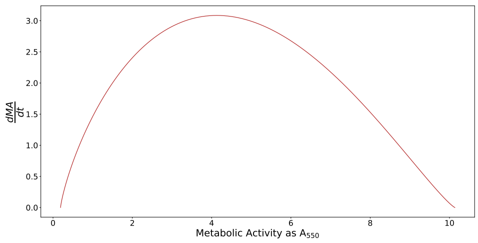

(a)

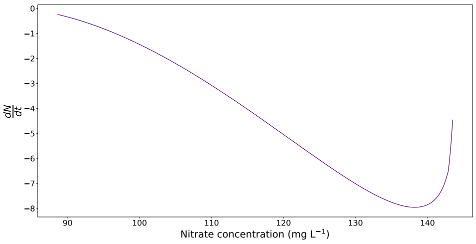

(b)

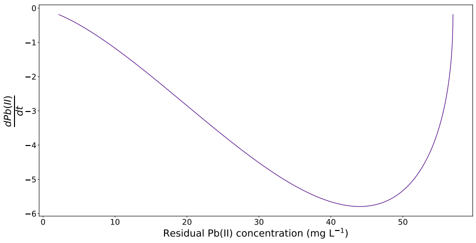

(c)

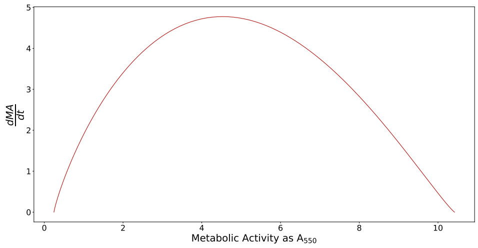

(d)

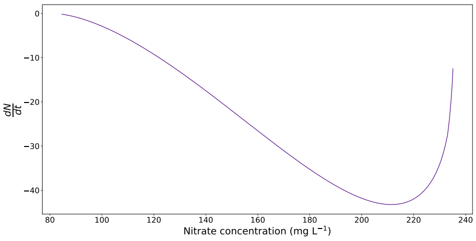

(e)

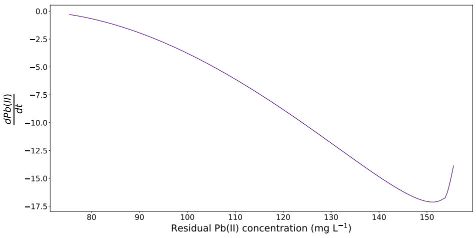

(f)

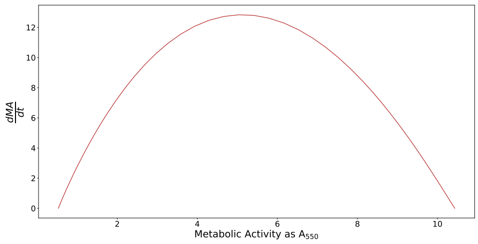

(g)

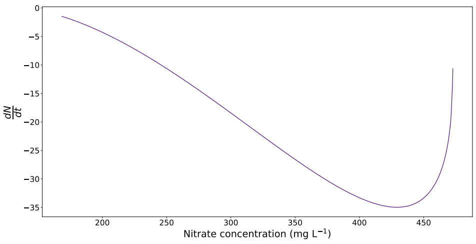

(h)

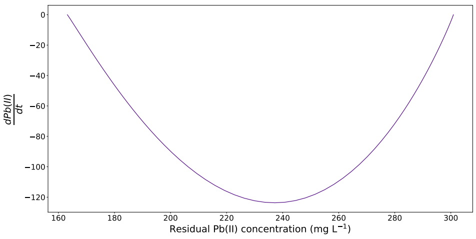

(i)

Figure S10. The comparison between the metabolic activity and the rate of change in metabolic activity for *K. pneumoniae* with (a) 80 mg L^{-1}, (d) 250 mg L^{-1} and (g) 500 mg L^{-1} initial Pb(II) concentration. The comparison between the nitrate concentration and the rate of change in nitrate concentration for *K. pneumoniae* with (b) 80 mg L^{-1}, (e) 250 mg L^{-1} and (h) 500 mg L^{-1} initial Pb(II) concentration. The comparison between the residual Pb(II) concentration and the rate of change in Pb(II) concentration for *K. pneumoniae* with (c) 80 mg L^{-1}, (f) 250 mg L^{-1} and (i) 500 mg L^{-1} initial Pb(II) concentration

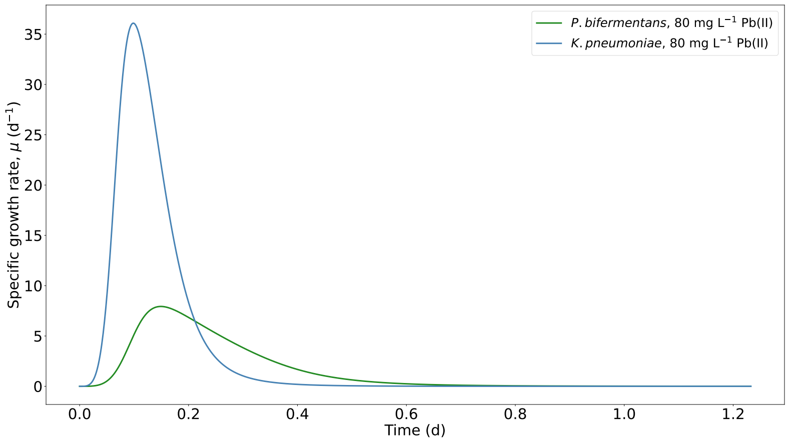

(a)

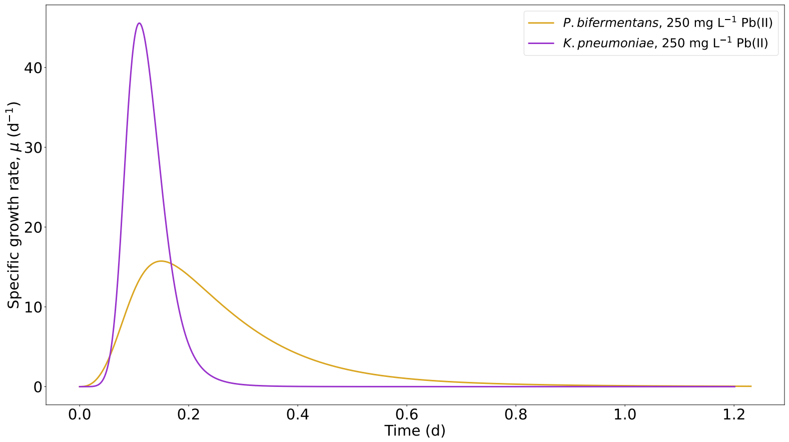

(b)

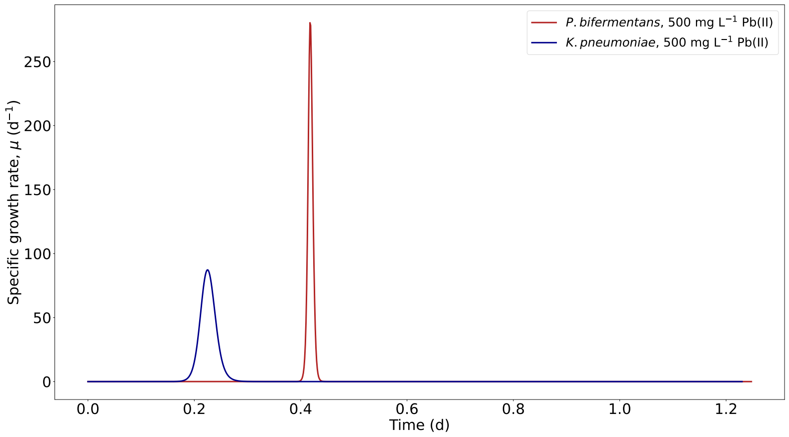

(c)

Figure S11. The specific growth rate, μ, over time for *P. bifermentans* and *K. pneumoniae* with (a) 80 mg L^{-1}, (b) 250 mg L^{-1} and (c) 500 mg L^{-1} initial Pb(II) concentration.

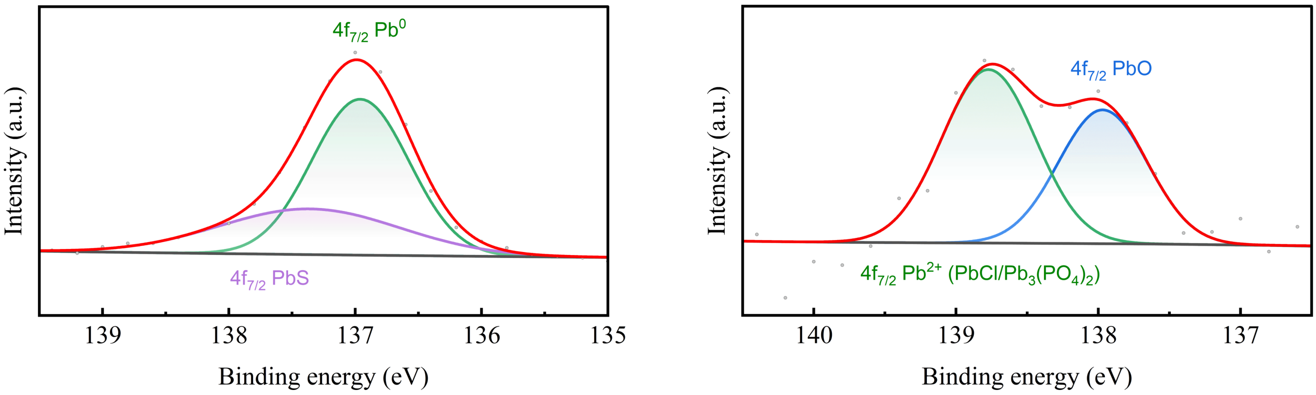

(a) **(b)**

Figure S12. The XPS profiles of the Pb-species for samples containing 80 mg L^{-1} initial Pb(II) concentration with (a) *P. bifermentans* and (b) *K. pneumoniae*.

2. Statistical inference analysis for Section 3.3.3

A statistical inference analysis was performed to determine if the initial concentration of nitrates for the experimental conditions of varying initial Pb(II) concentrations and varying bacterial strains, differs [1]. If the nitrate concentrations measured at the start of the experiment show insignificant differences then it can be deduced that the experiments contained the same initial Pb(II) concentrations since the main source of nitrates in the reactors was $Pb(NO_3)_2$. The t-test statistic was calculated as

$$t = \frac{\overline{X}_1 - \overline{X}_2}{S_p \sqrt{\frac{1}{n_1} + \frac{1}{n_2}}} \tag{1}$$

where $\overline{X}_1$ and $\overline{X}_2$ are the average of the three runs of the two populations, n_1 and n_2 are the sample sizes which are equal to three for all analysis and S_p is the pooled estimate of the common standard deviation given by

$$S_p = \sqrt{\frac{(n_1 - 1)s_1^2 + (n_2 - 1)s_2^2}{n_1 + n_2 - 2}} \tag{2}$$

where s_1 and s_2 are the sample standard deviations of the two populations.The null and alternative hypothesis used were

$$H_0 : \mu_1 = \mu_2 \tag{3}$$

and

$$H_1 : \mu_1 \neq \mu_2 \tag{4}$$

where μ is the population mean for population 1 and 2. The degrees of freedom are given by

$$df = n_1 + n_2 - 2 \tag{5}$$

and since n_1 and n_2 are both always equal to three because three replicates of each experiment were done, the degrees of freedom was always equal to four. A confidence interval of 95 % was used and the critical value of the t distribution at this confidence level and at four degrees of freedom was equal to 2.776. The null hypothesis can therefore be rejected if:

$$t > 2.776 \text{ or } t < -2.776$$

The value of the t statistic and the resulting conclusions from the statistical inference analysis are shown in Table S3. The null hypothesis is not rejected for any calculated t value and it can be concluded that there is no statistically significant difference in the initial nitrate concentration between experimental conditions.

Table S3. The calculated t statistics of the inference analysis in Section 3.3.3

**Initial Pb(II) concentration (mg L^{-1})**	**Populations to be compared**	t
80	*P. bifermentans* and *K. pneumoniae*	-0.3324
	P. bifermentans and microbial consortium	-1.341
	K. pneumoniae and microbial consortium	-0.6174
250	*P. bifermentans* and *K. pneumoniae*	-0.6207
500	*P. bifermentans* and *K. pneumoniae*	-0.2274
	P. bifermentans and microbial consortium	-0.5171
	K. pneumoniae and microbial consortium	-0.4058

References

1. D'Agostino Sr, R.B.; Sullivan, L.M.; Beiser, A.S. *Introductory Applied Biostatistics*, 1st ed.; Publisher: Cengage Learning Location, Belmont, CA, USA, 2006; pp. 239.
