# Supplementary figures and images for: Microbial Precipitation of Pb(II) with Wild Strains of Paraclostridium bifermentans and Klebsiella pneumoniae Isolated from an Industrially Obtained Microbial Consortium
Source: Int J Mol Sci. 2022 Oct 14;23(20):12255. doi: 10.3390/ijms232012255 (PMC9603858; doi:10.3390/ijms232012255)

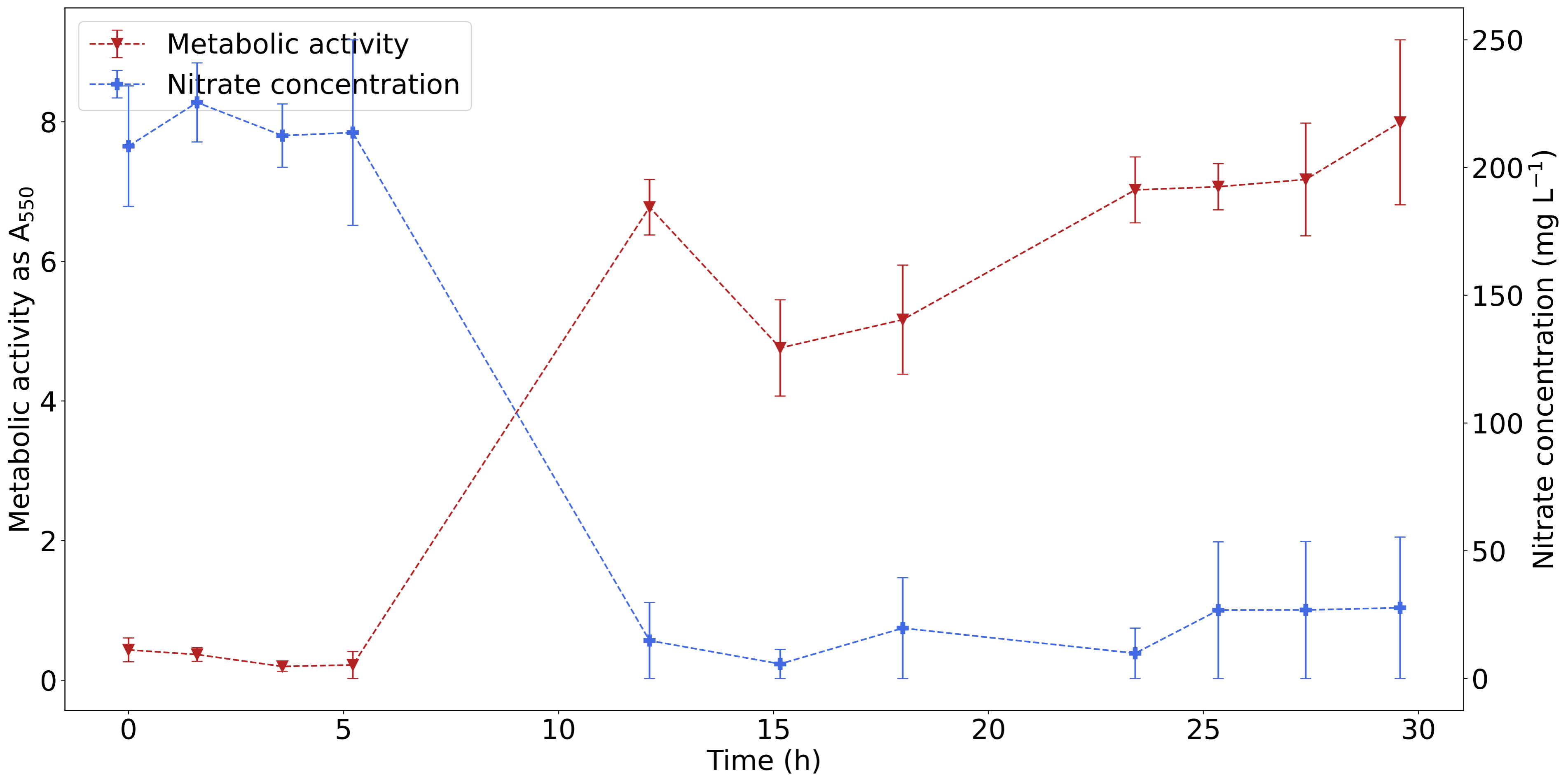

Supplement: Supplementary file 1 [file ijms-23-12255-s001.zip › Definitions/C250_MA_N.pdf]

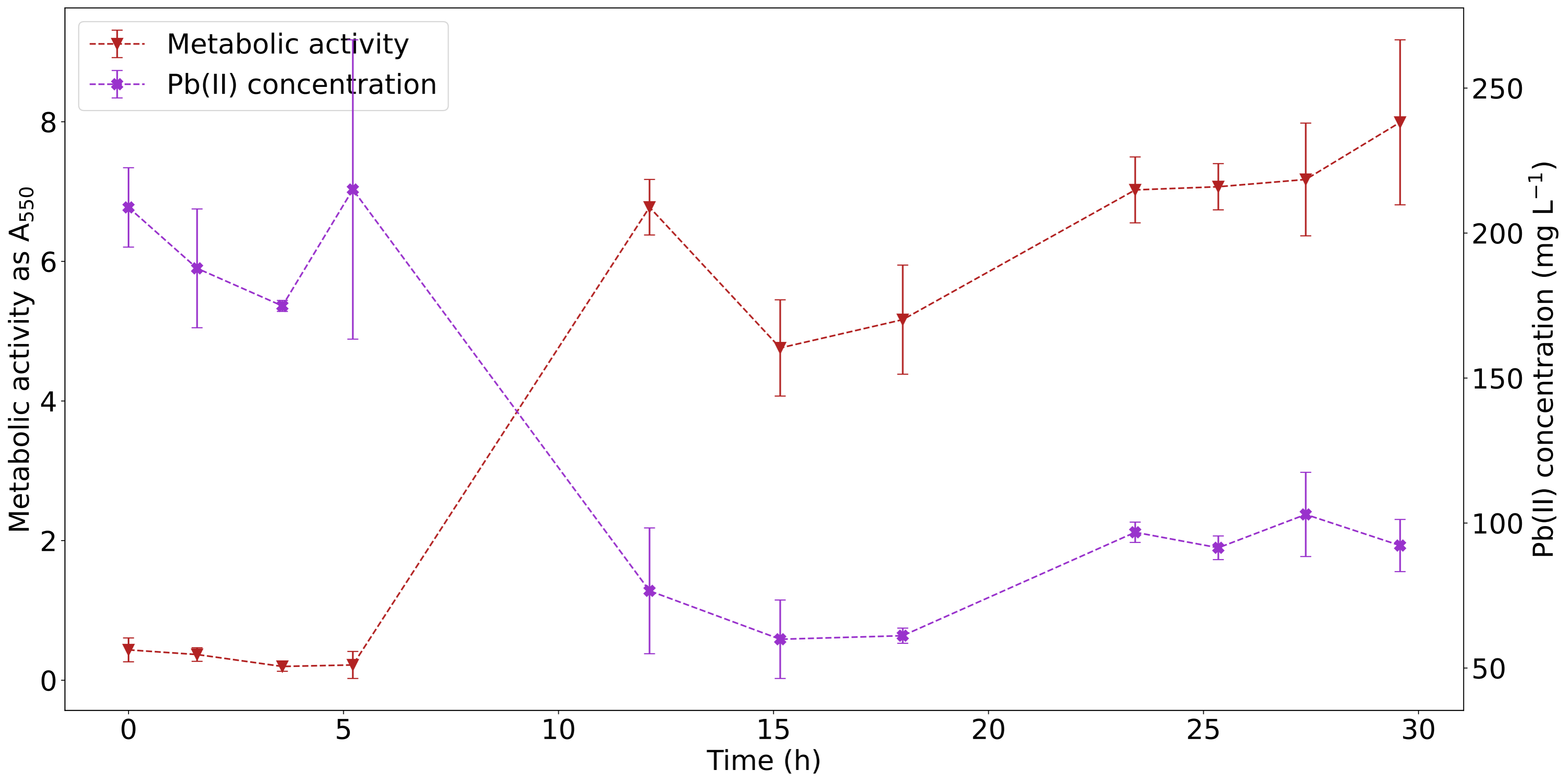

Supplement: Supplementary file 1 [file ijms-23-12255-s001.zip › Definitions/C250_MA_Pb.pdf]

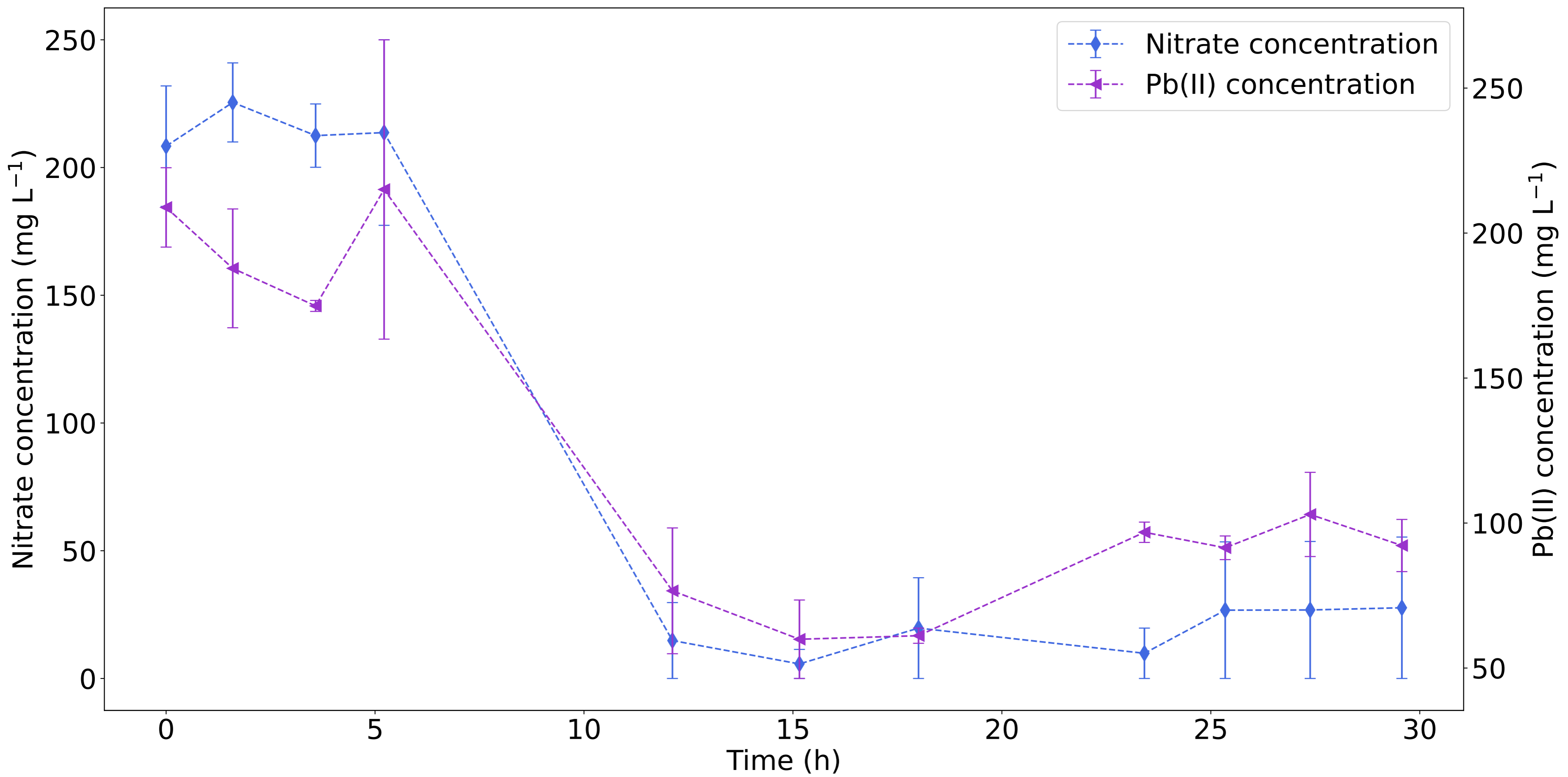

Supplement: Supplementary file 1 [file ijms-23-12255-s001.zip › Definitions/C250_N_Pb.pdf]

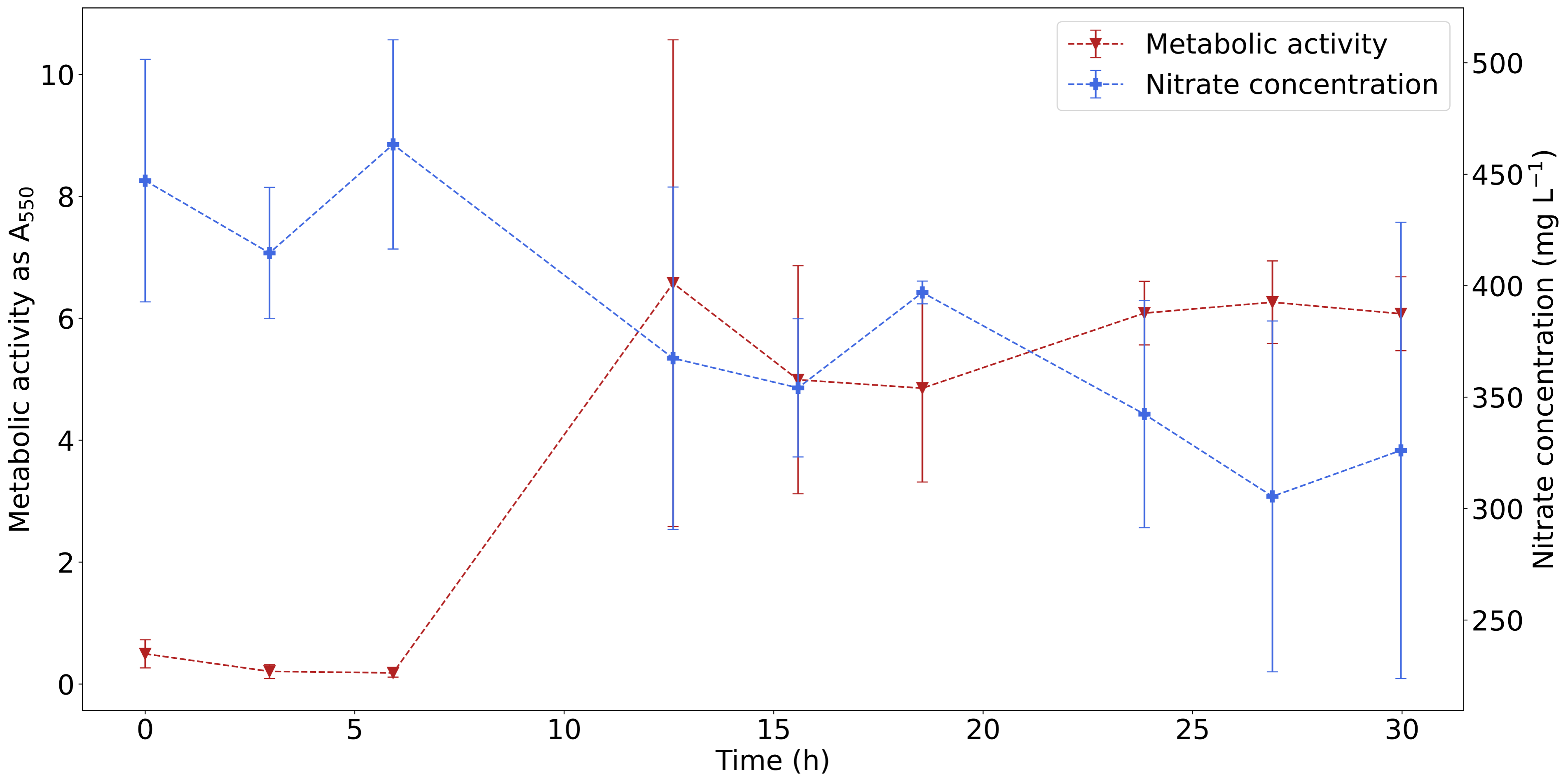

Supplement: Supplementary file 1 [file ijms-23-12255-s001.zip › Definitions/C500_MA_N.pdf]

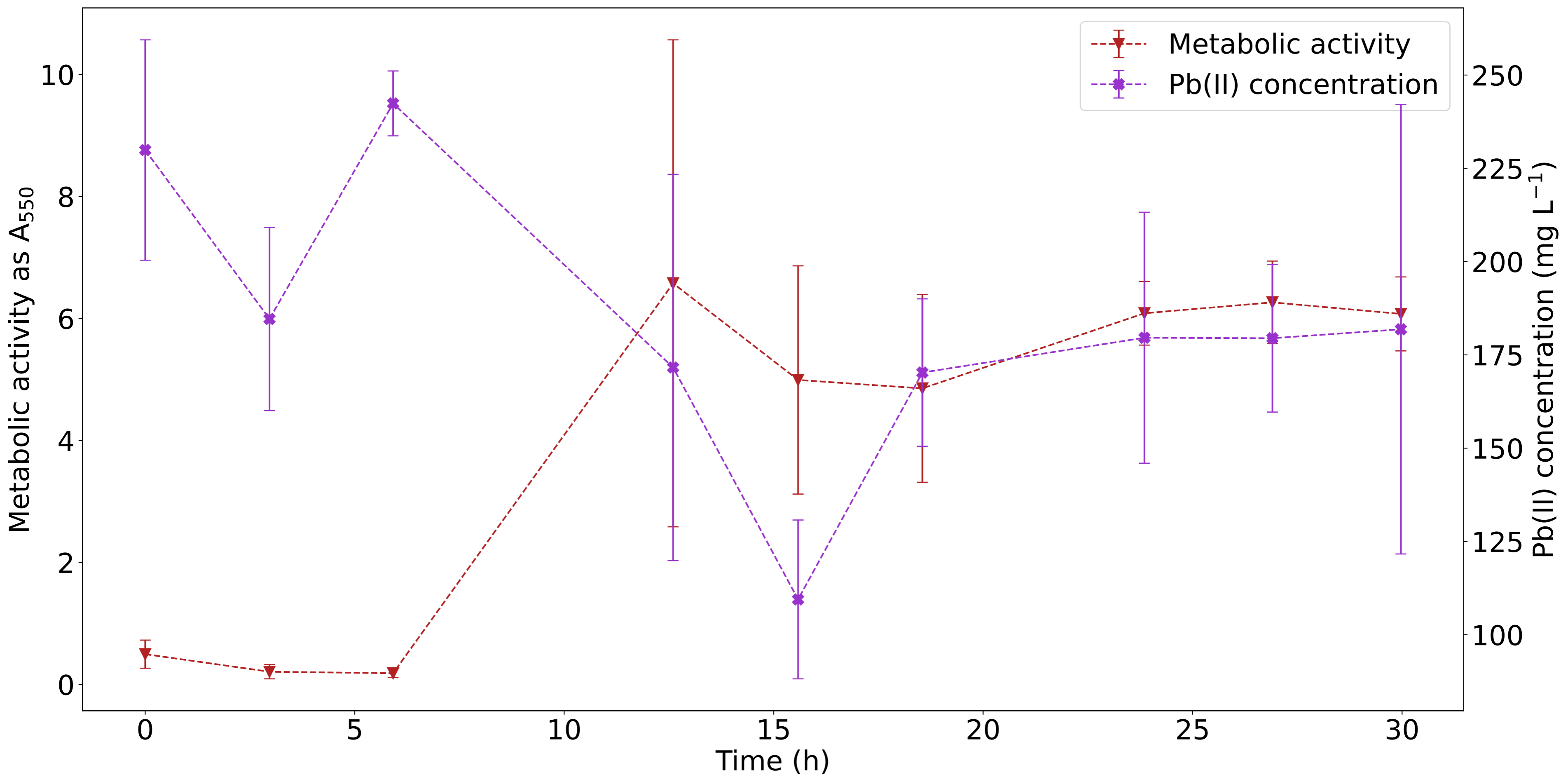

Supplement: Supplementary file 1 [file ijms-23-12255-s001.zip › Definitions/C500_MA_Pb.pdf]

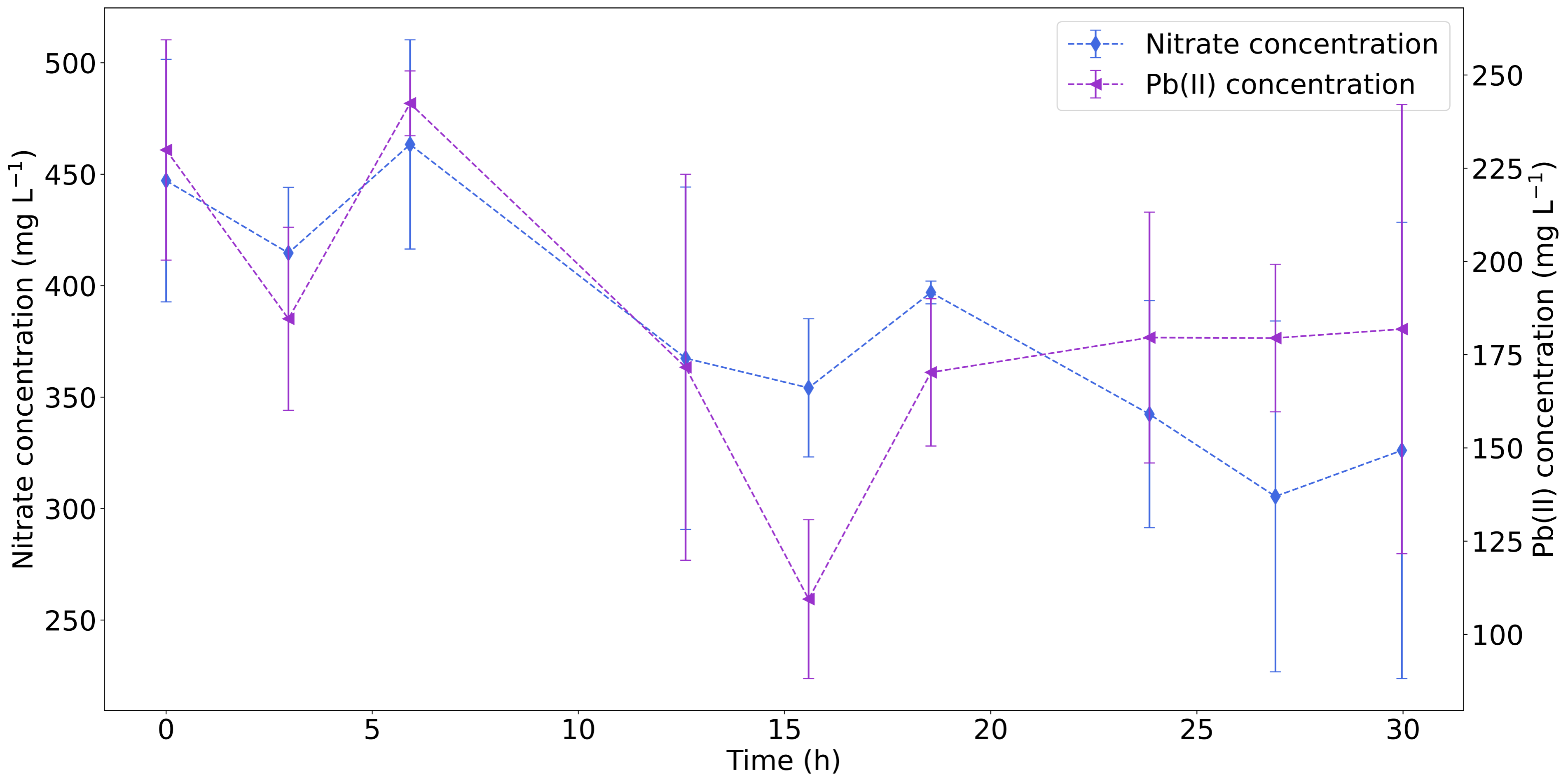

Supplement: Supplementary file 1 [file ijms-23-12255-s001.zip › Definitions/C500_N_Pb.pdf]

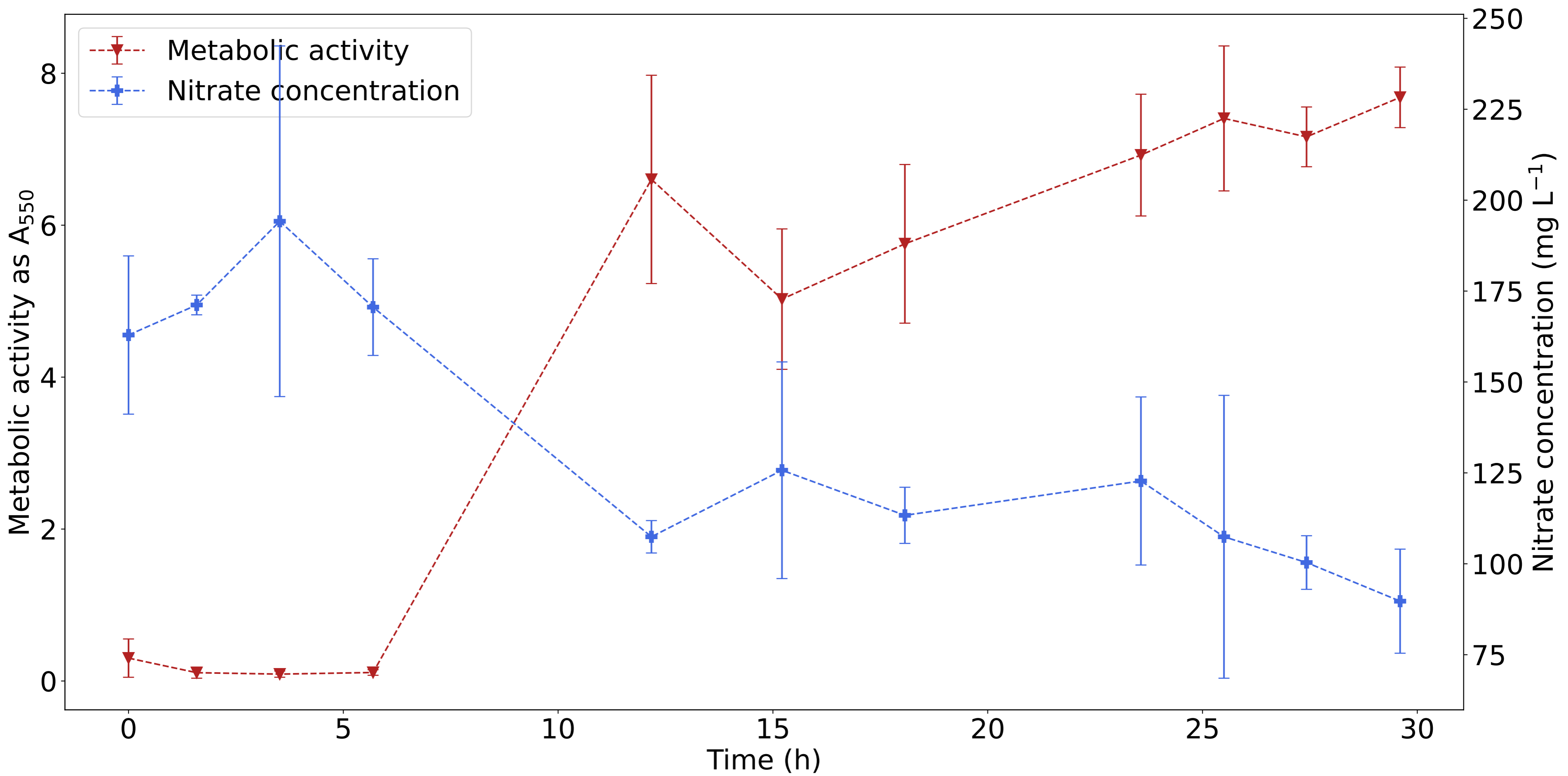

Supplement: Supplementary file 1 [file ijms-23-12255-s001.zip › Definitions/C80_MA_N.pdf]

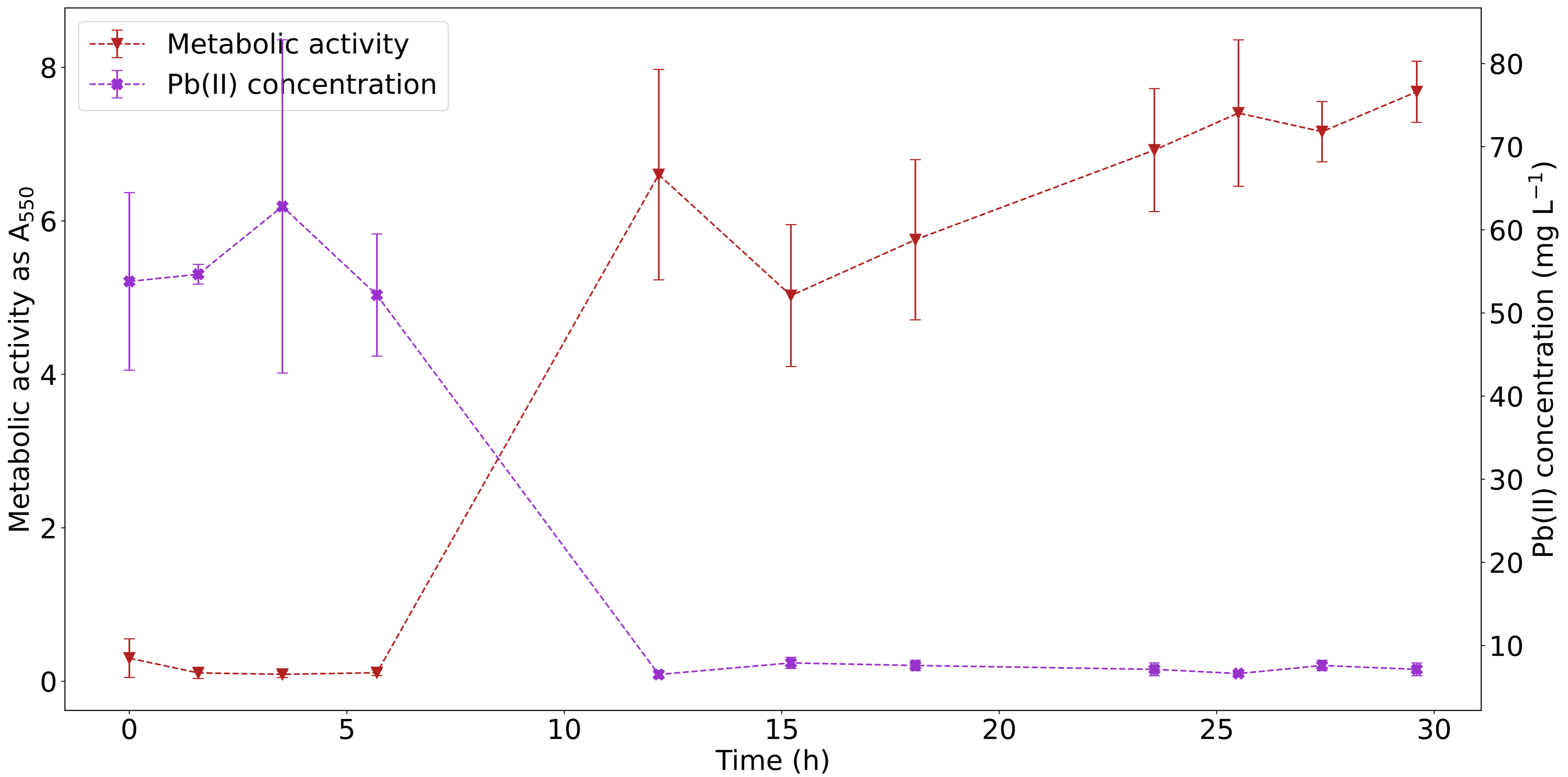

Supplement: Supplementary file 1 [file ijms-23-12255-s001.zip › Definitions/C80_MA_Pb.pdf]

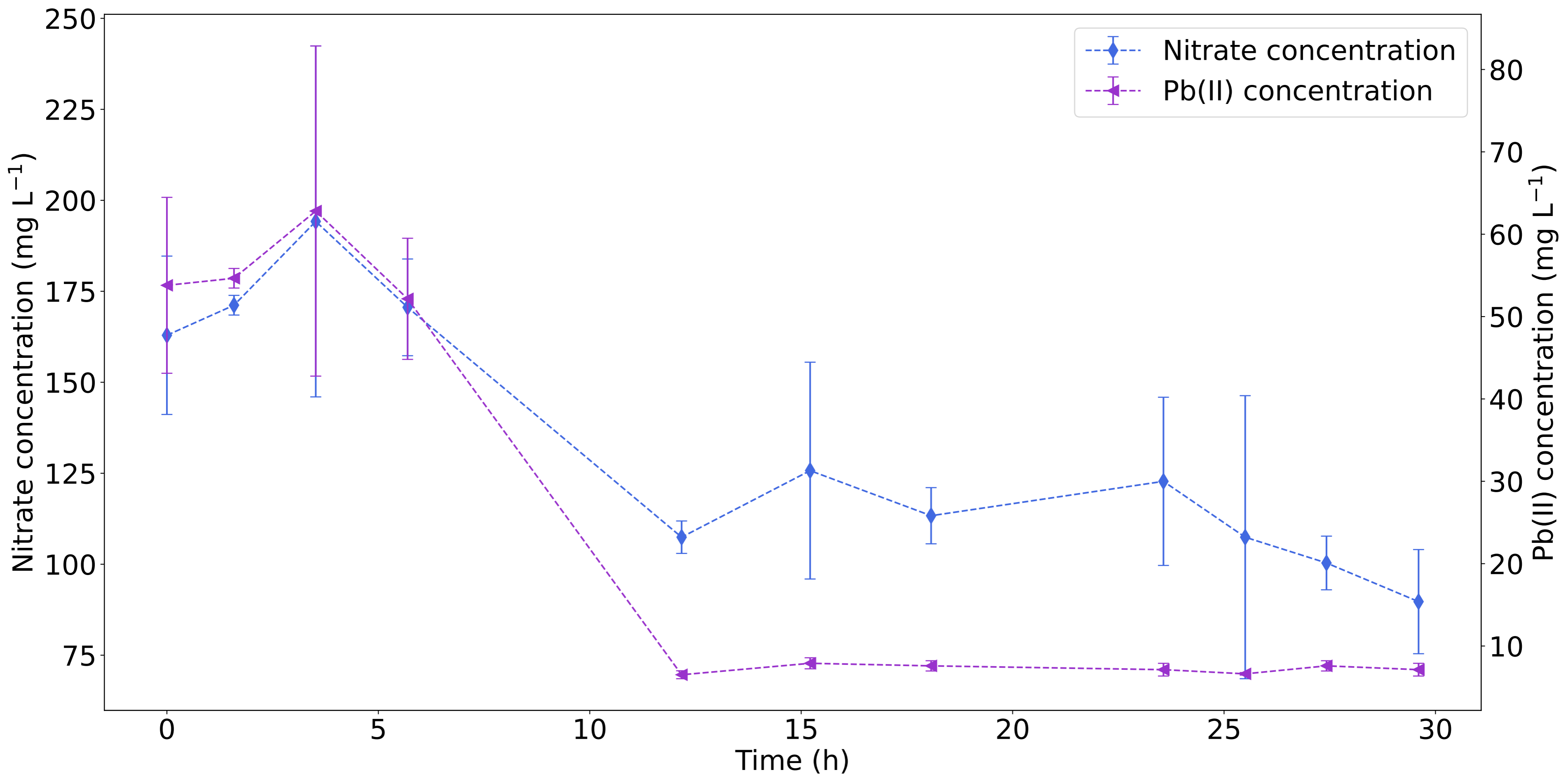

Supplement: Supplementary file 1 [file ijms-23-12255-s001.zip › Definitions/C80_N_Pb.pdf]

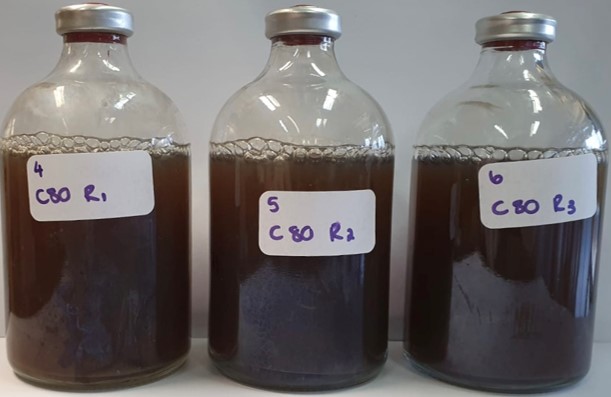

Supplement: Supplementary file 1 [file ijms-23-12255-s001.zip › Definitions/C80_TF.jpg]

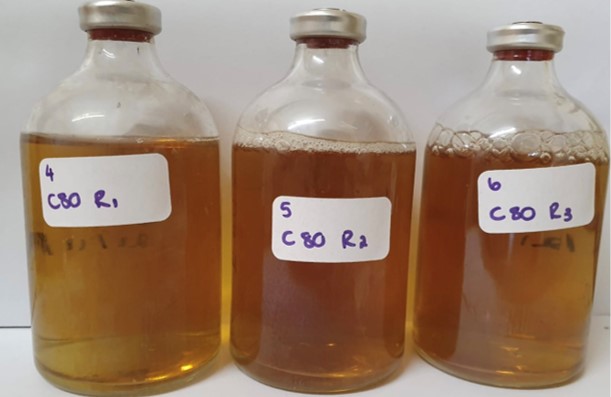

Supplement: Supplementary file 1 [file ijms-23-12255-s001.zip › Definitions/C80_TI.jpg]

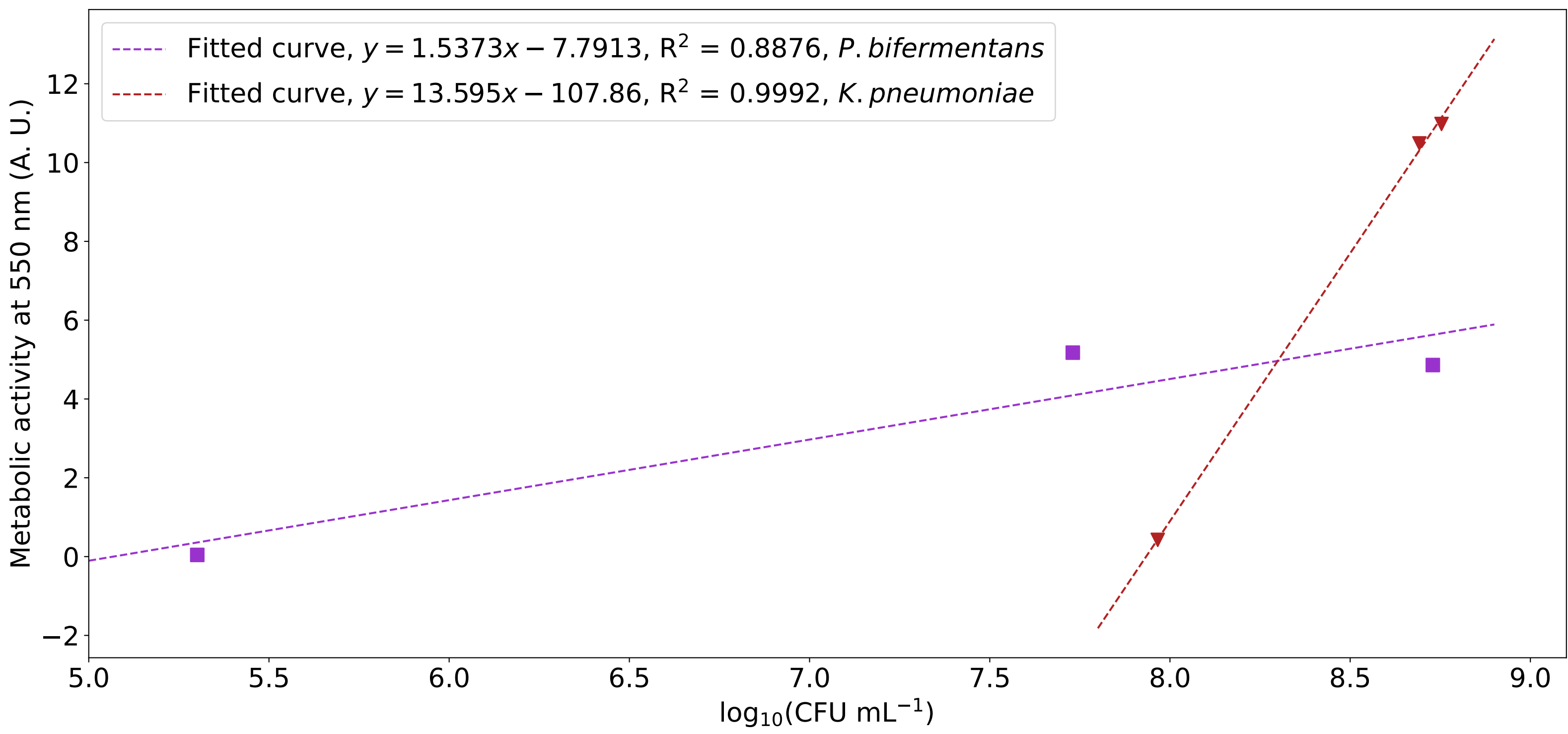

Supplement: Supplementary file 1 [file ijms-23-12255-s001.zip › Definitions/CFU_MA_K_P_Chap5.pdf]

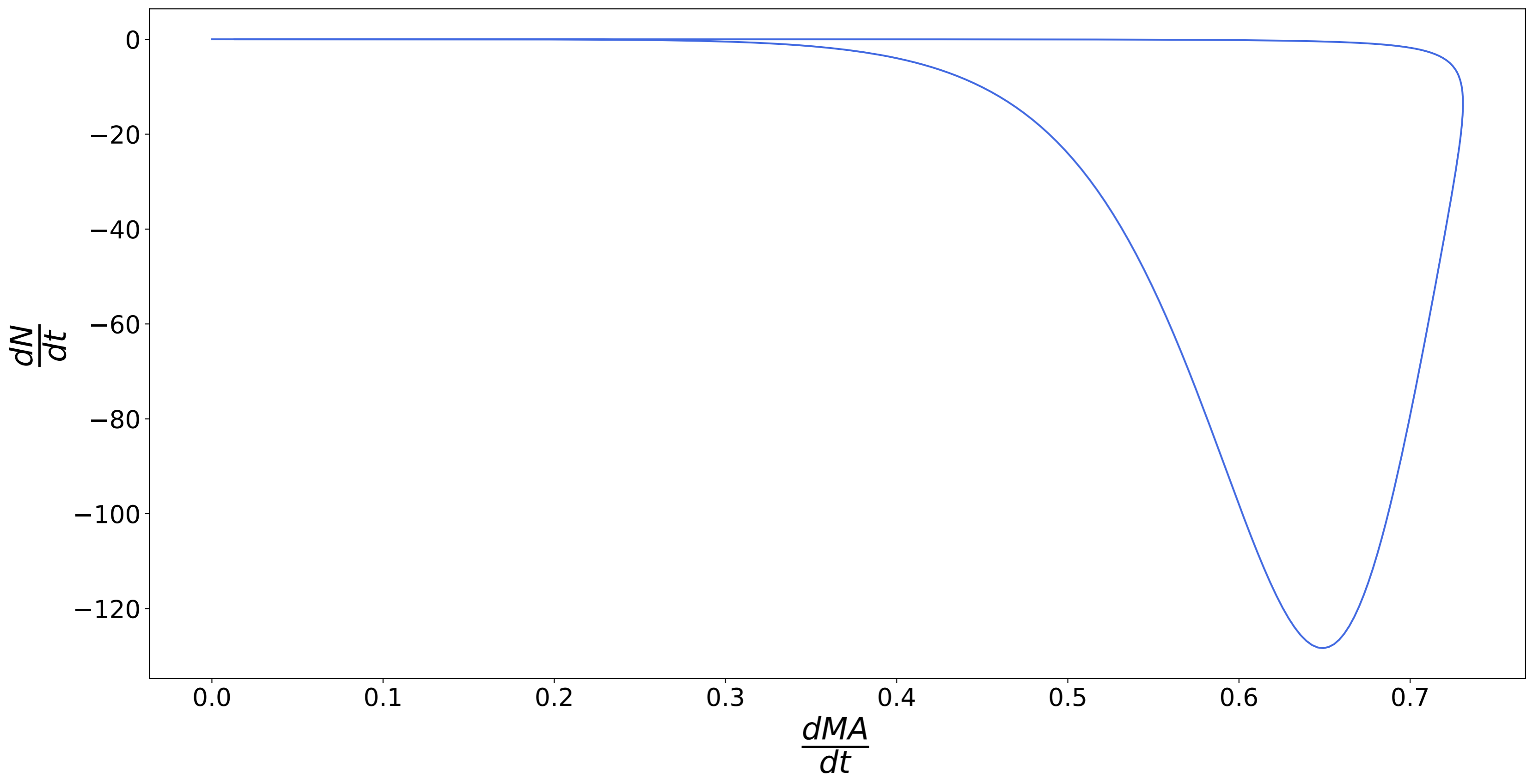

Supplement: Supplementary file 1 [file ijms-23-12255-s001.zip › Definitions/Derivatives_C250_MA_N.pdf]

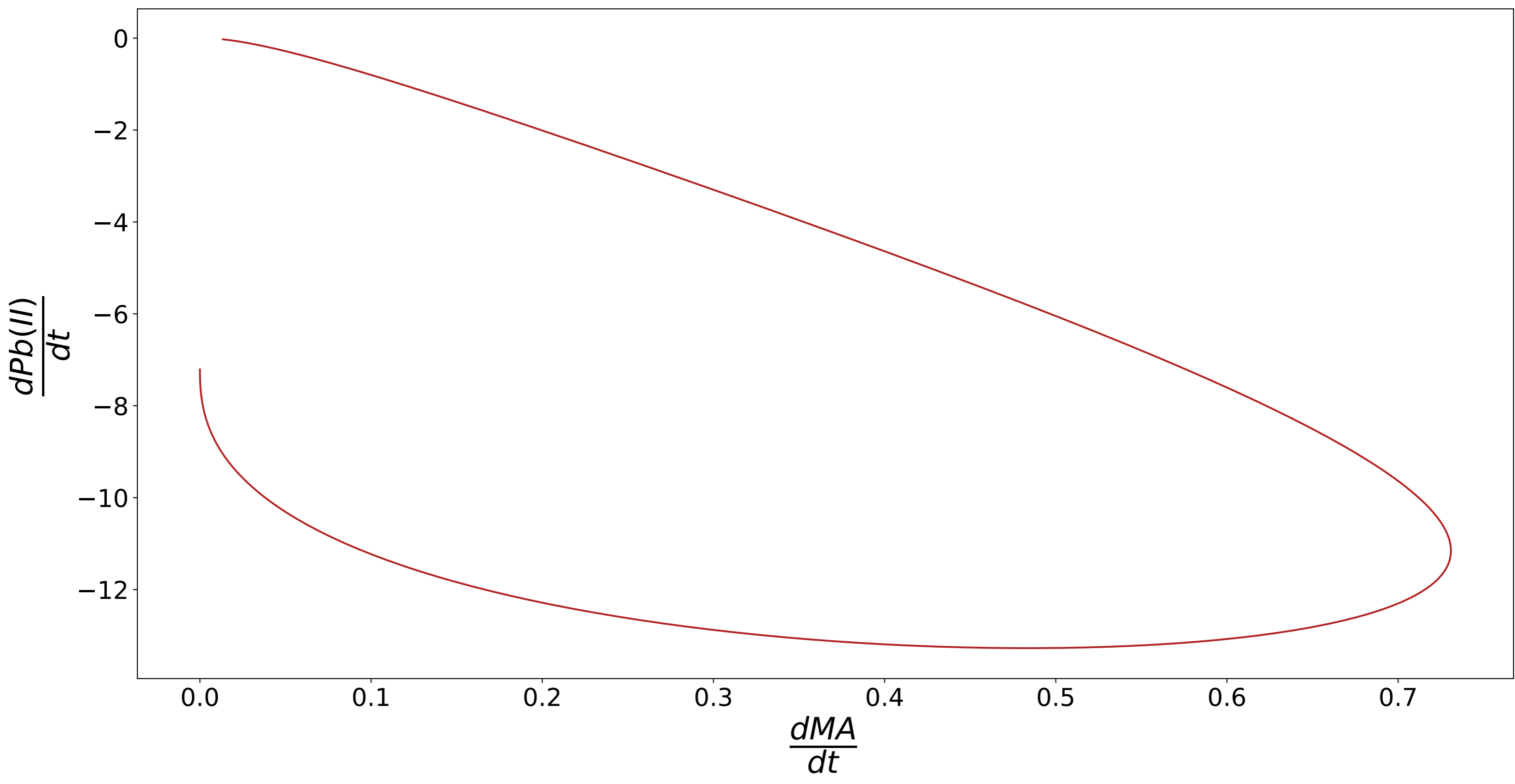

Supplement: Supplementary file 1 [file ijms-23-12255-s001.zip › Definitions/Derivatives_C250_MA_Pb.pdf]

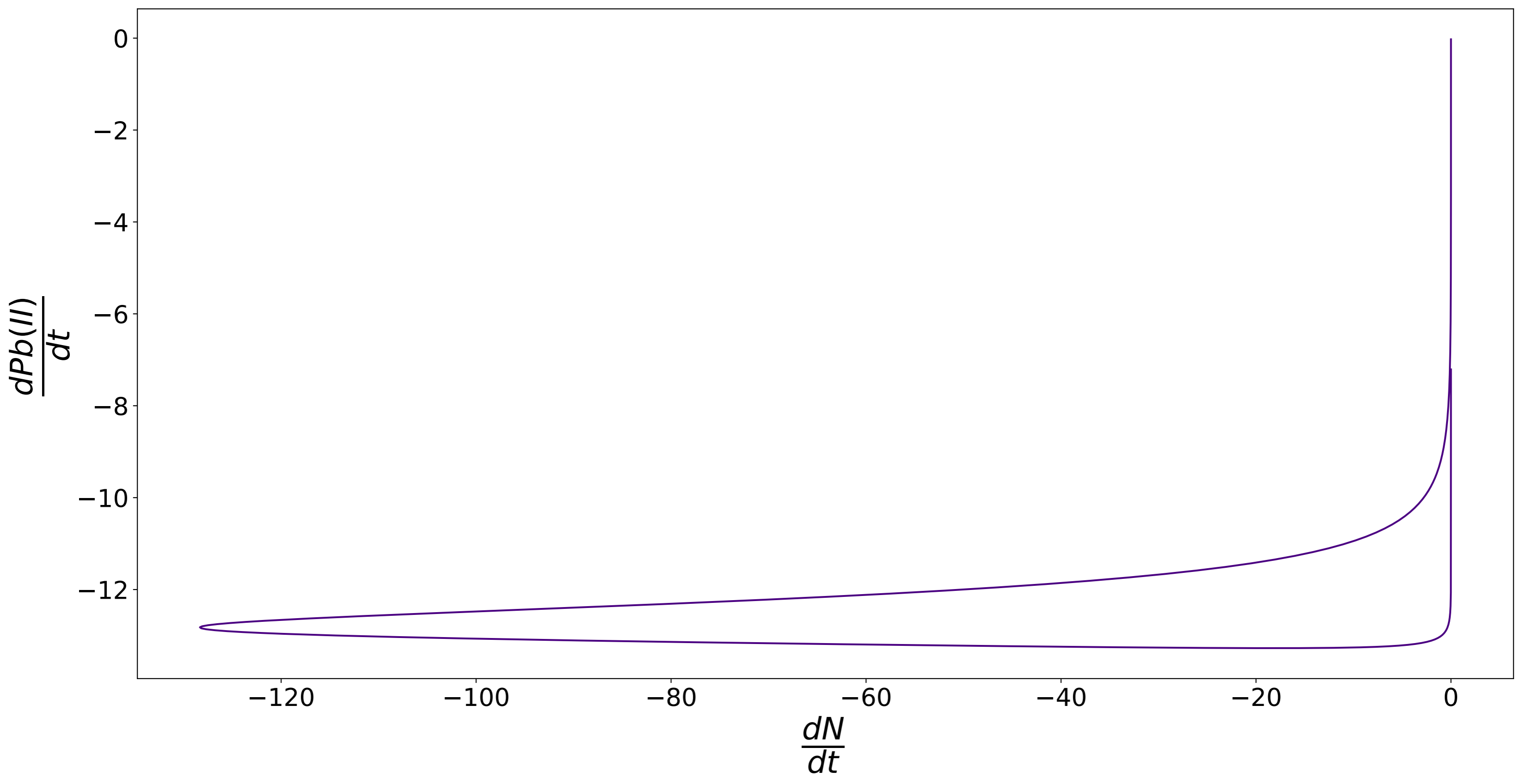

Supplement: Supplementary file 1 [file ijms-23-12255-s001.zip › Definitions/Derivatives_C250_N_Pb.pdf]

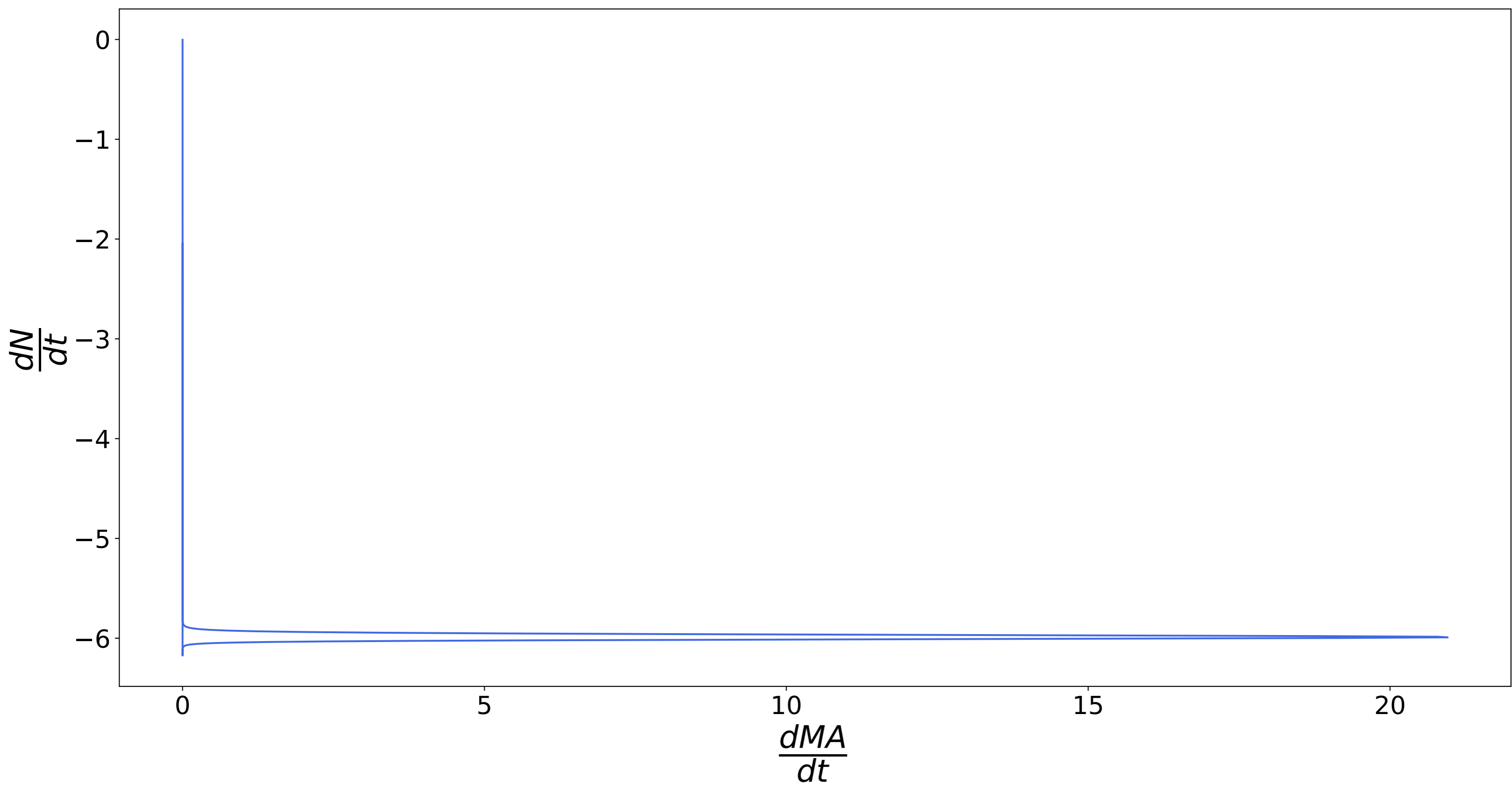

Supplement: Supplementary file 1 [file ijms-23-12255-s001.zip › Definitions/Derivatives_C500_MA_N.pdf]

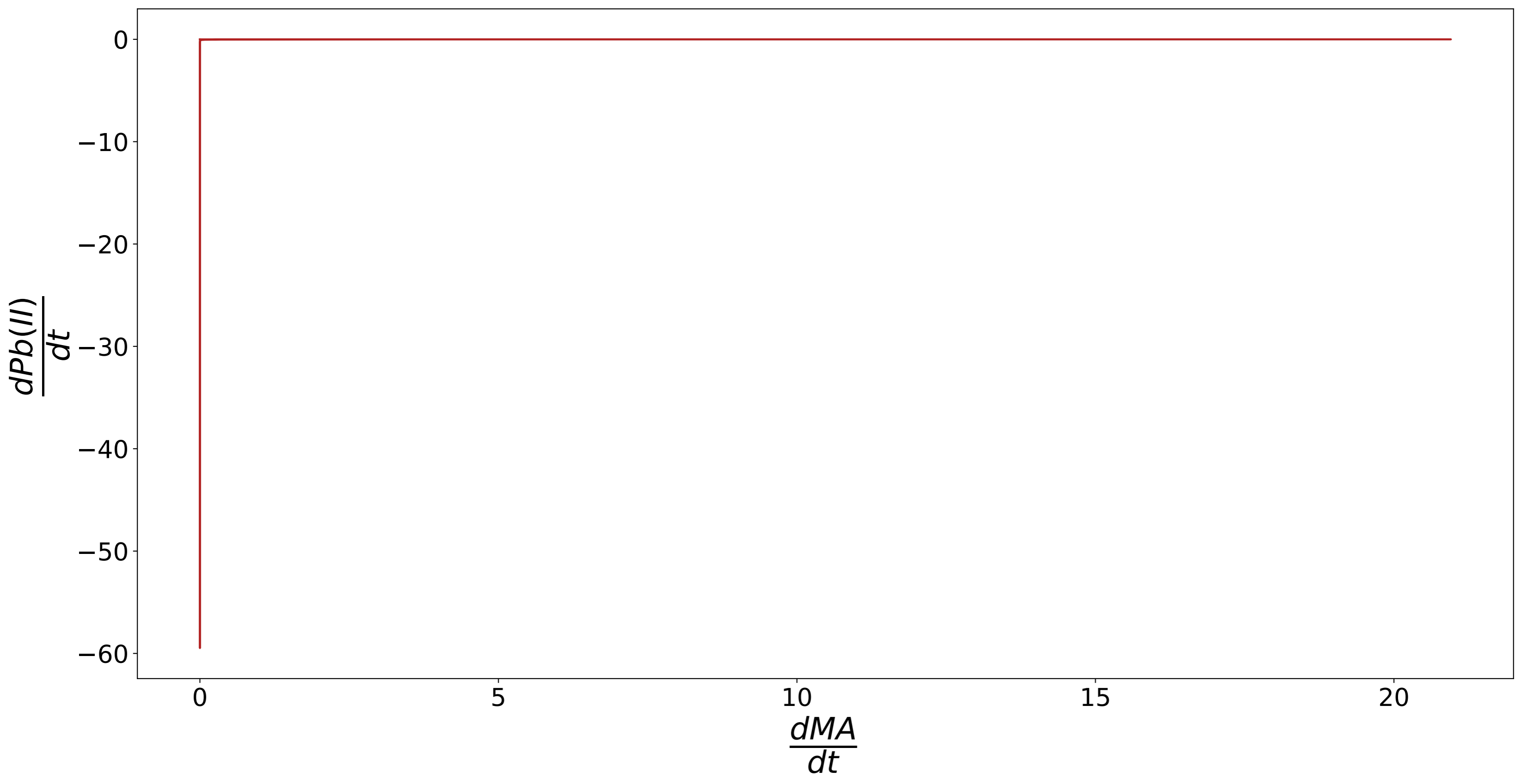

Supplement: Supplementary file 1 [file ijms-23-12255-s001.zip › Definitions/Derivatives_C500_MA_Pb.pdf]

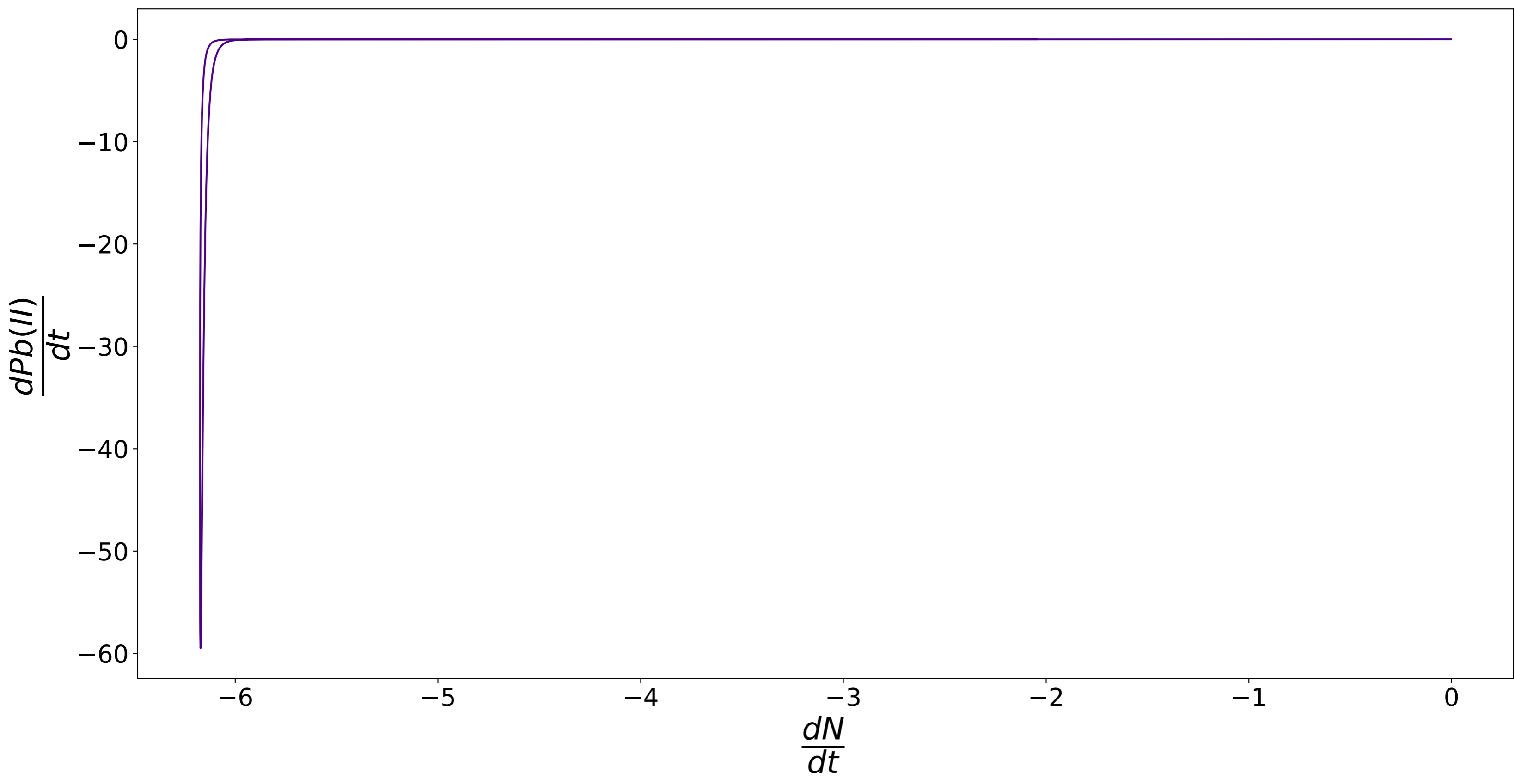

Supplement: Supplementary file 1 [file ijms-23-12255-s001.zip › Definitions/Derivatives_C500_N_Pb.pdf]

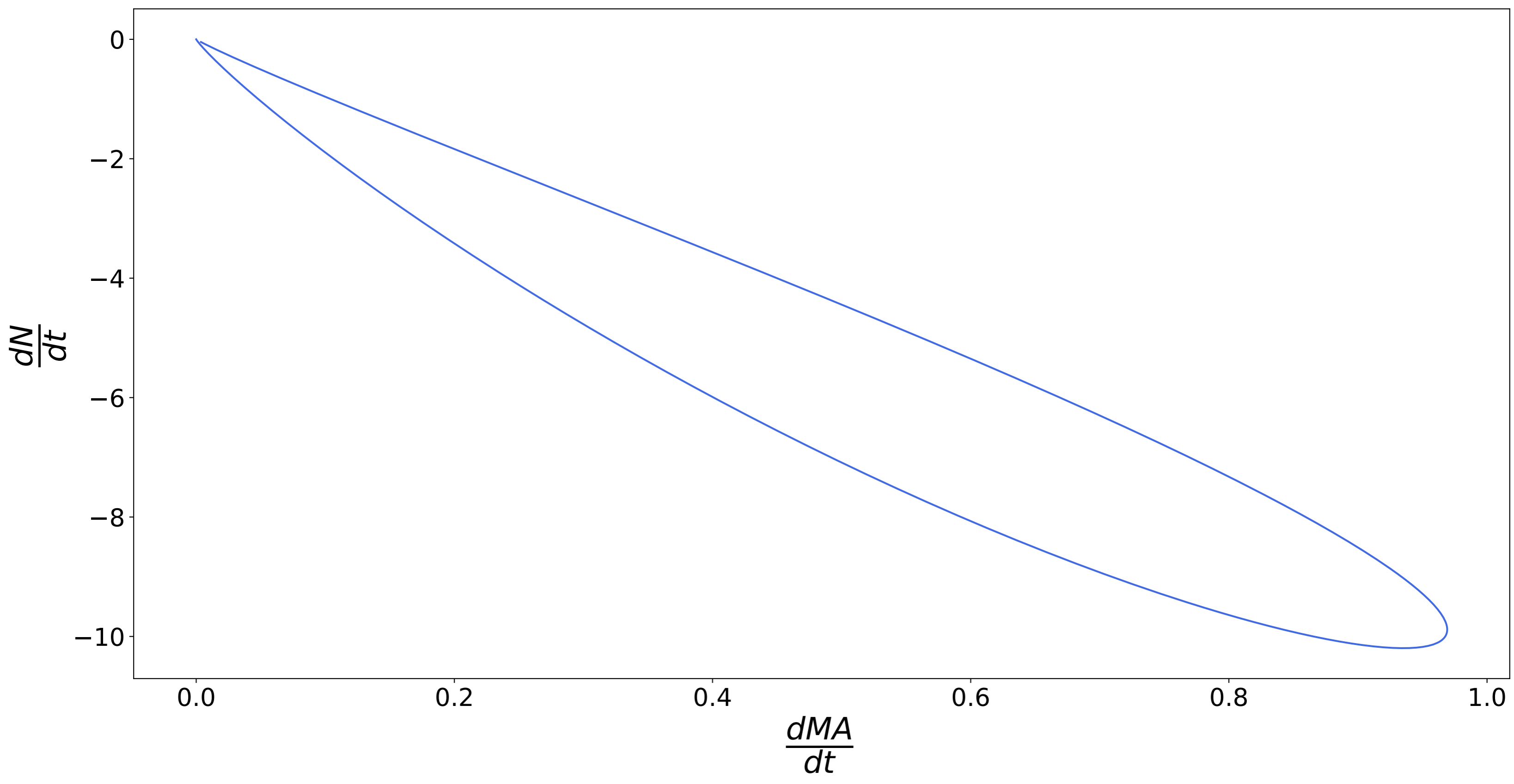

Supplement: Supplementary file 1 [file ijms-23-12255-s001.zip › Definitions/Derivatives_C80_MA_N.pdf]

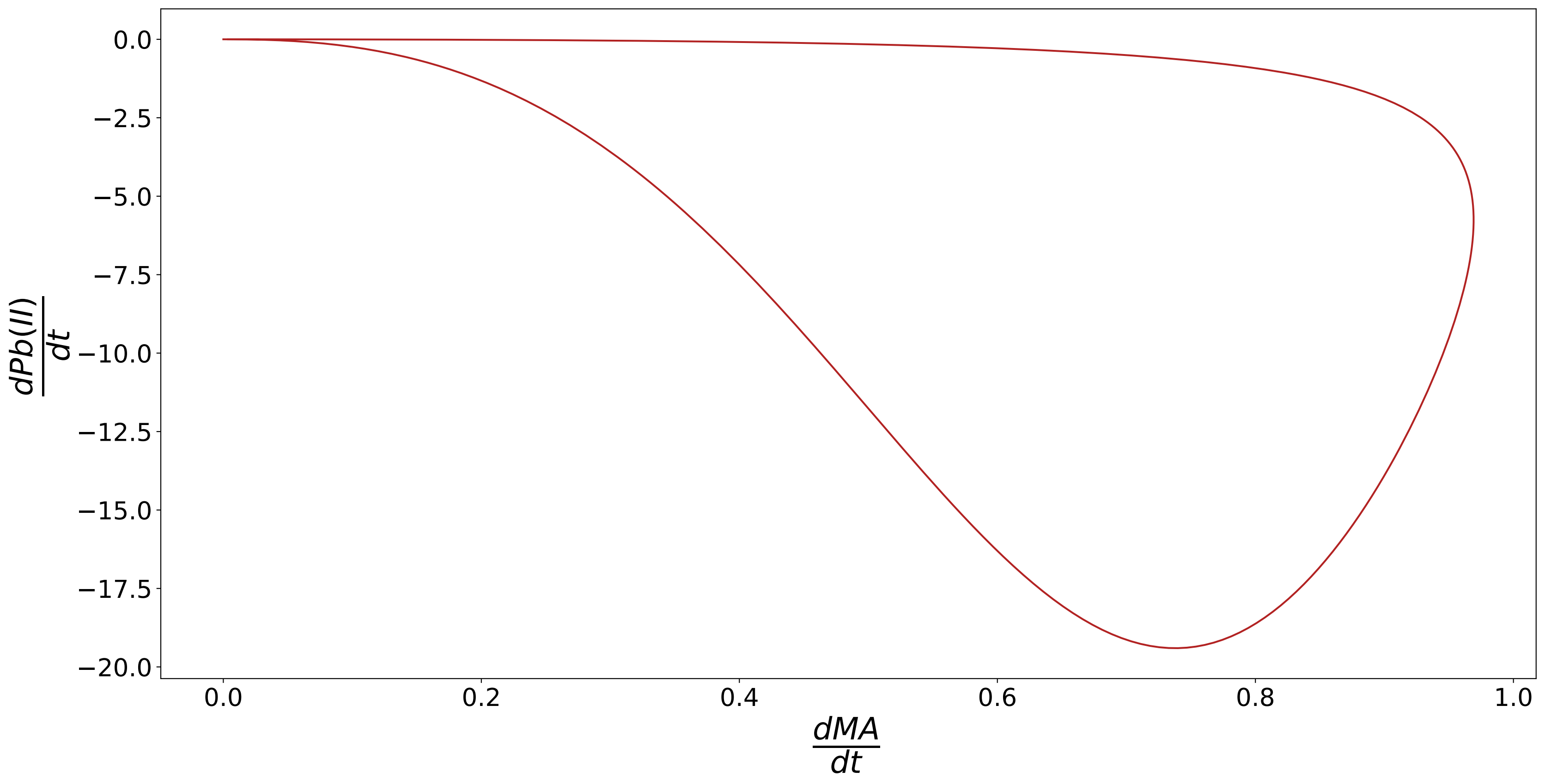

Supplement: Supplementary file 1 [file ijms-23-12255-s001.zip › Definitions/Derivatives_C80_MA_Pb.pdf]

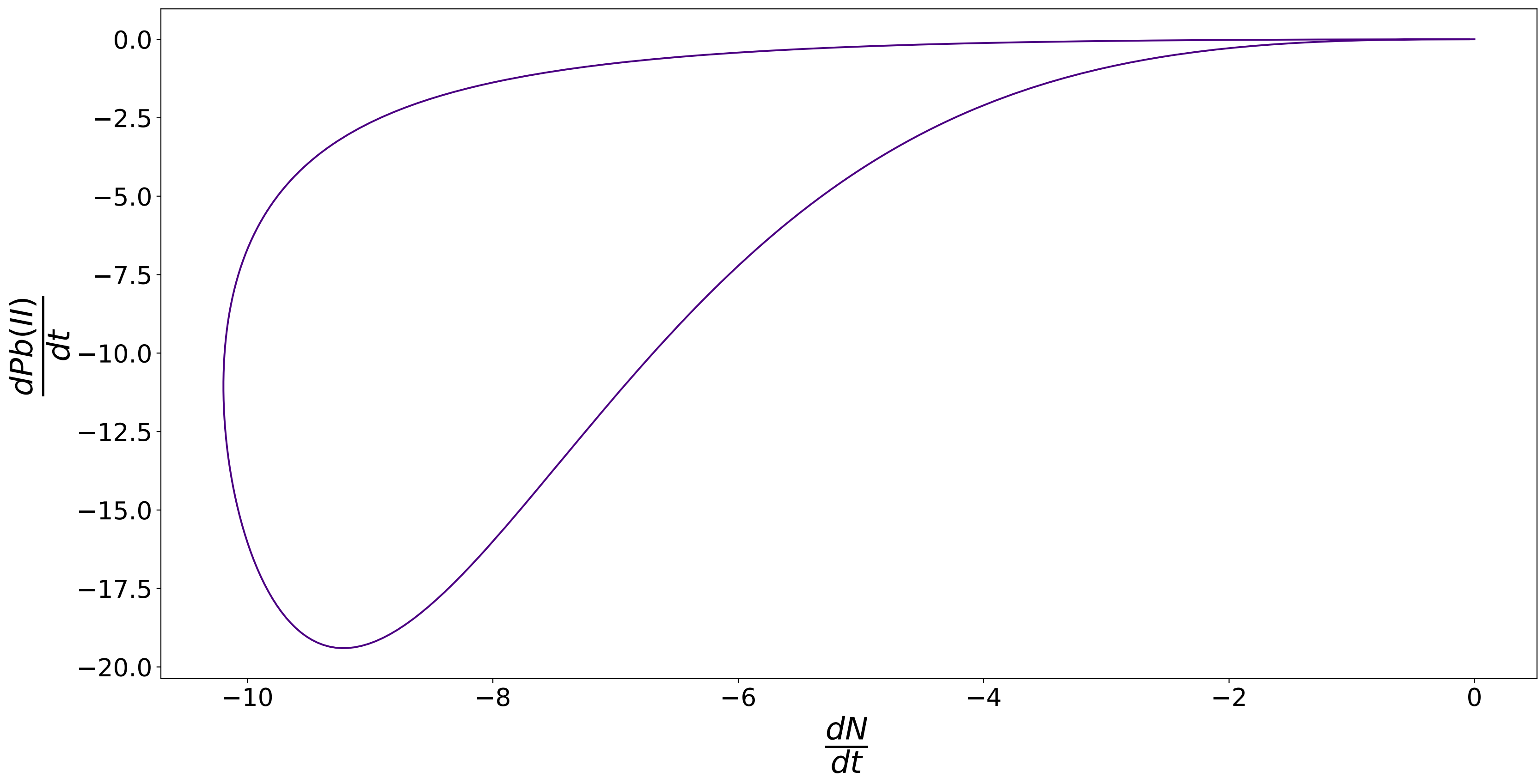

Supplement: Supplementary file 1 [file ijms-23-12255-s001.zip › Definitions/Derivatives_C80_N_Pb.pdf]

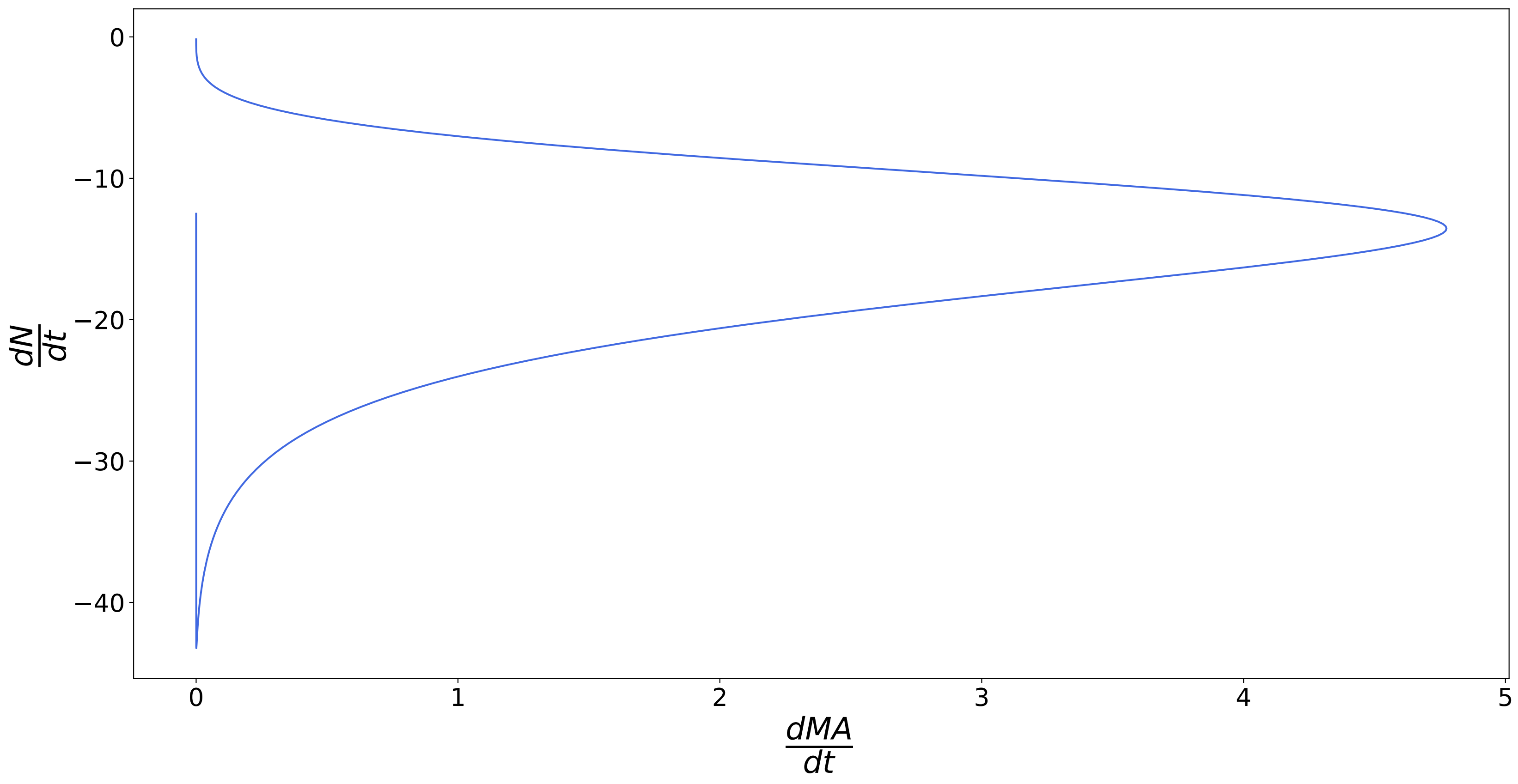

Supplement: Supplementary file 1 [file ijms-23-12255-s001.zip › Definitions/Derivatives_K250_MA_N.pdf]

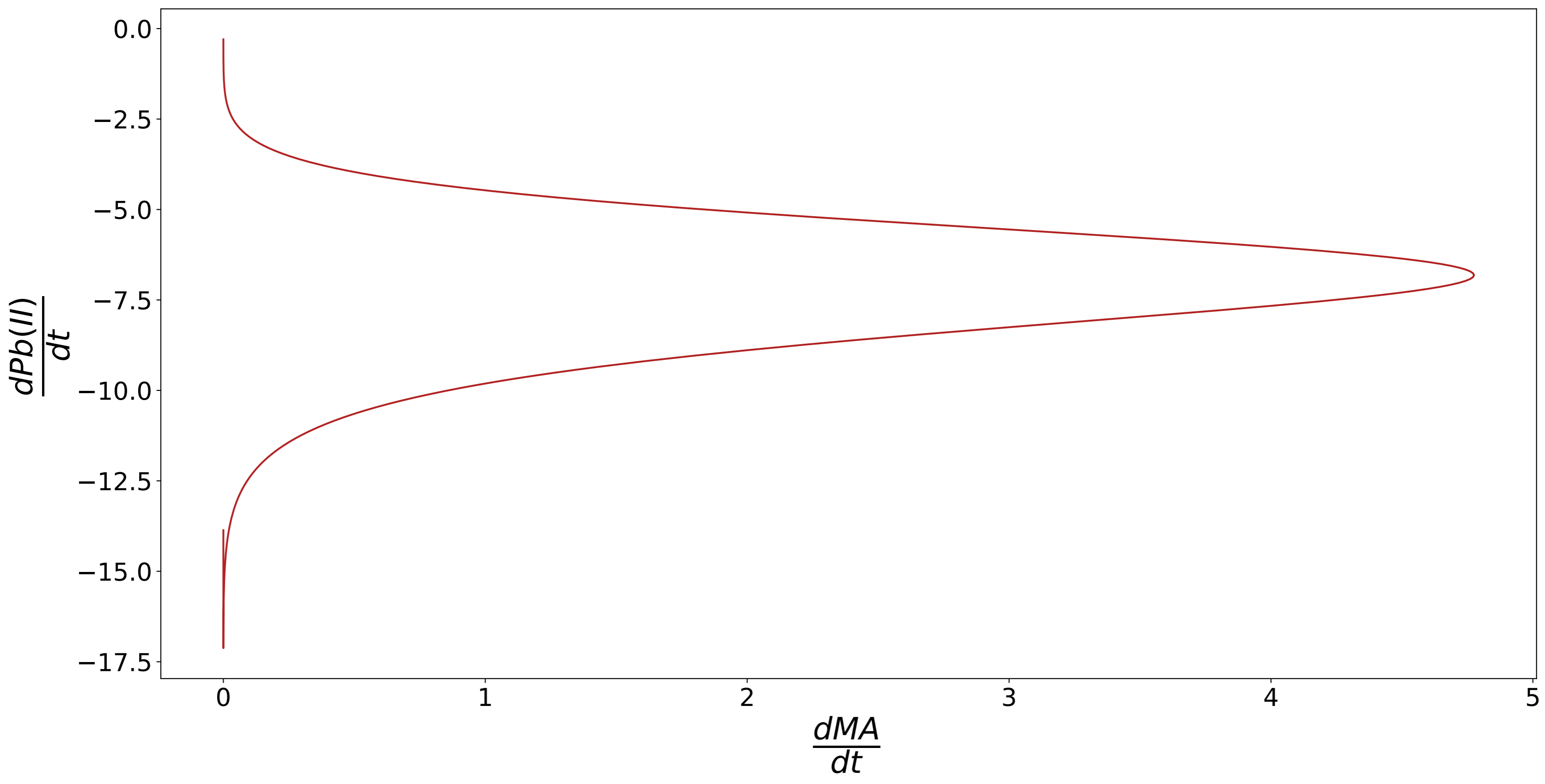

Supplement: Supplementary file 1 [file ijms-23-12255-s001.zip › Definitions/Derivatives_K250_MA_Pb.pdf]

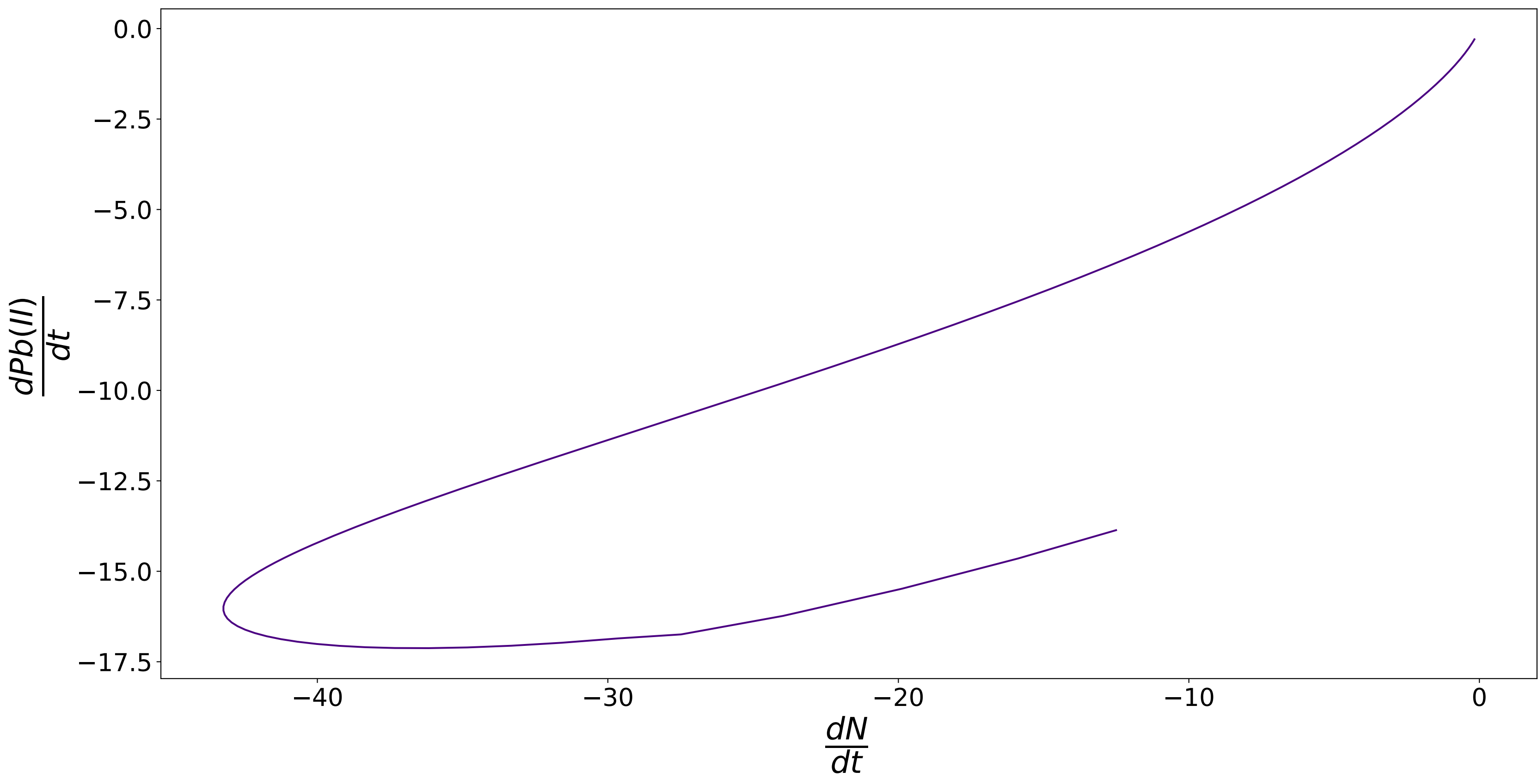

Supplement: Supplementary file 1 [file ijms-23-12255-s001.zip › Definitions/Derivatives_K250_N_Pb.pdf]

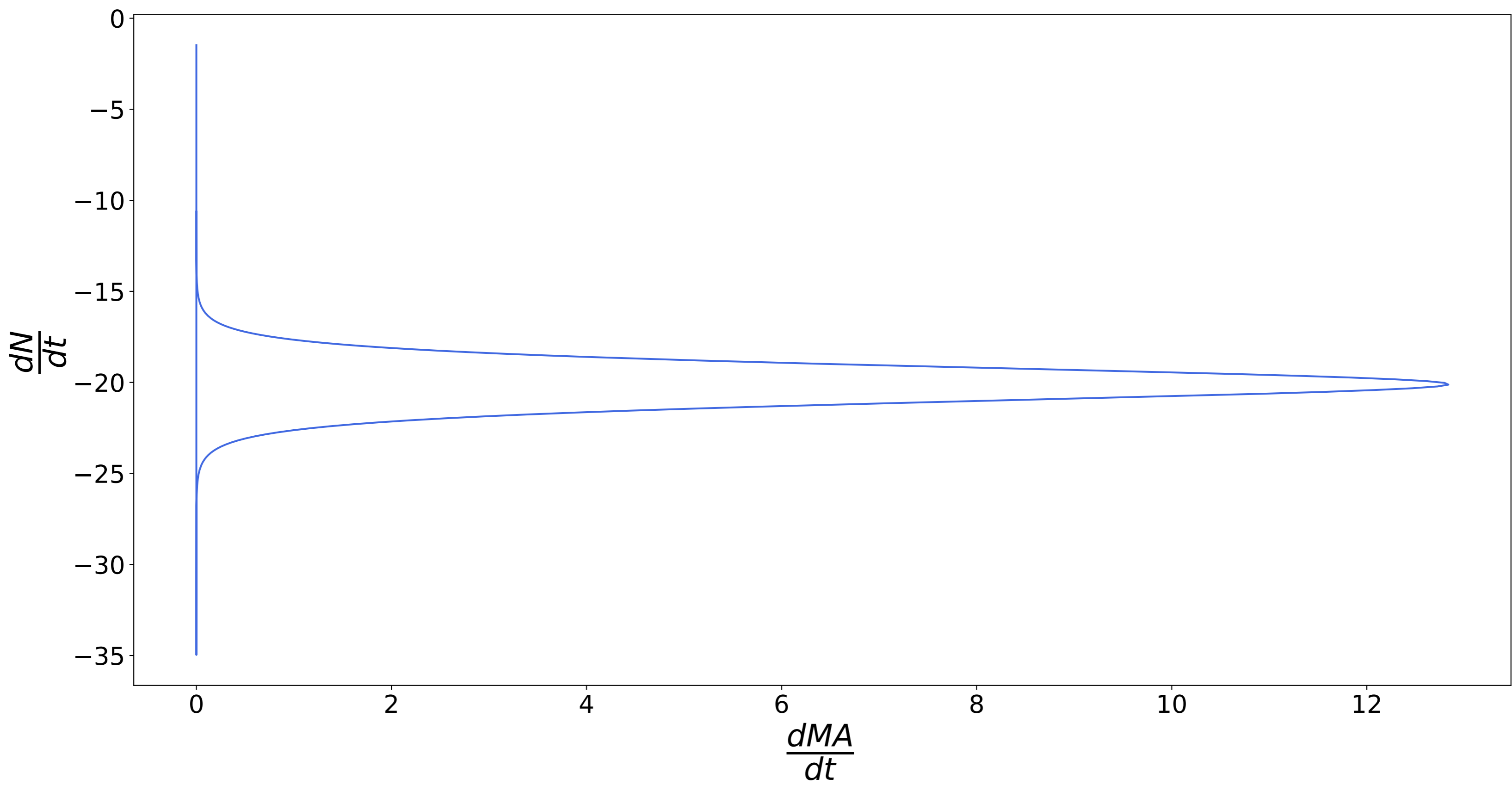

Supplement: Supplementary file 1 [file ijms-23-12255-s001.zip › Definitions/Derivatives_K500_MA_N.pdf]

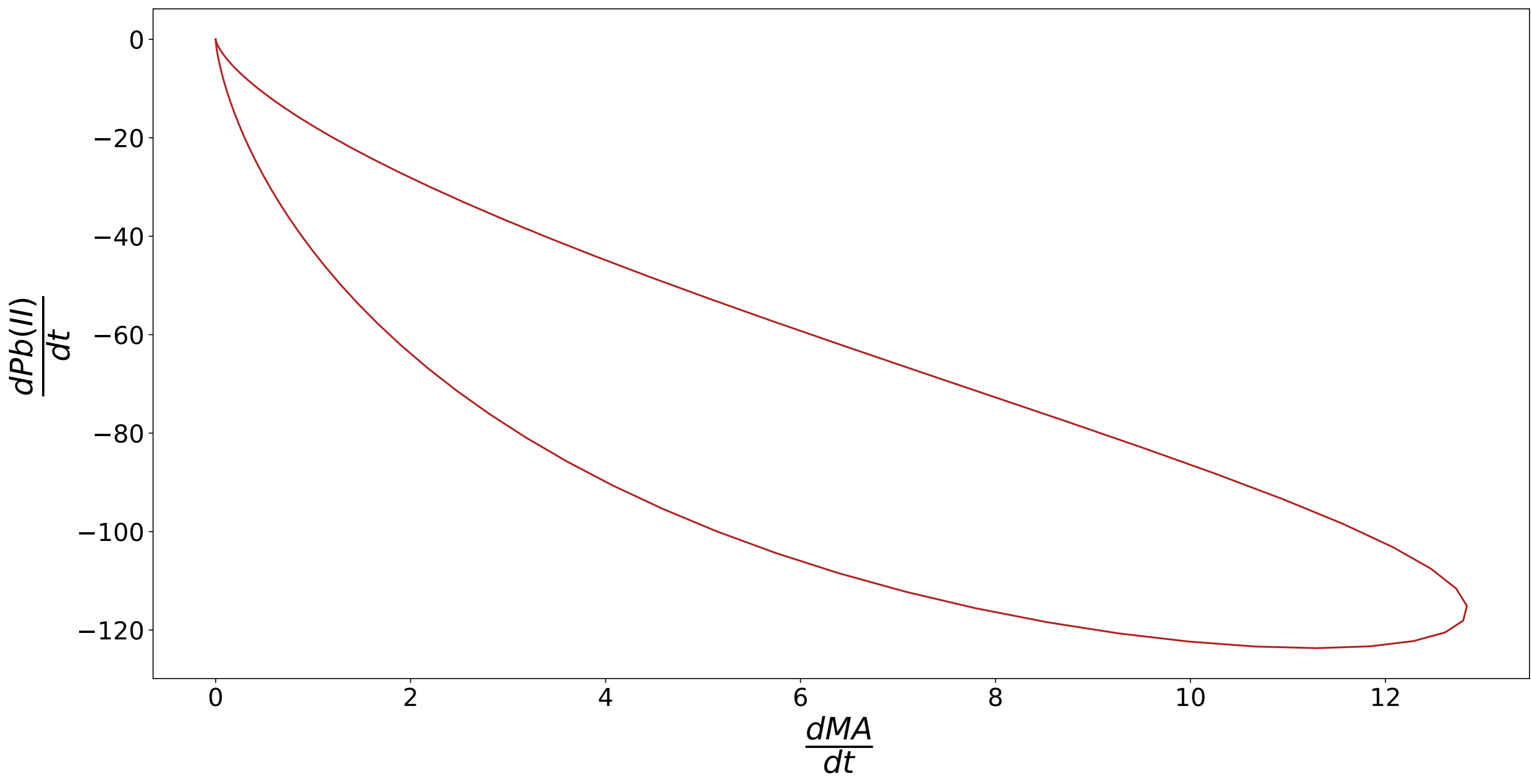

Supplement: Supplementary file 1 [file ijms-23-12255-s001.zip › Definitions/Derivatives_K500_MA_Pb.pdf]

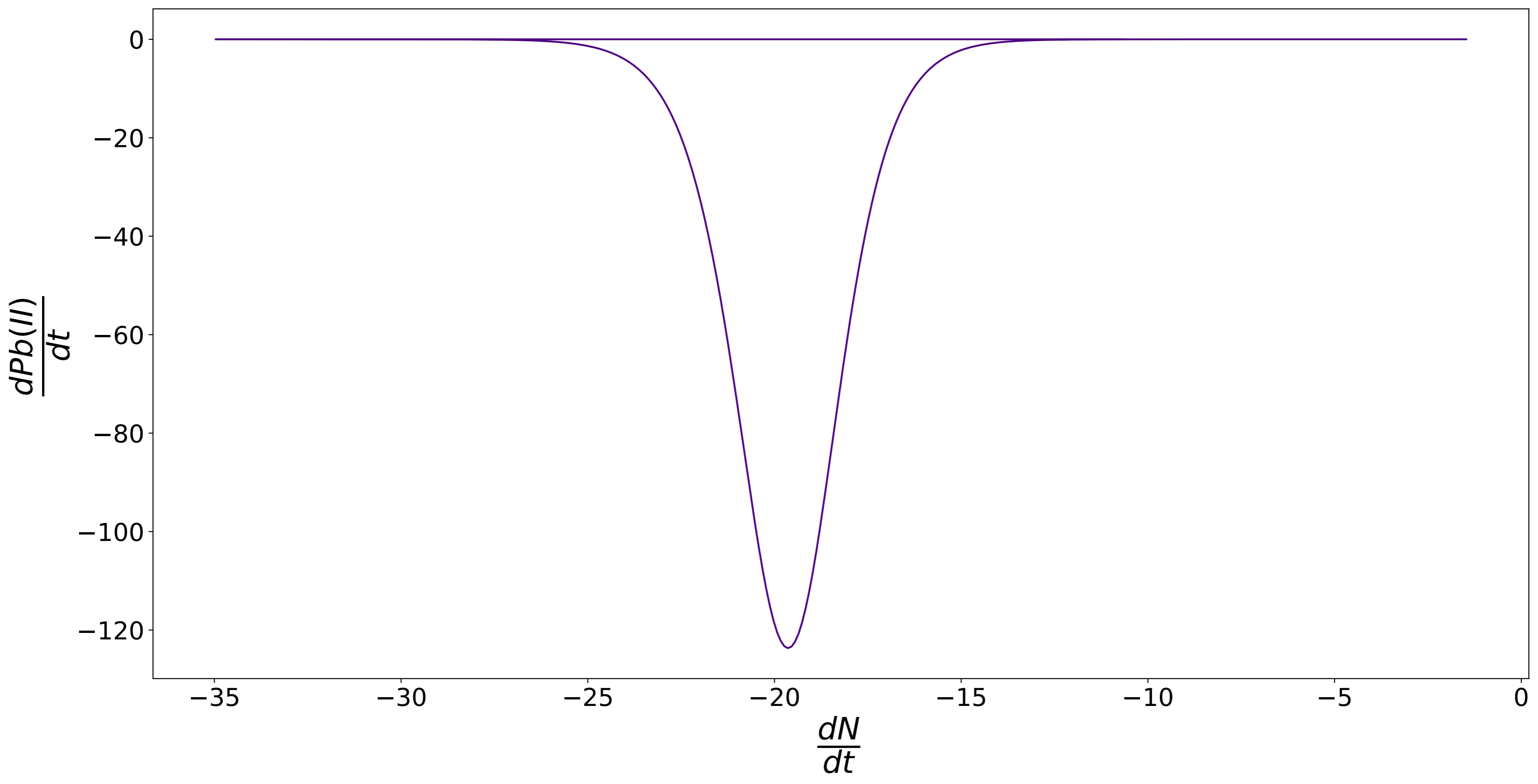

Supplement: Supplementary file 1 [file ijms-23-12255-s001.zip › Definitions/Derivatives_K500_N_Pb.pdf]

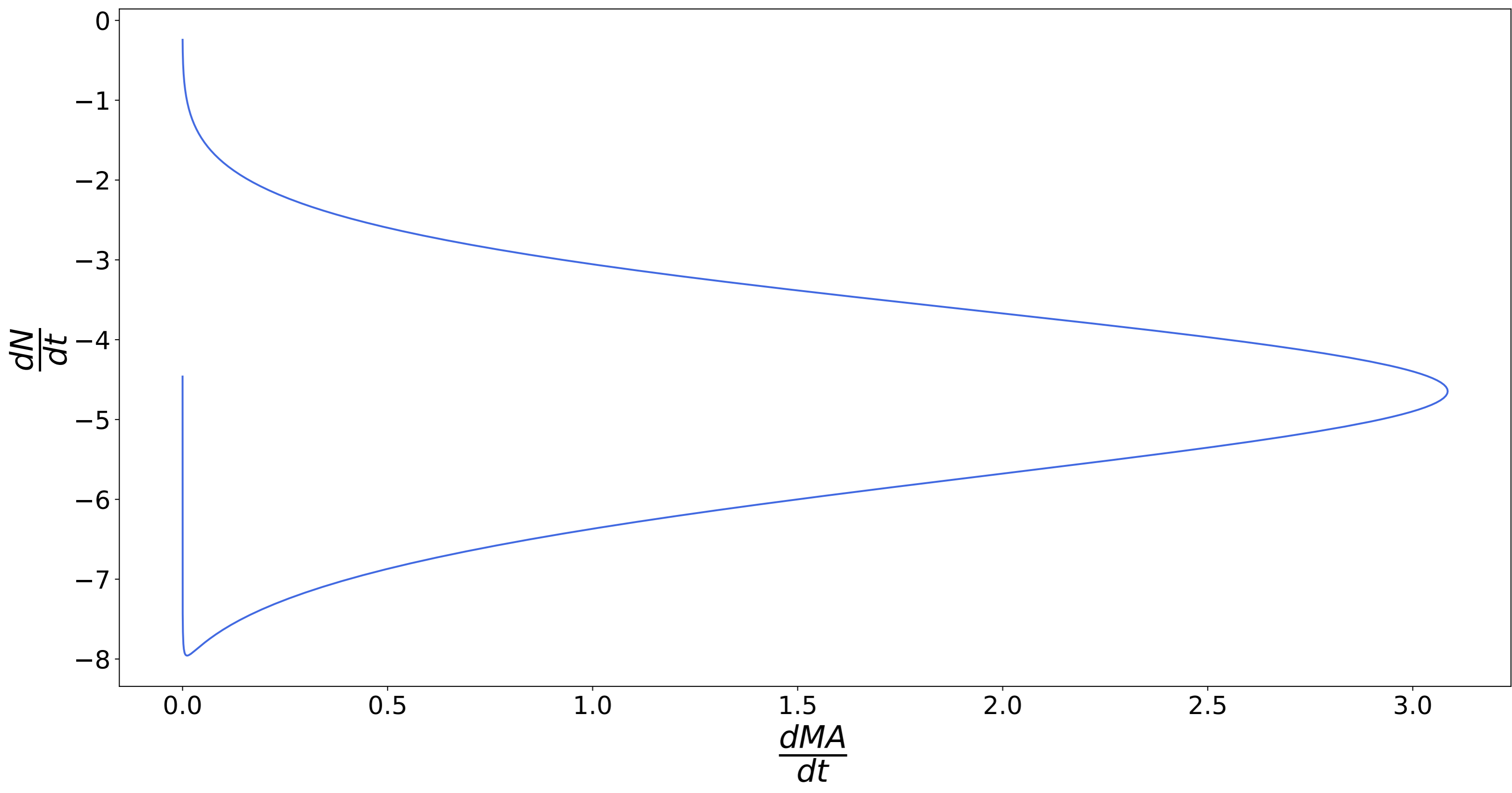

Supplement: Supplementary file 1 [file ijms-23-12255-s001.zip › Definitions/Derivatives_K80_MA_N.pdf]

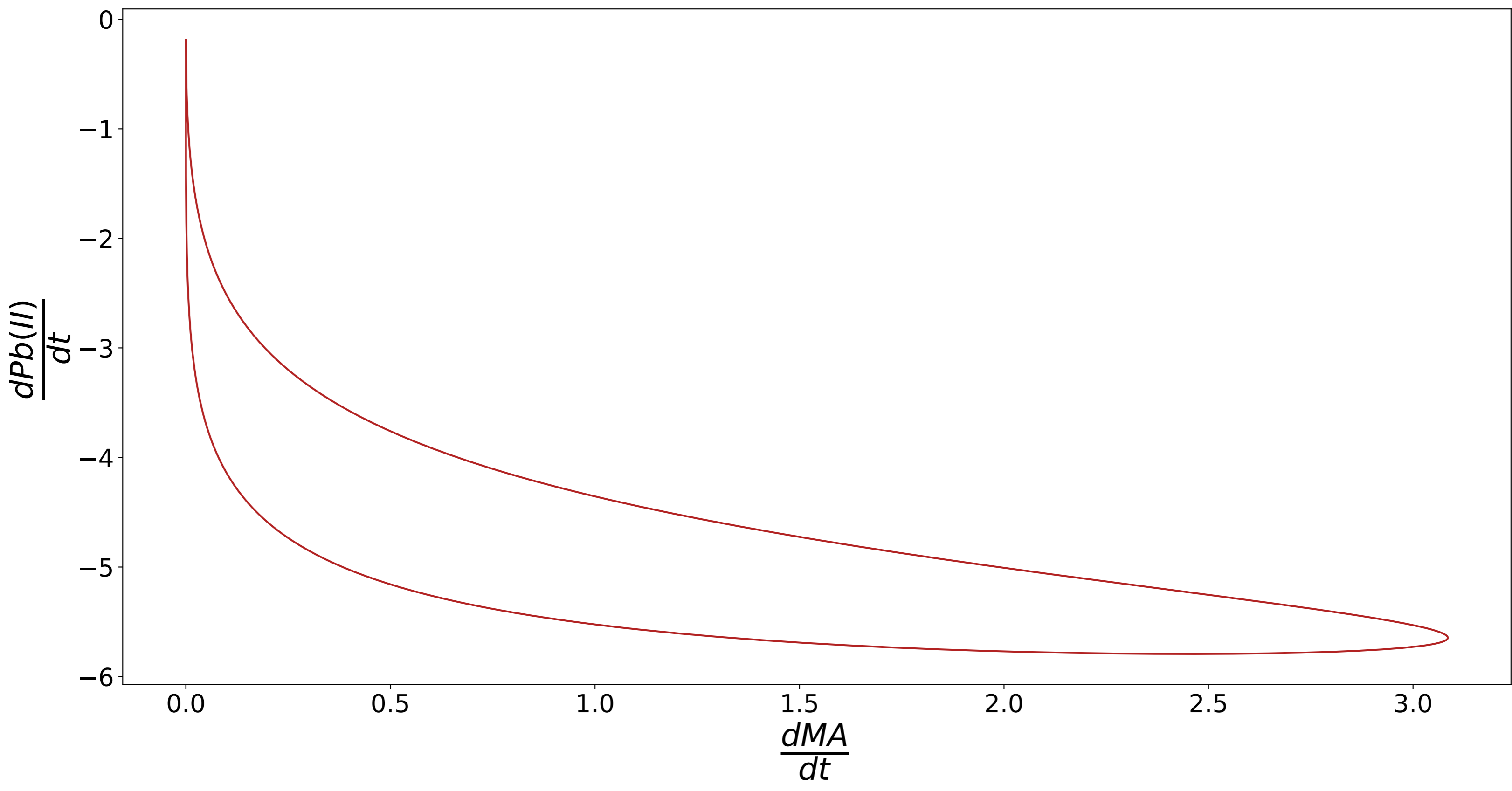

Supplement: Supplementary file 1 [file ijms-23-12255-s001.zip › Definitions/Derivatives_K80_MA_Pb.pdf]

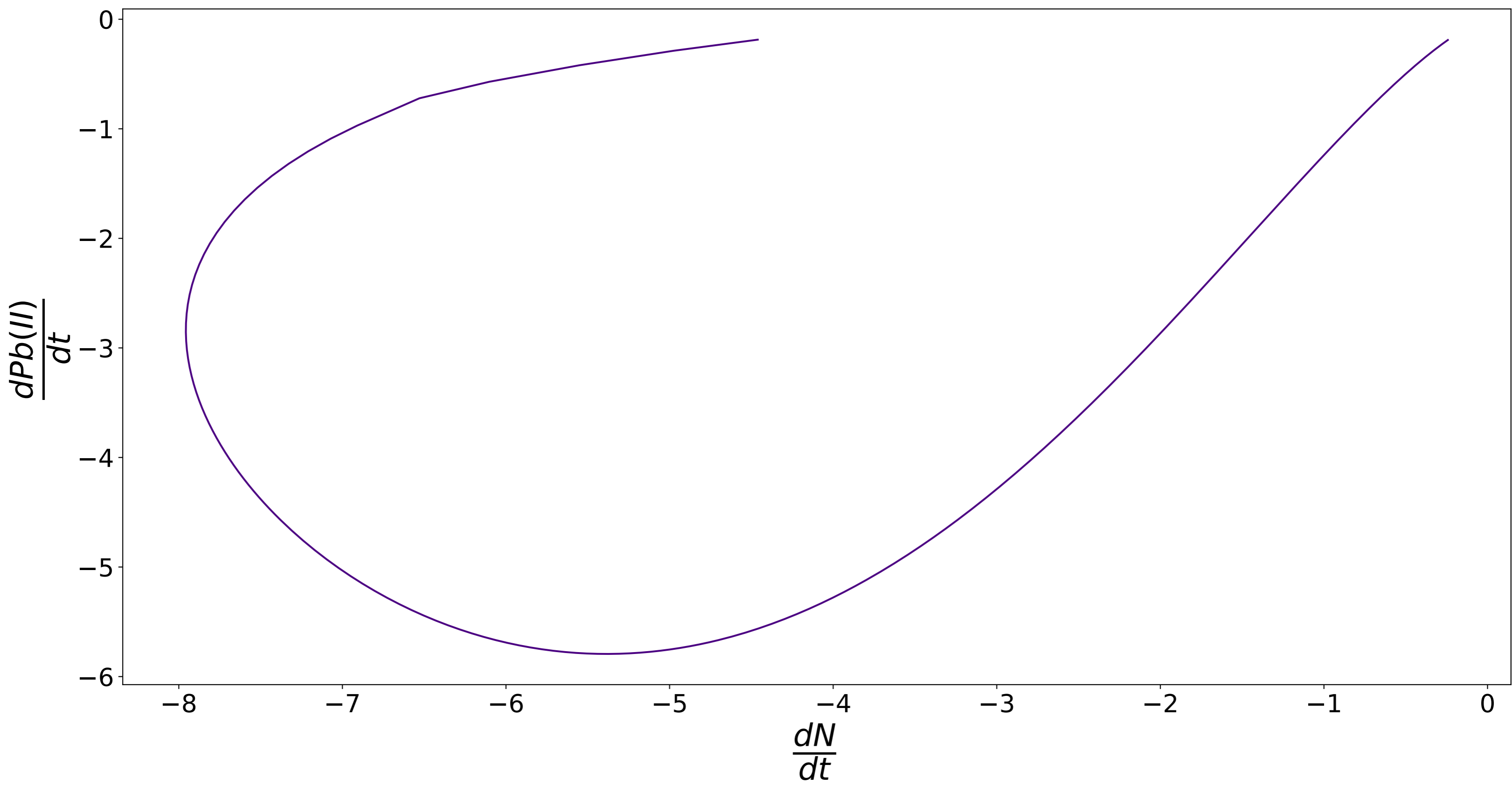

Supplement: Supplementary file 1 [file ijms-23-12255-s001.zip › Definitions/Derivatives_K80_N_Pb.pdf]

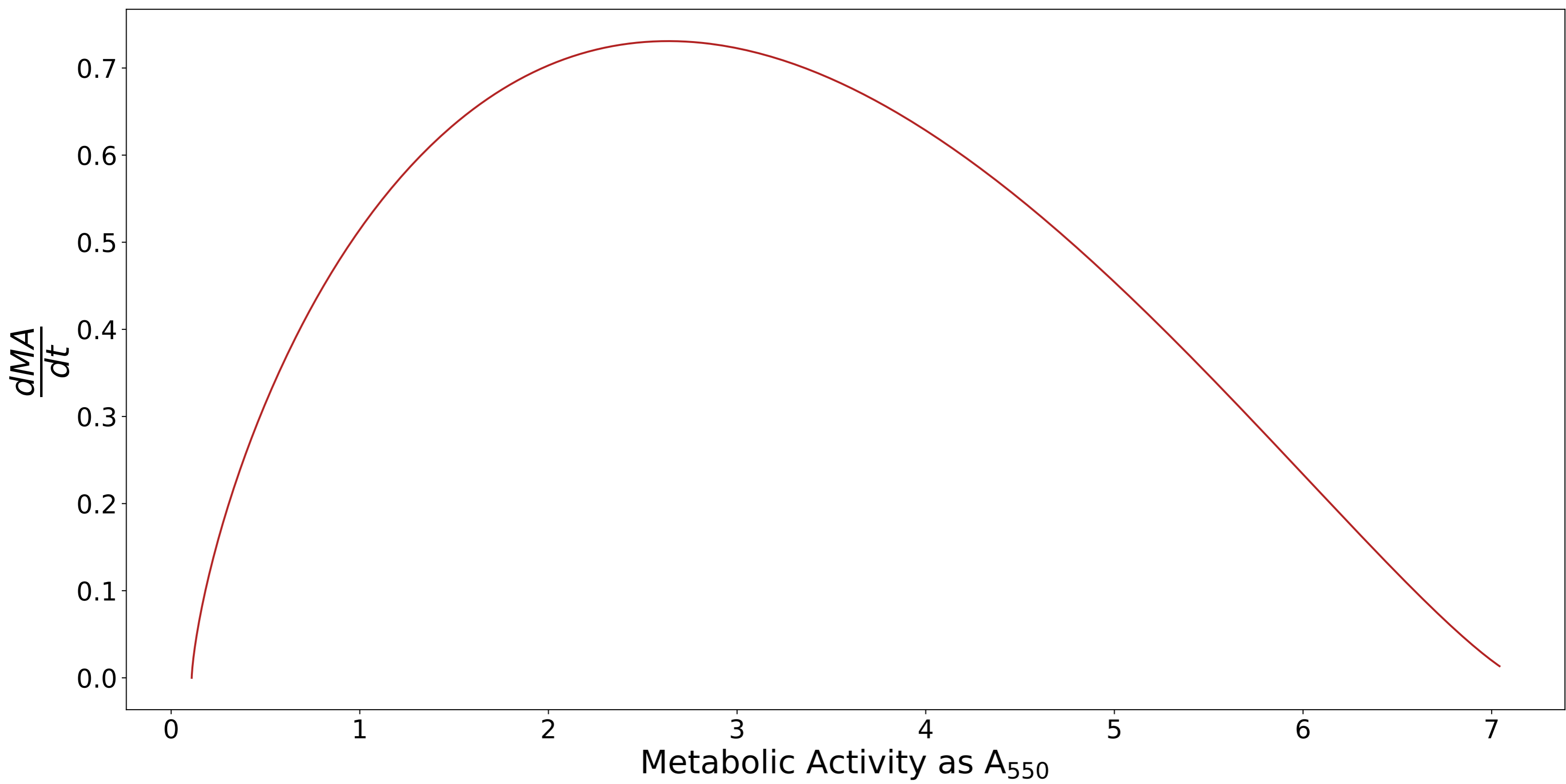

Supplement: Supplementary file 1 [file ijms-23-12255-s001.zip › Definitions/dMA_MA_C250.pdf]

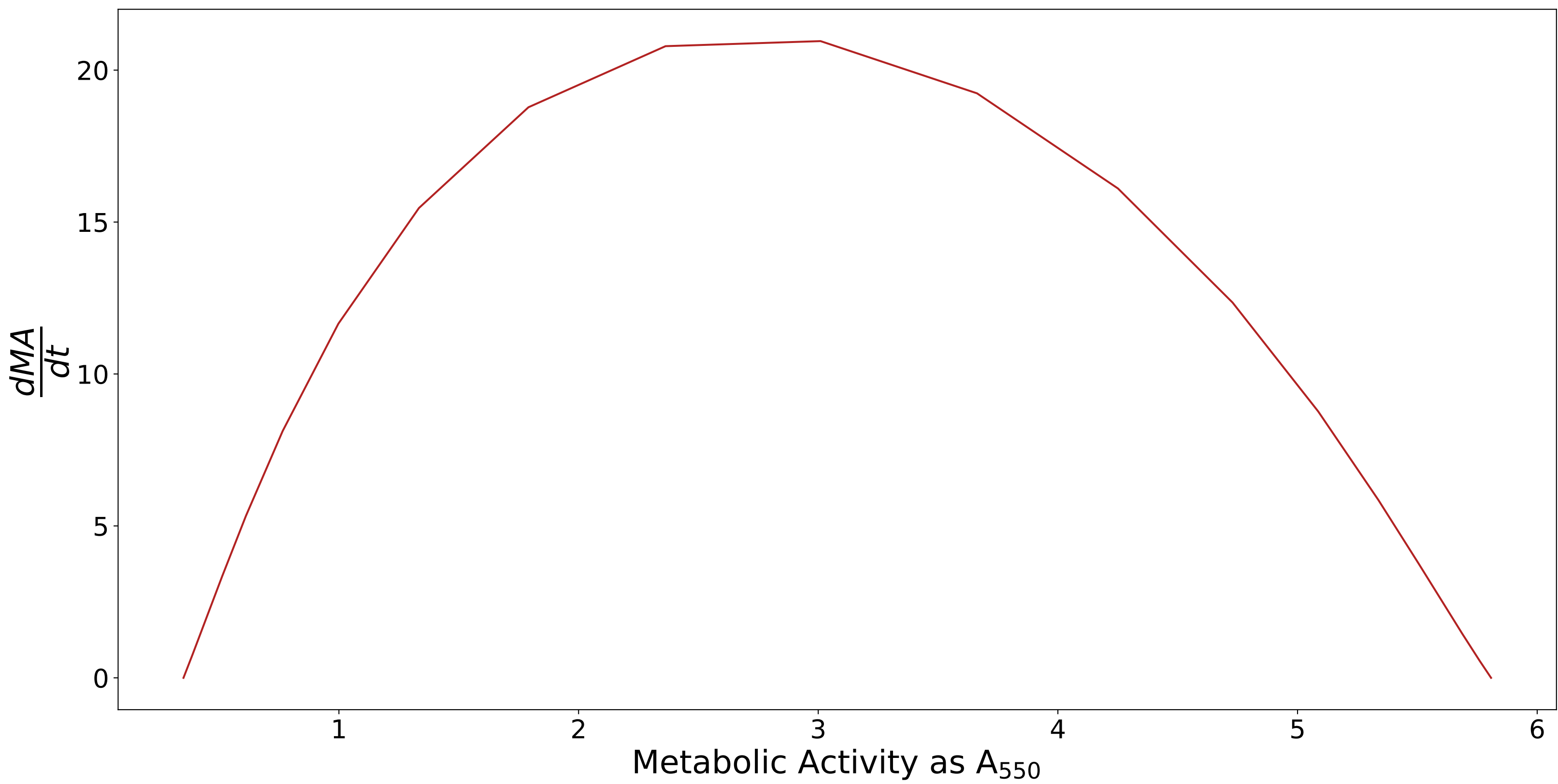

Supplement: Supplementary file 1 [file ijms-23-12255-s001.zip › Definitions/dMA_MA_C500.pdf]

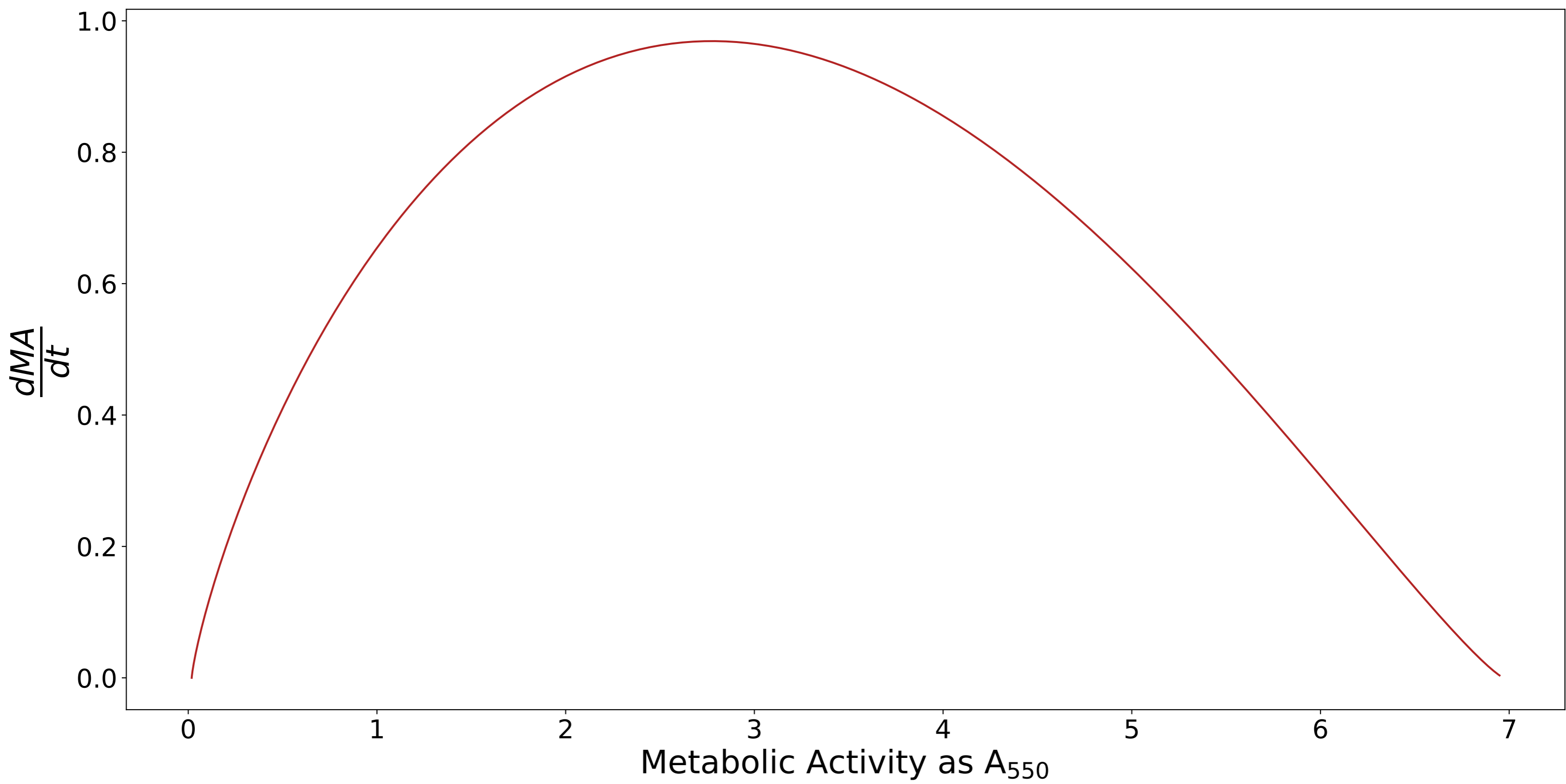

Supplement: Supplementary file 1 [file ijms-23-12255-s001.zip › Definitions/dMA_MA_C80.pdf]

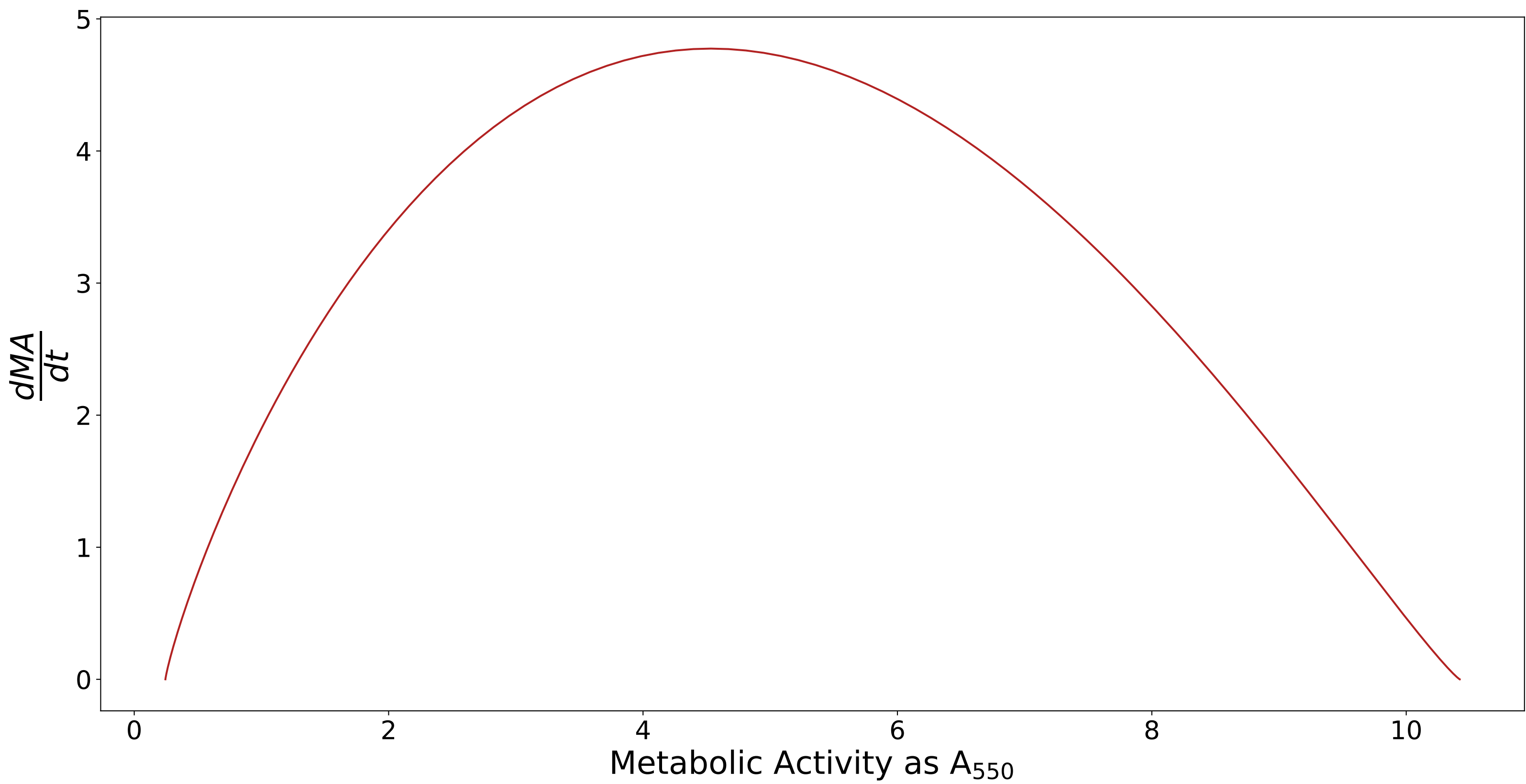

Supplement: Supplementary file 1 [file ijms-23-12255-s001.zip › Definitions/dMA_MA_K250.pdf]

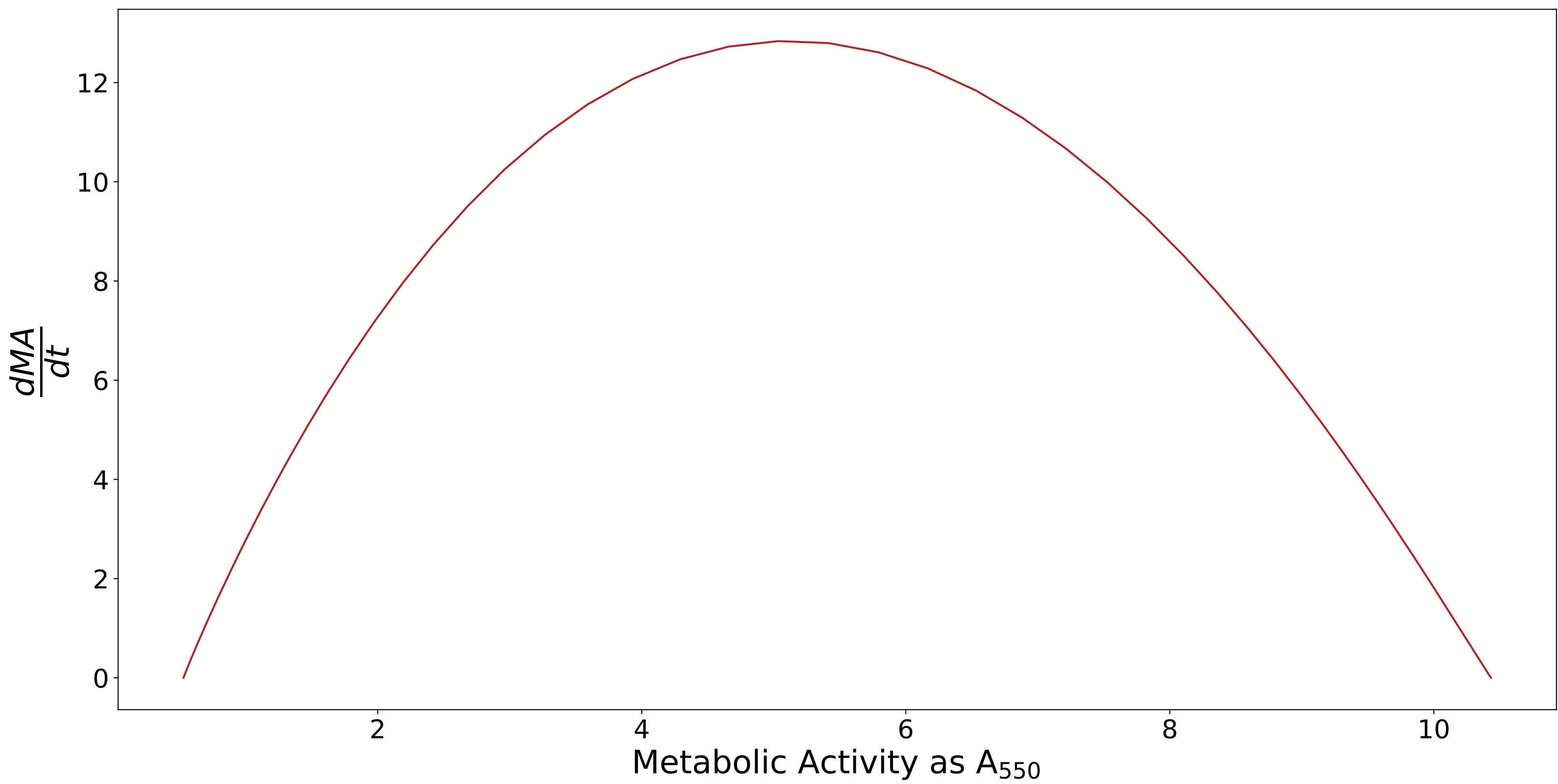

Supplement: Supplementary file 1 [file ijms-23-12255-s001.zip › Definitions/dMA_MA_K500.pdf]

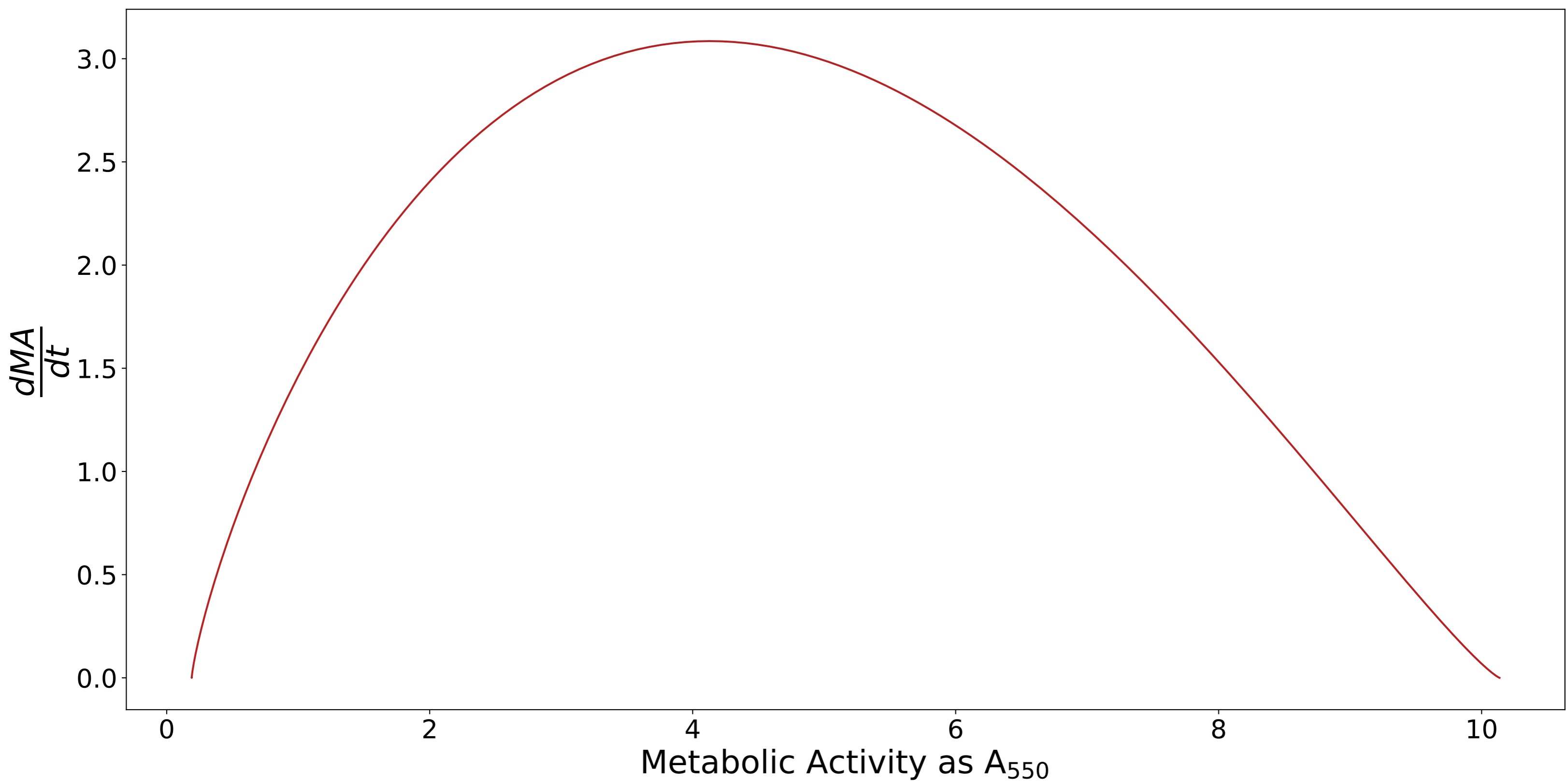

Supplement: Supplementary file 1 [file ijms-23-12255-s001.zip › Definitions/dMA_MA_K80.pdf]

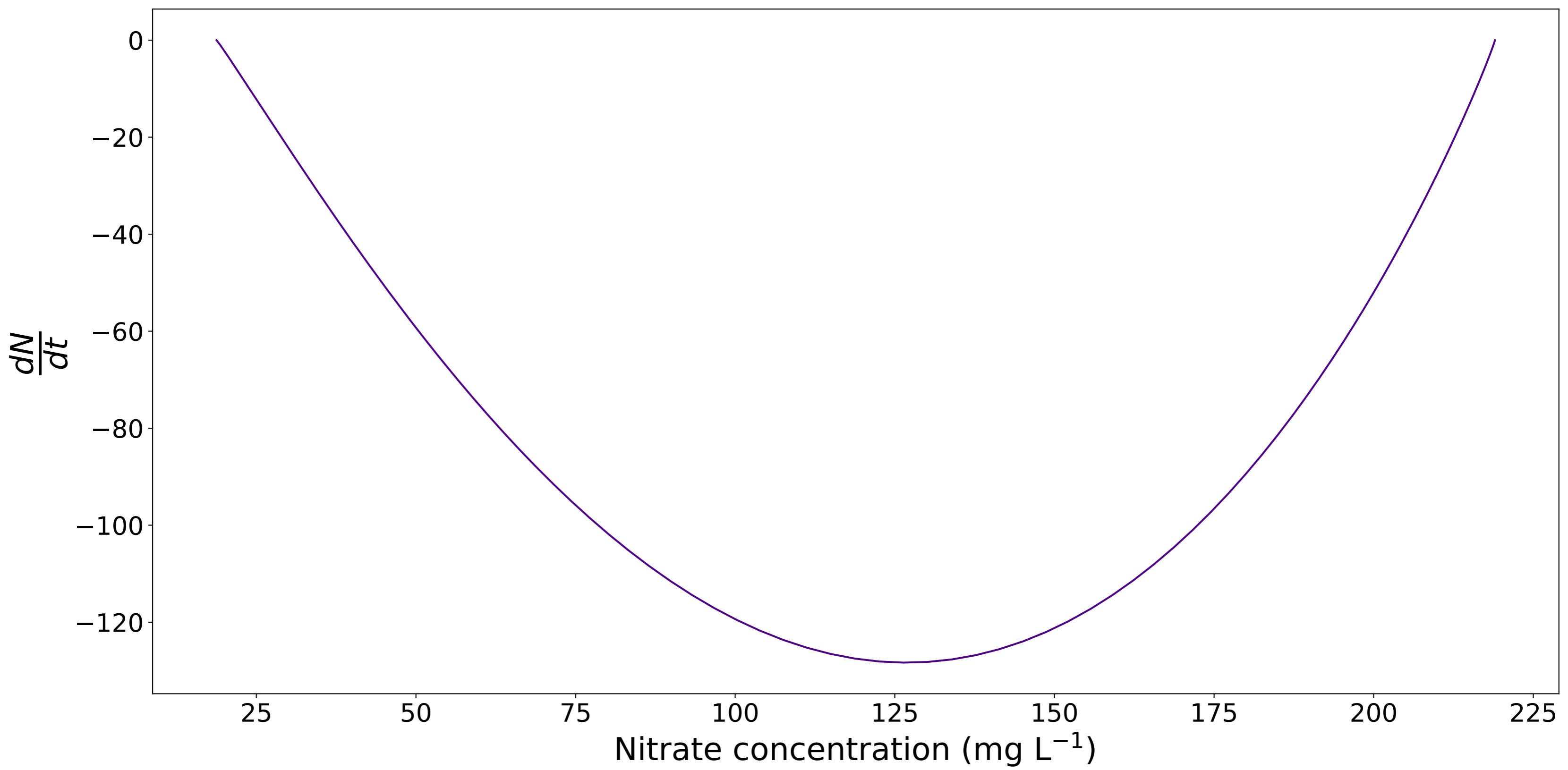

Supplement: Supplementary file 1 [file ijms-23-12255-s001.zip › Definitions/dN_N_C250.pdf]

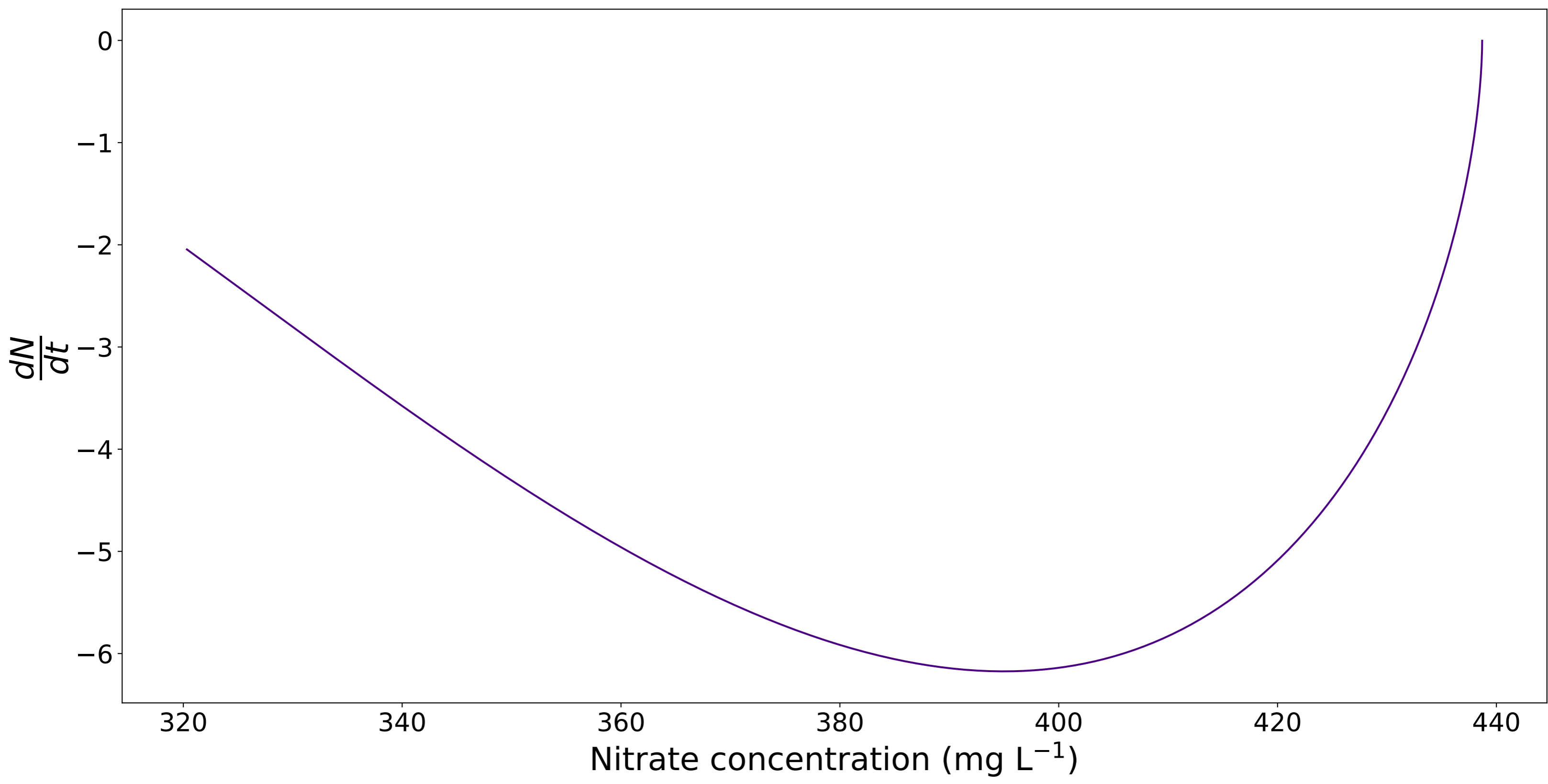

Supplement: Supplementary file 1 [file ijms-23-12255-s001.zip › Definitions/dN_N_C500.pdf]

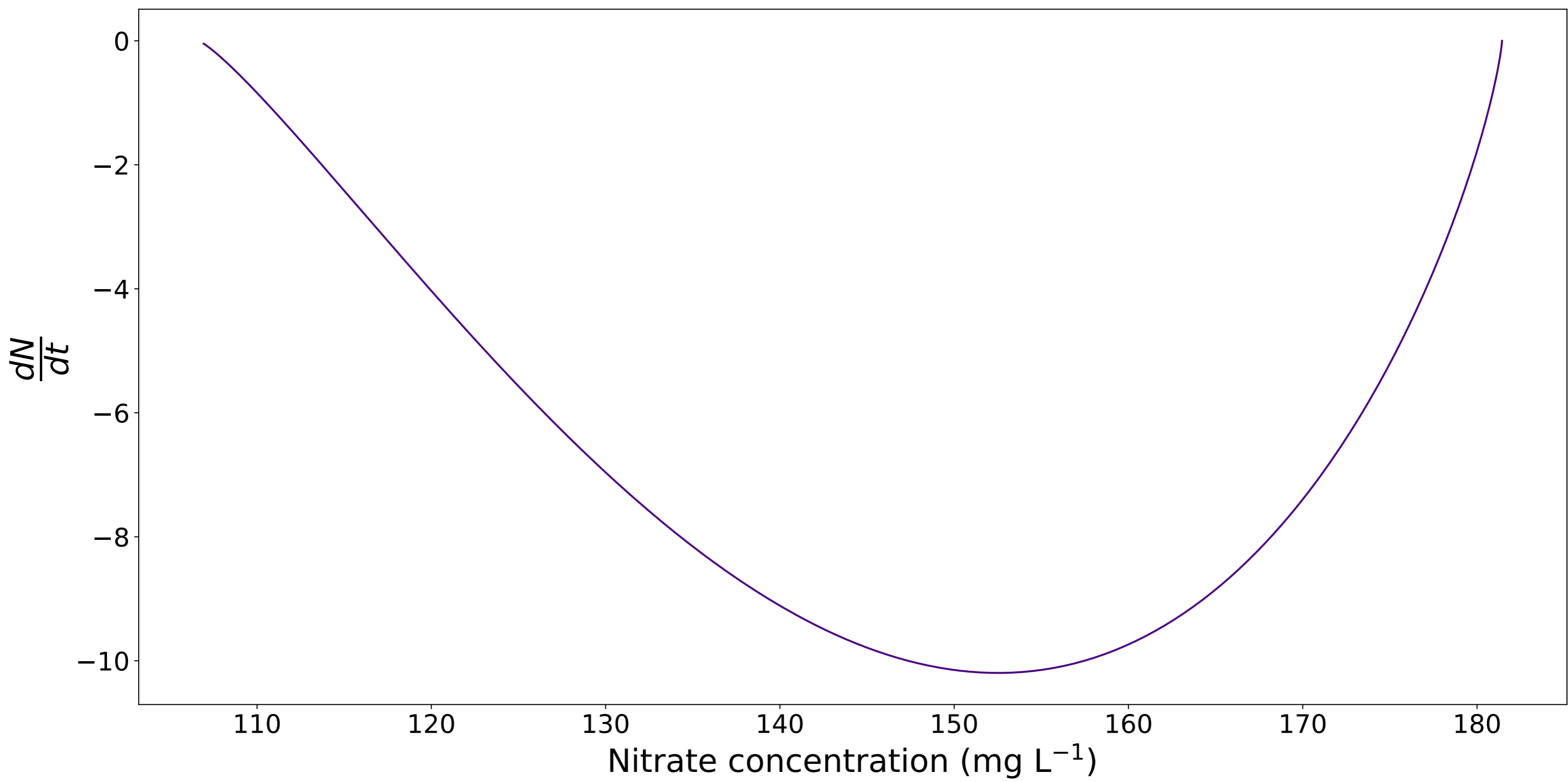

Supplement: Supplementary file 1 [file ijms-23-12255-s001.zip › Definitions/dN_N_C80.pdf]

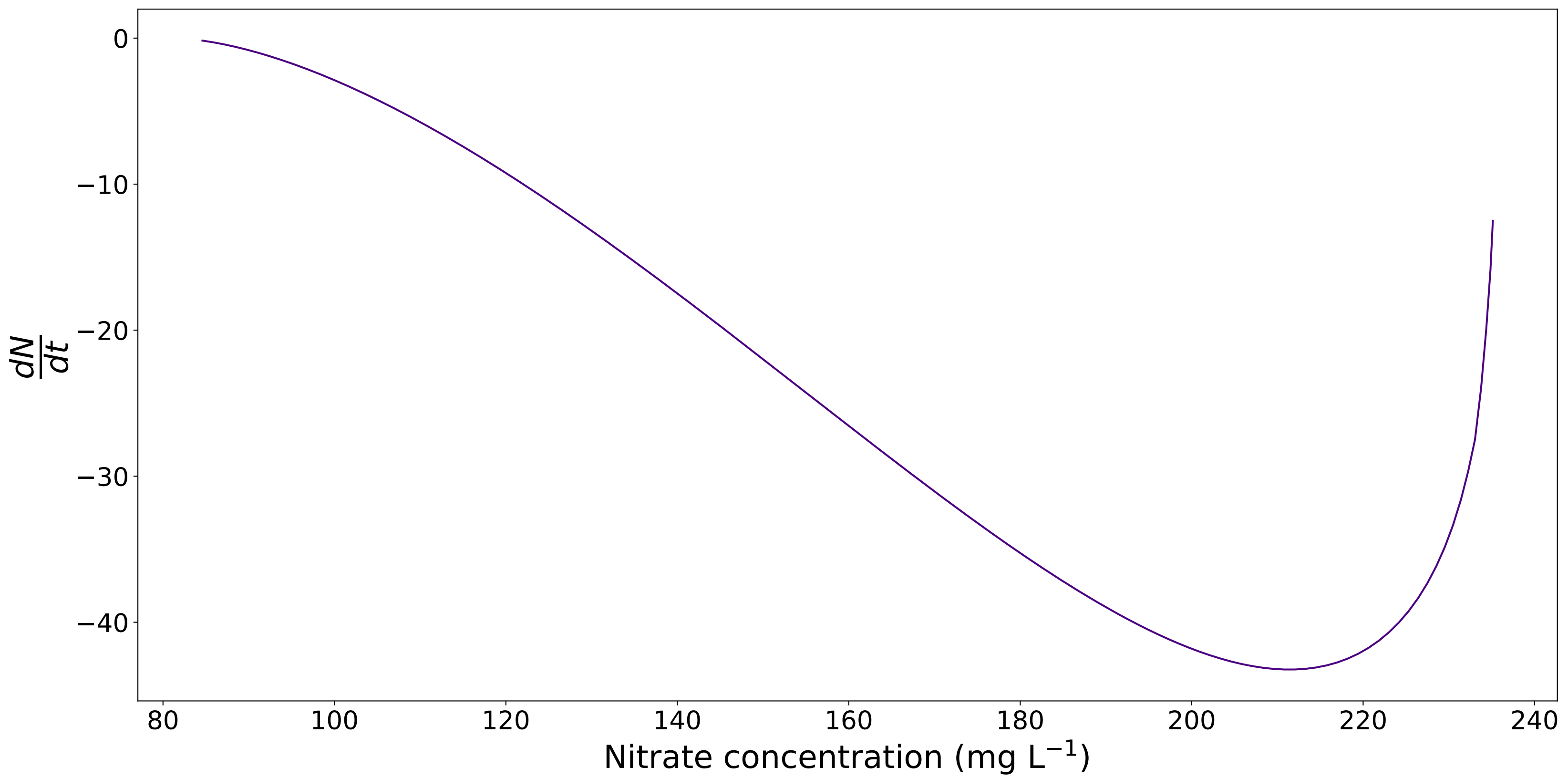

Supplement: Supplementary file 1 [file ijms-23-12255-s001.zip › Definitions/dN_N_K250.pdf]

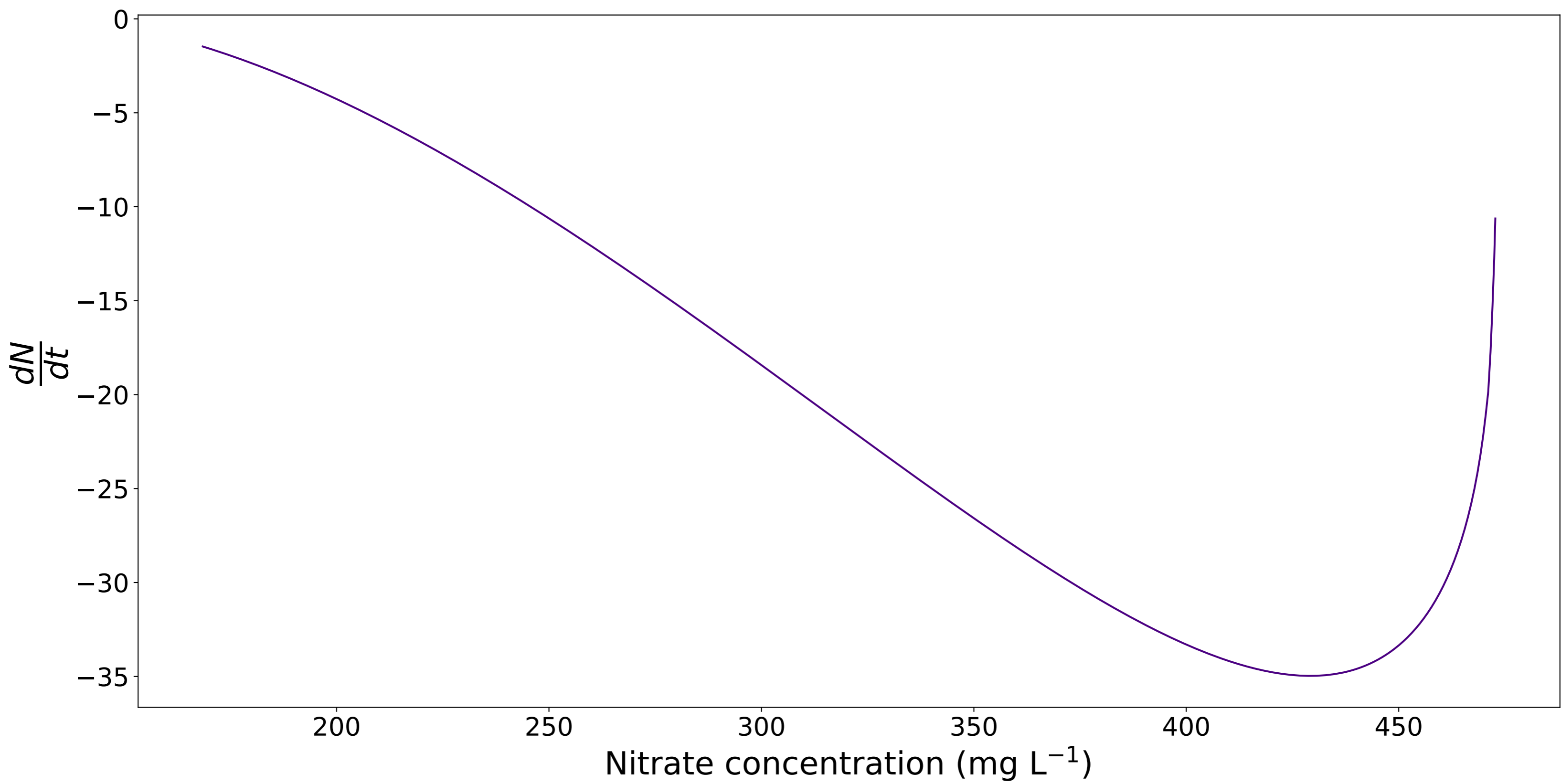

Supplement: Supplementary file 1 [file ijms-23-12255-s001.zip › Definitions/dN_N_K500.pdf]

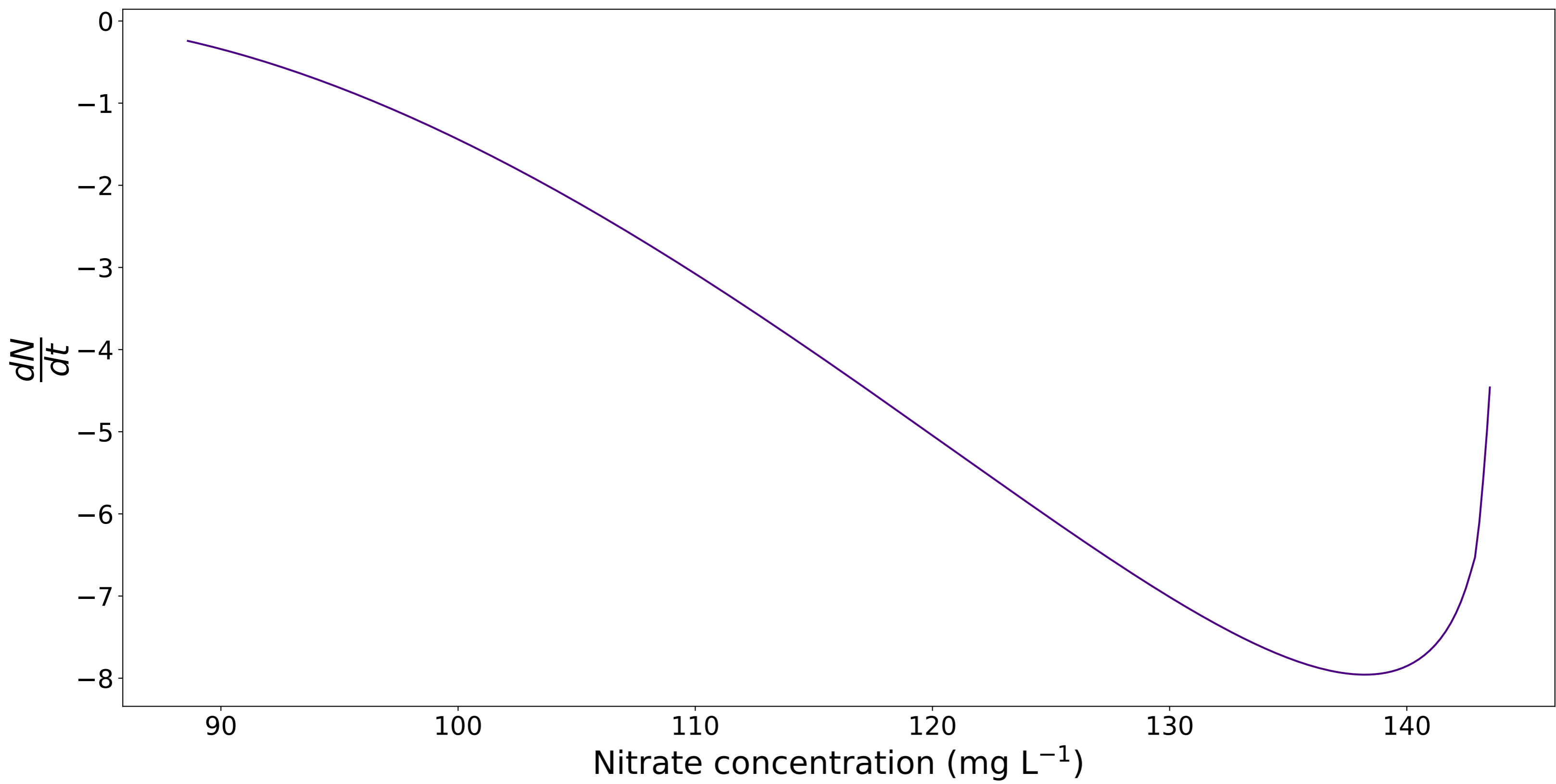

Supplement: Supplementary file 1 [file ijms-23-12255-s001.zip › Definitions/dN_N_K80.pdf]

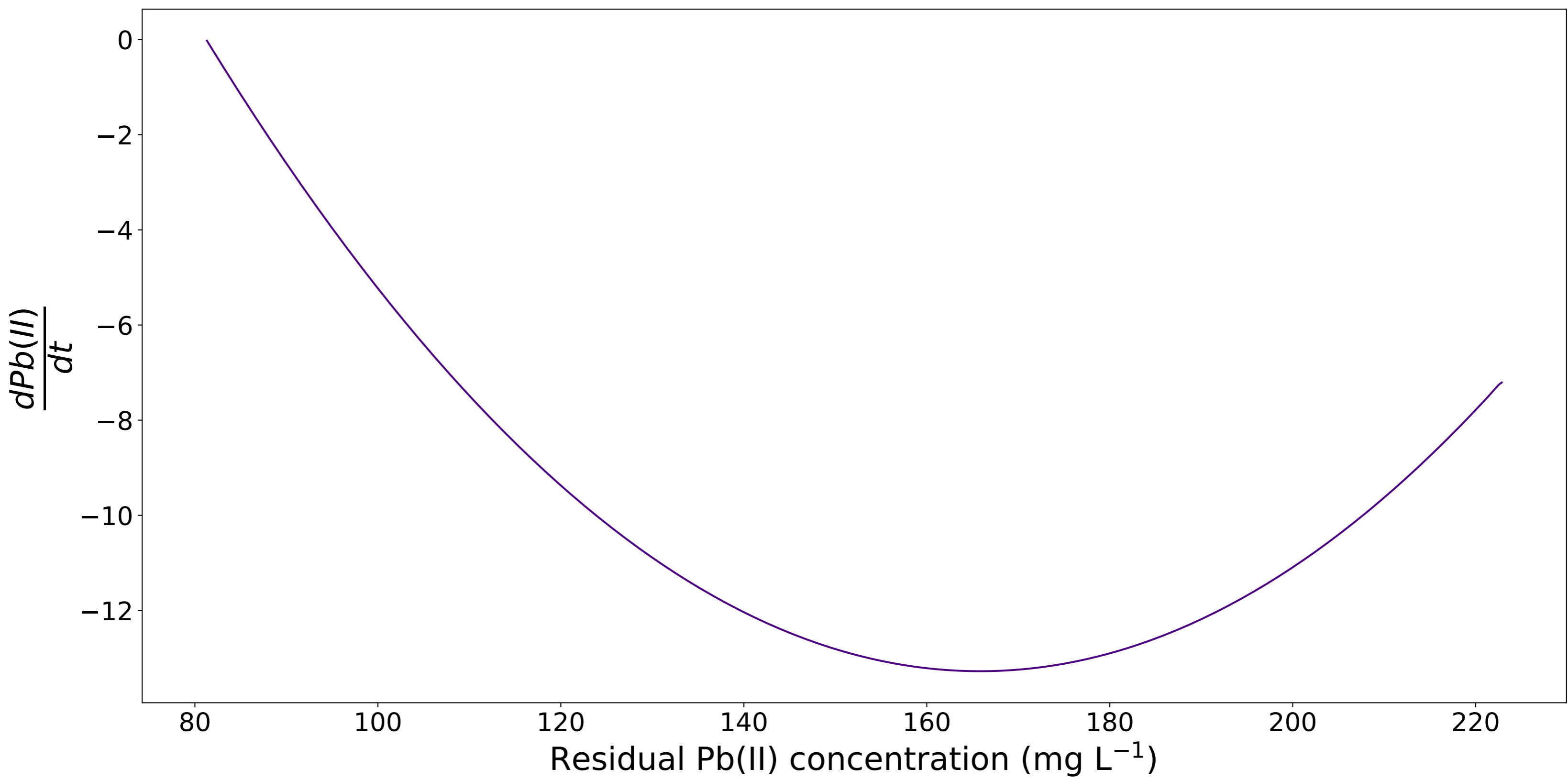

Supplement: Supplementary file 1 [file ijms-23-12255-s001.zip › Definitions/dPb_Pb_C250.pdf]

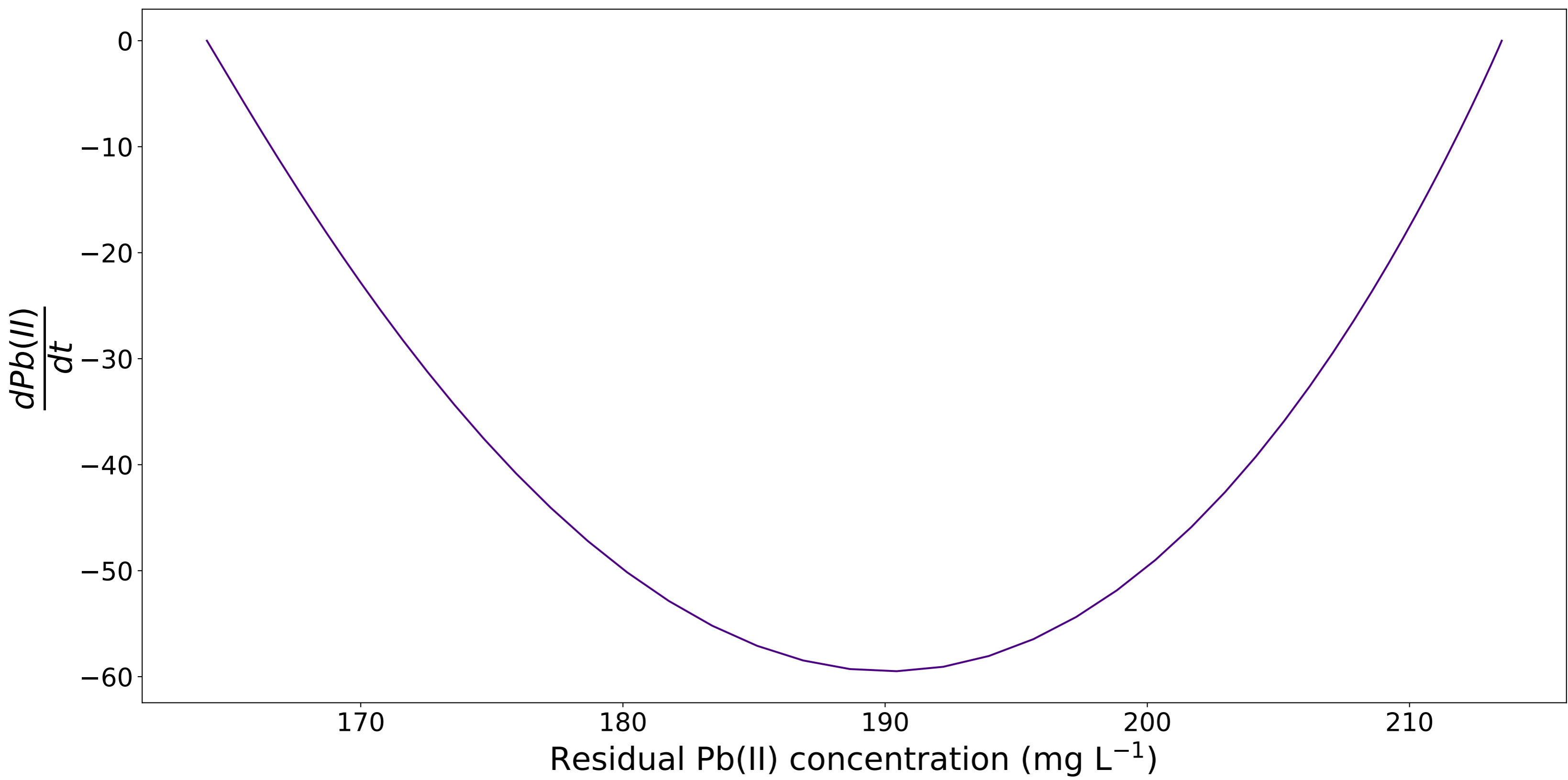

Supplement: Supplementary file 1 [file ijms-23-12255-s001.zip › Definitions/dPb_Pb_C500.pdf]

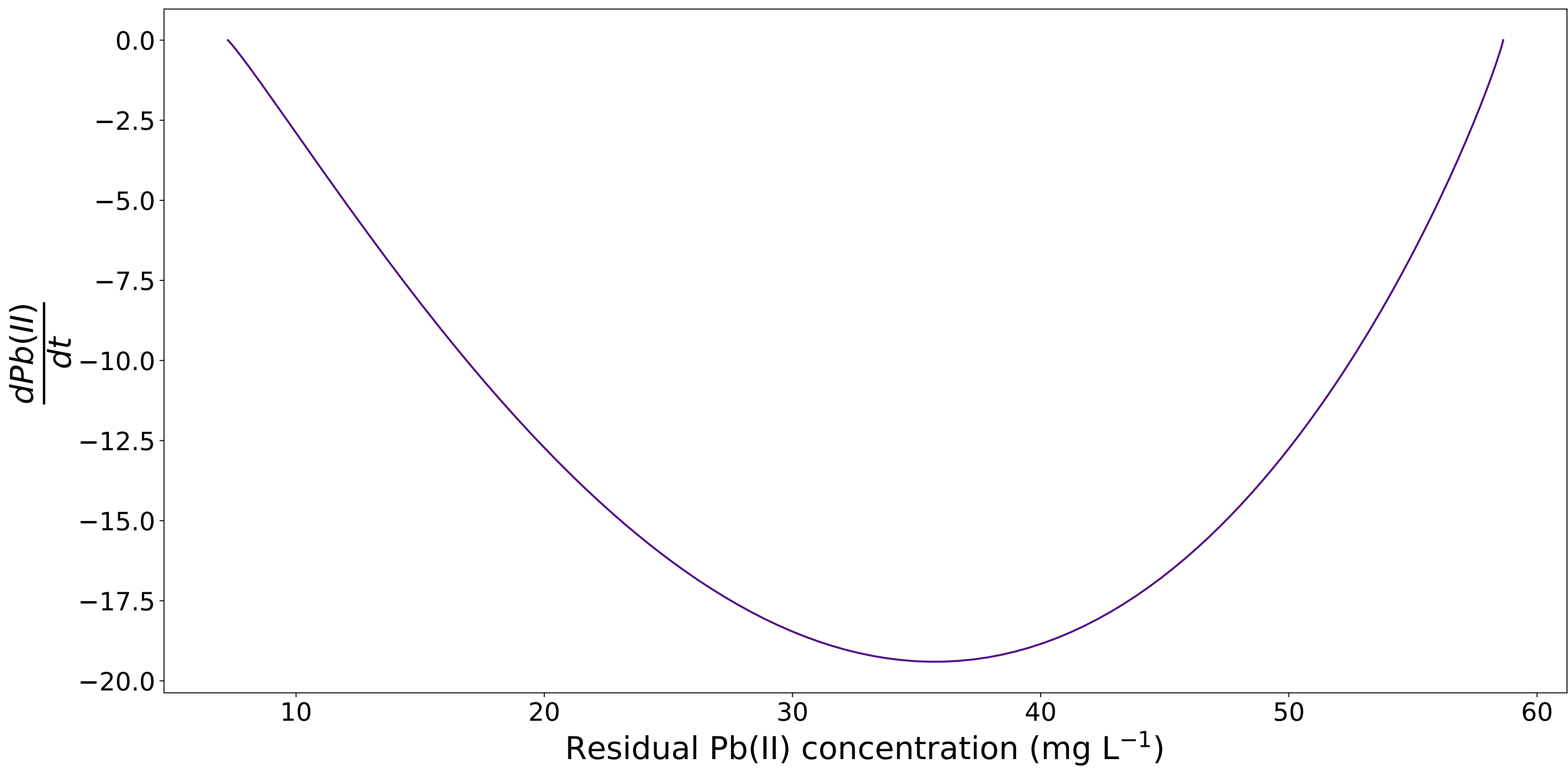

Supplement: Supplementary file 1 [file ijms-23-12255-s001.zip › Definitions/dPb_Pb_C80.pdf]

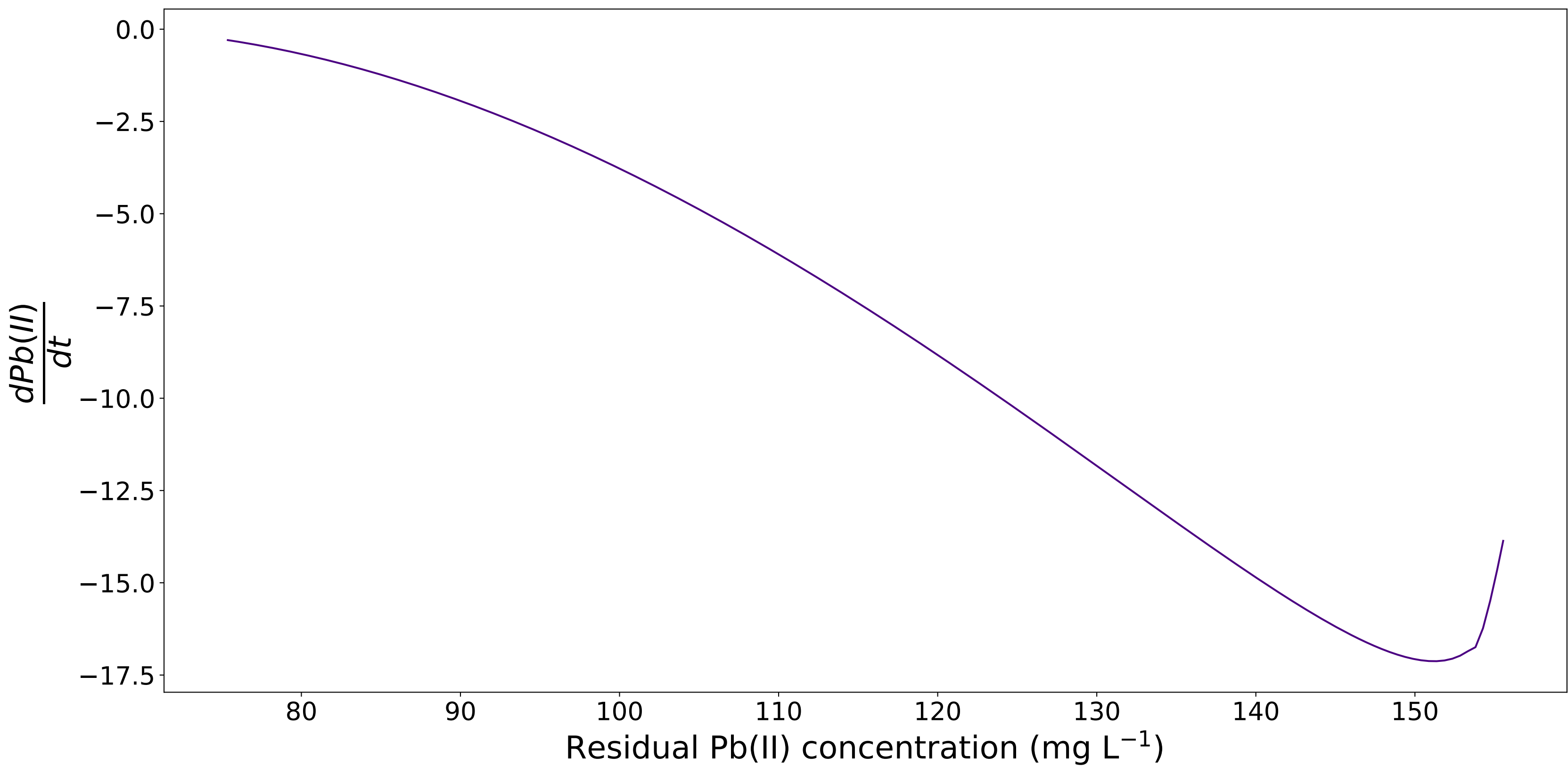

Supplement: Supplementary file 1 [file ijms-23-12255-s001.zip › Definitions/dPb_Pb_K250.pdf]

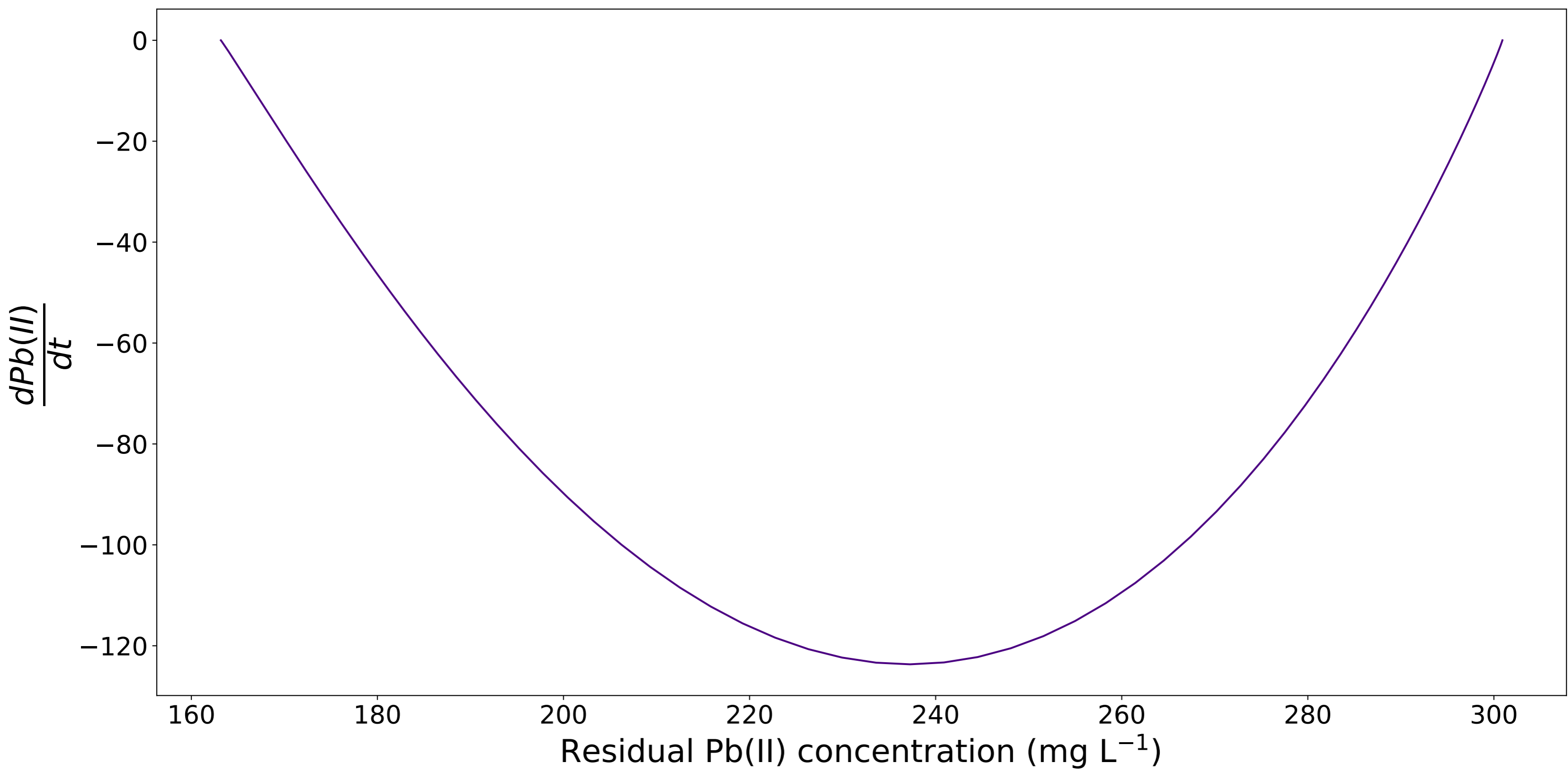

Supplement: Supplementary file 1 [file ijms-23-12255-s001.zip › Definitions/dPb_Pb_K500.pdf]

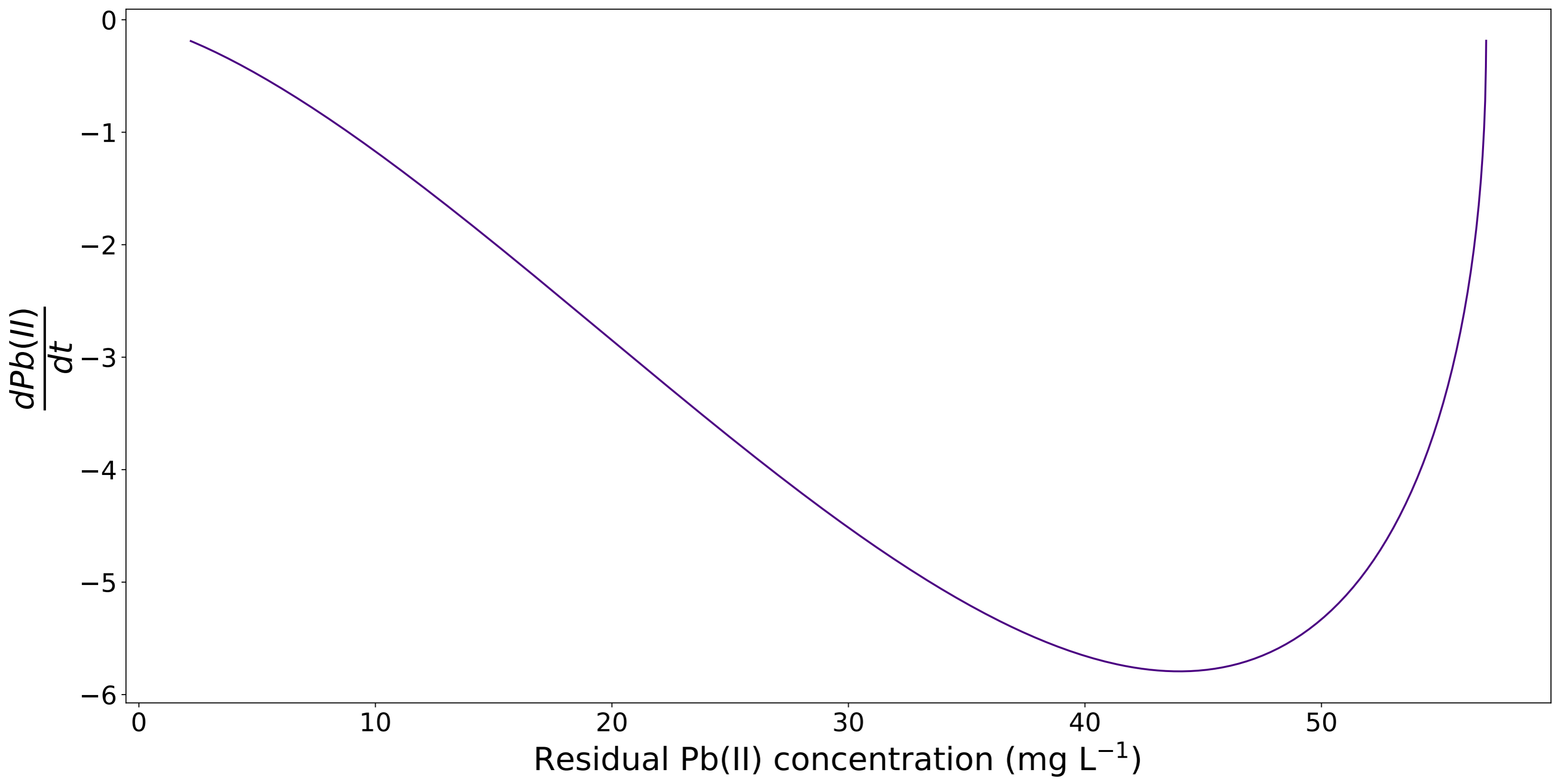

Supplement: Supplementary file 1 [file ijms-23-12255-s001.zip › Definitions/dPb_Pb_K80.pdf]

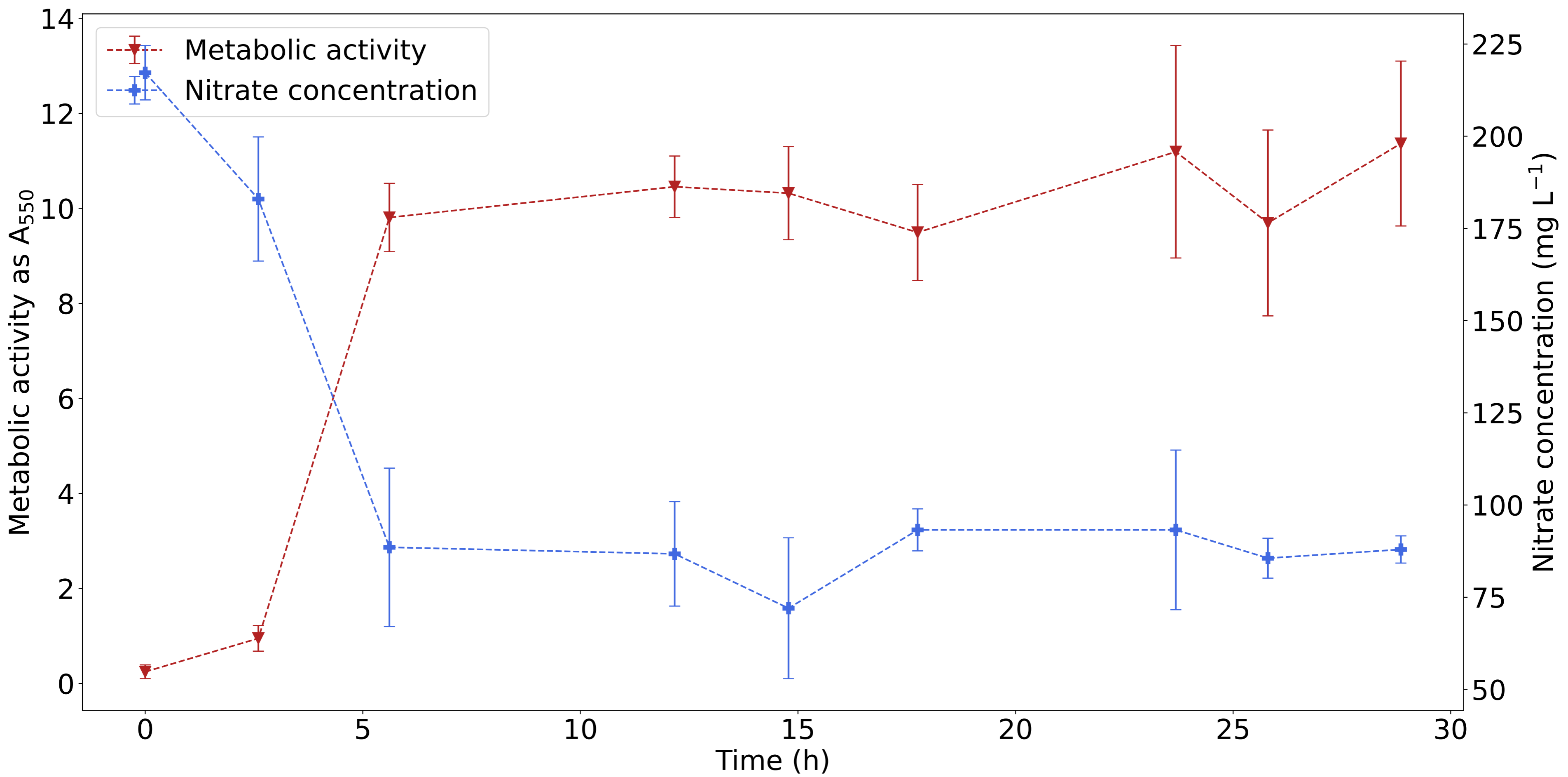

Supplement: Supplementary file 1 [file ijms-23-12255-s001.zip › Definitions/K250_MA_N.pdf]

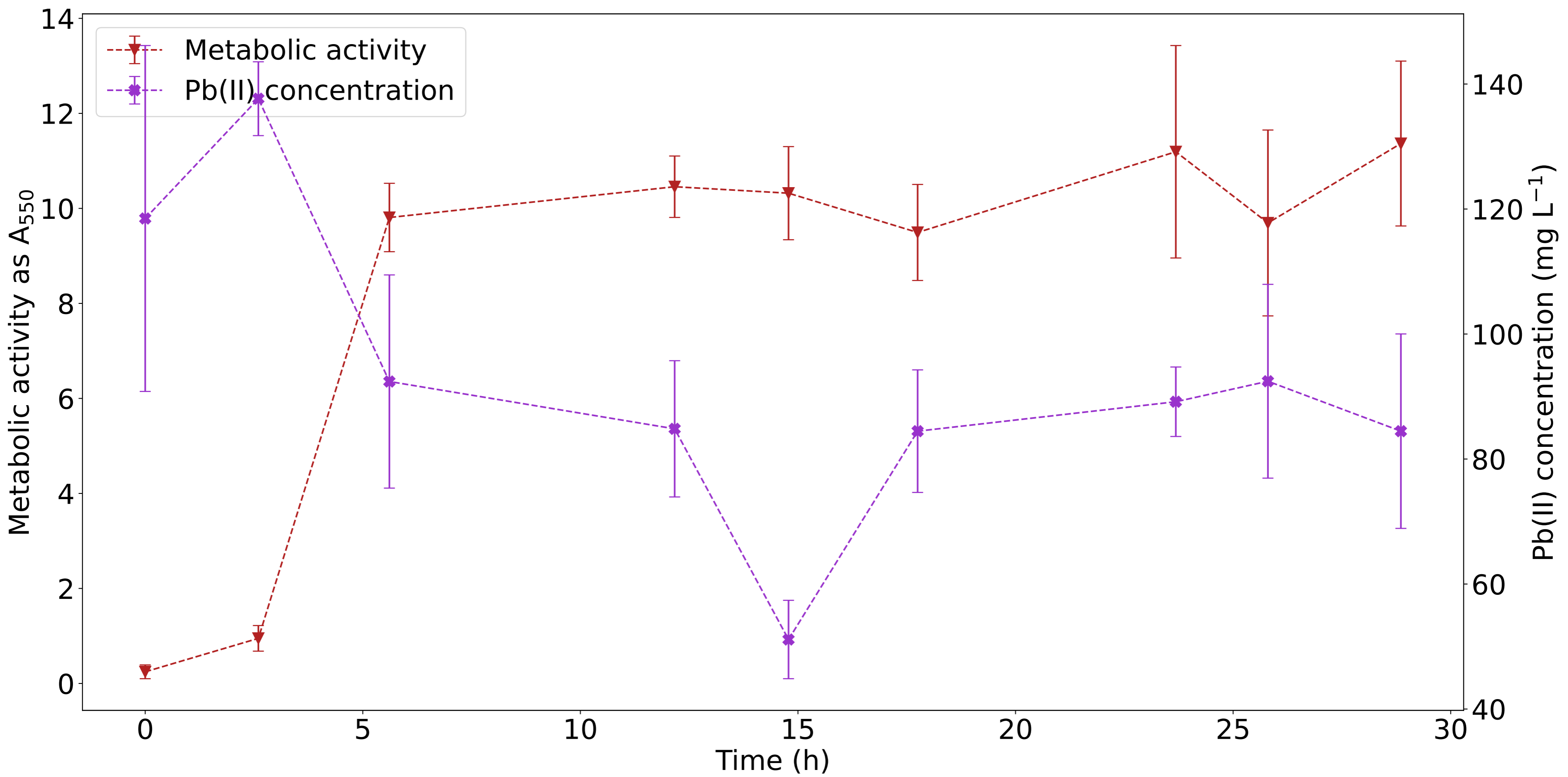

Supplement: Supplementary file 1 [file ijms-23-12255-s001.zip › Definitions/K250_MA_Pb.pdf]

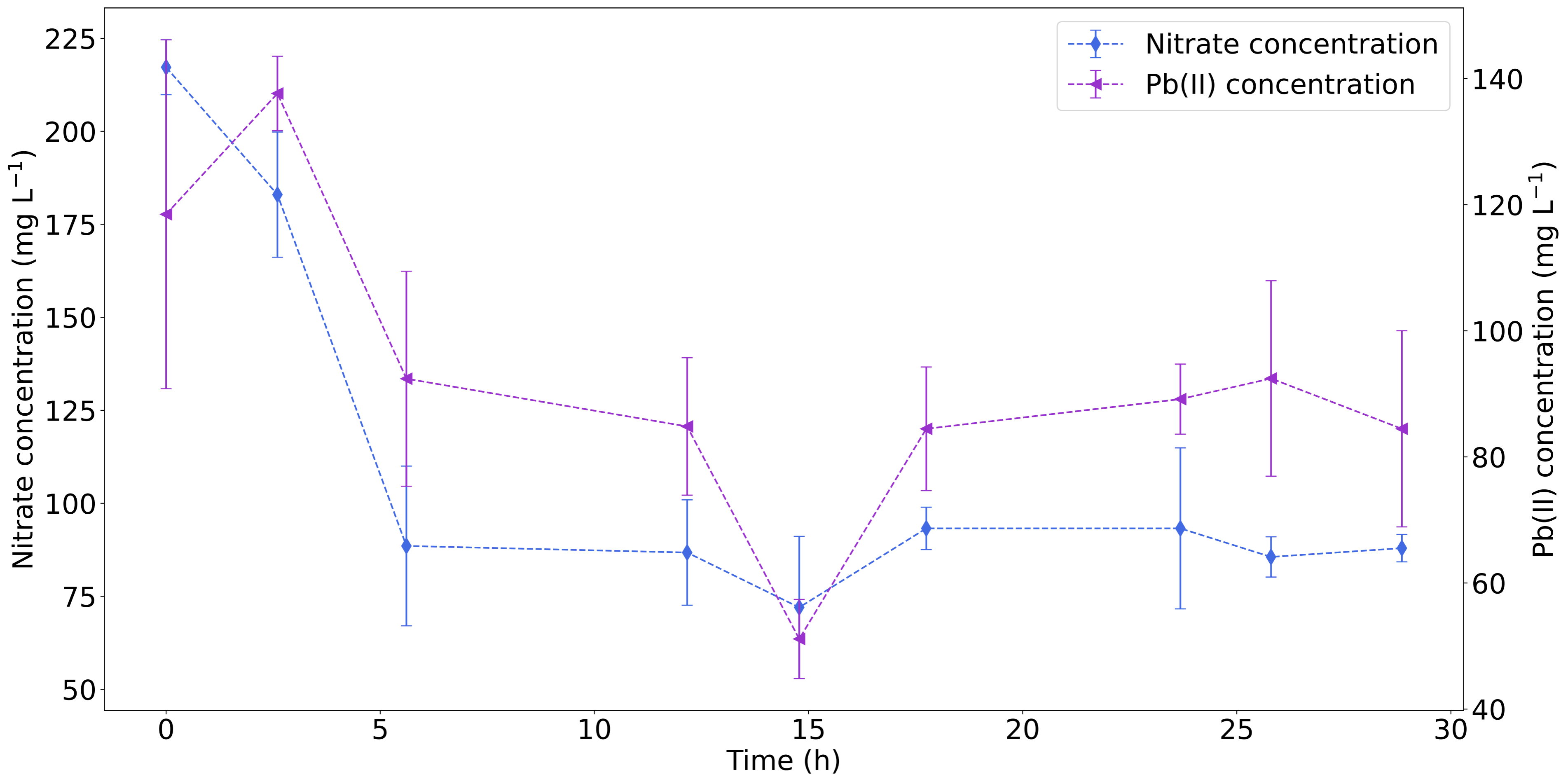

Supplement: Supplementary file 1 [file ijms-23-12255-s001.zip › Definitions/K250_N_Pb.pdf]

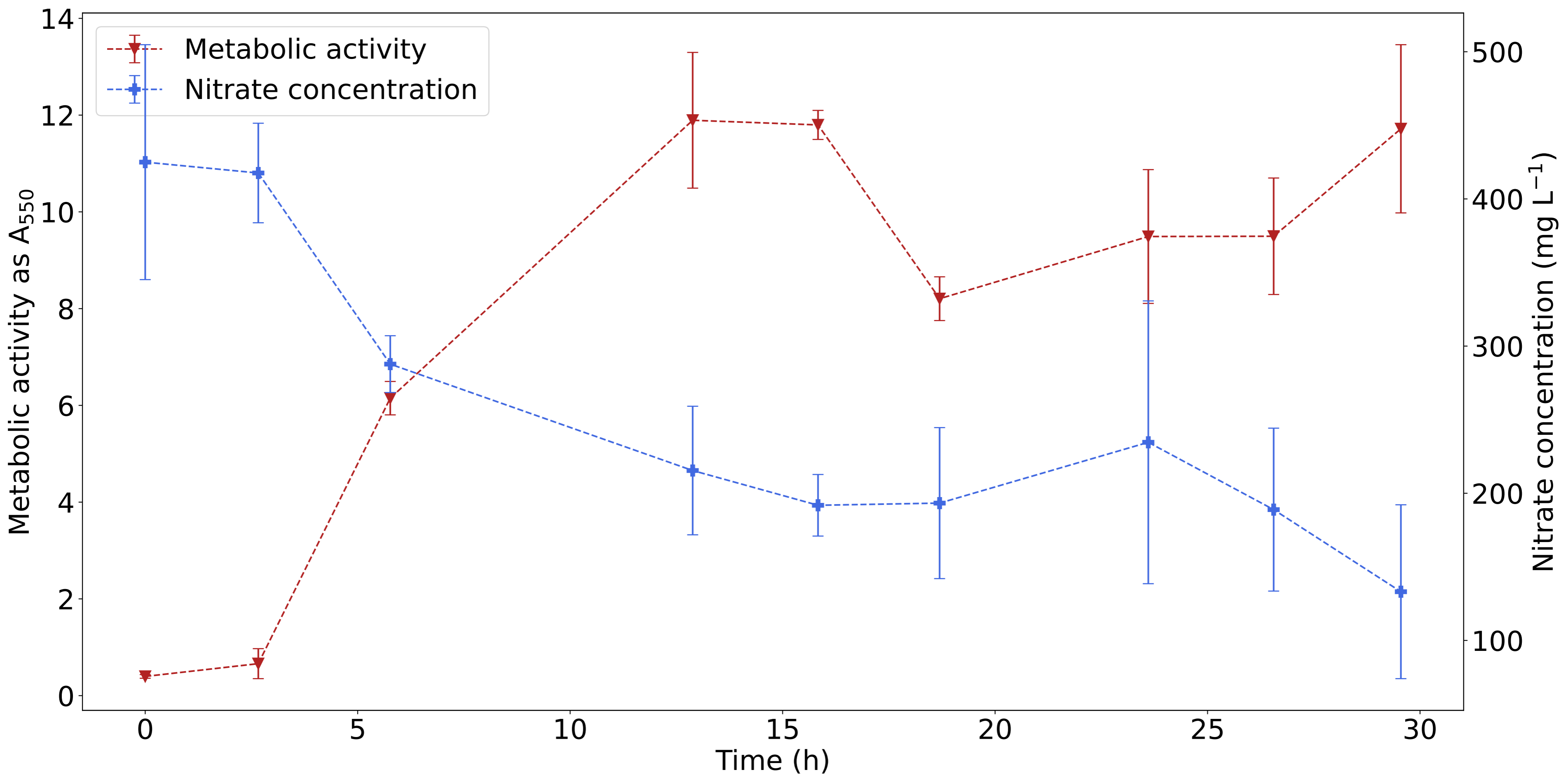

Supplement: Supplementary file 1 [file ijms-23-12255-s001.zip › Definitions/K500_MA_N.pdf]

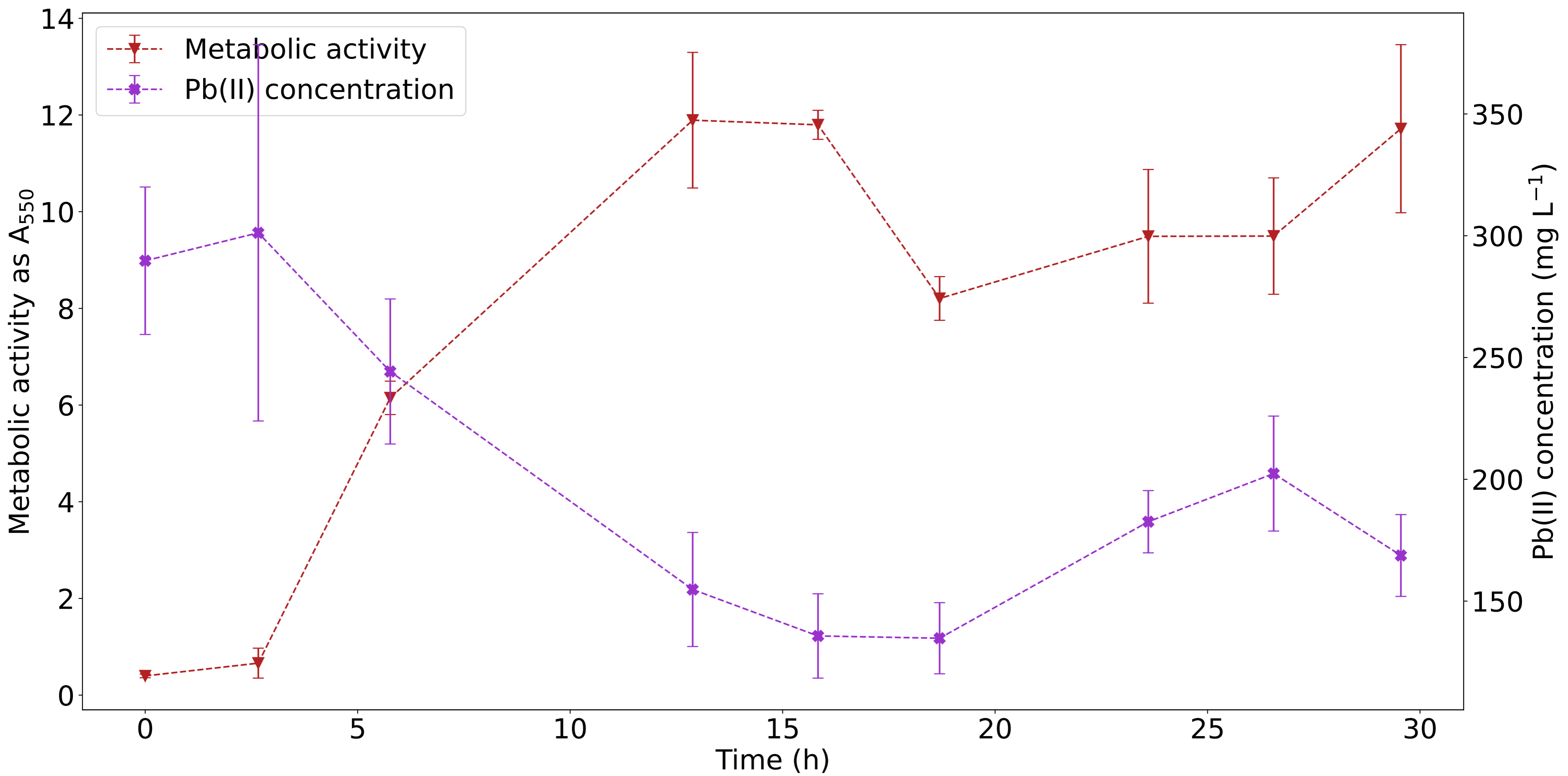

Supplement: Supplementary file 1 [file ijms-23-12255-s001.zip › Definitions/K500_MA_Pb.pdf]

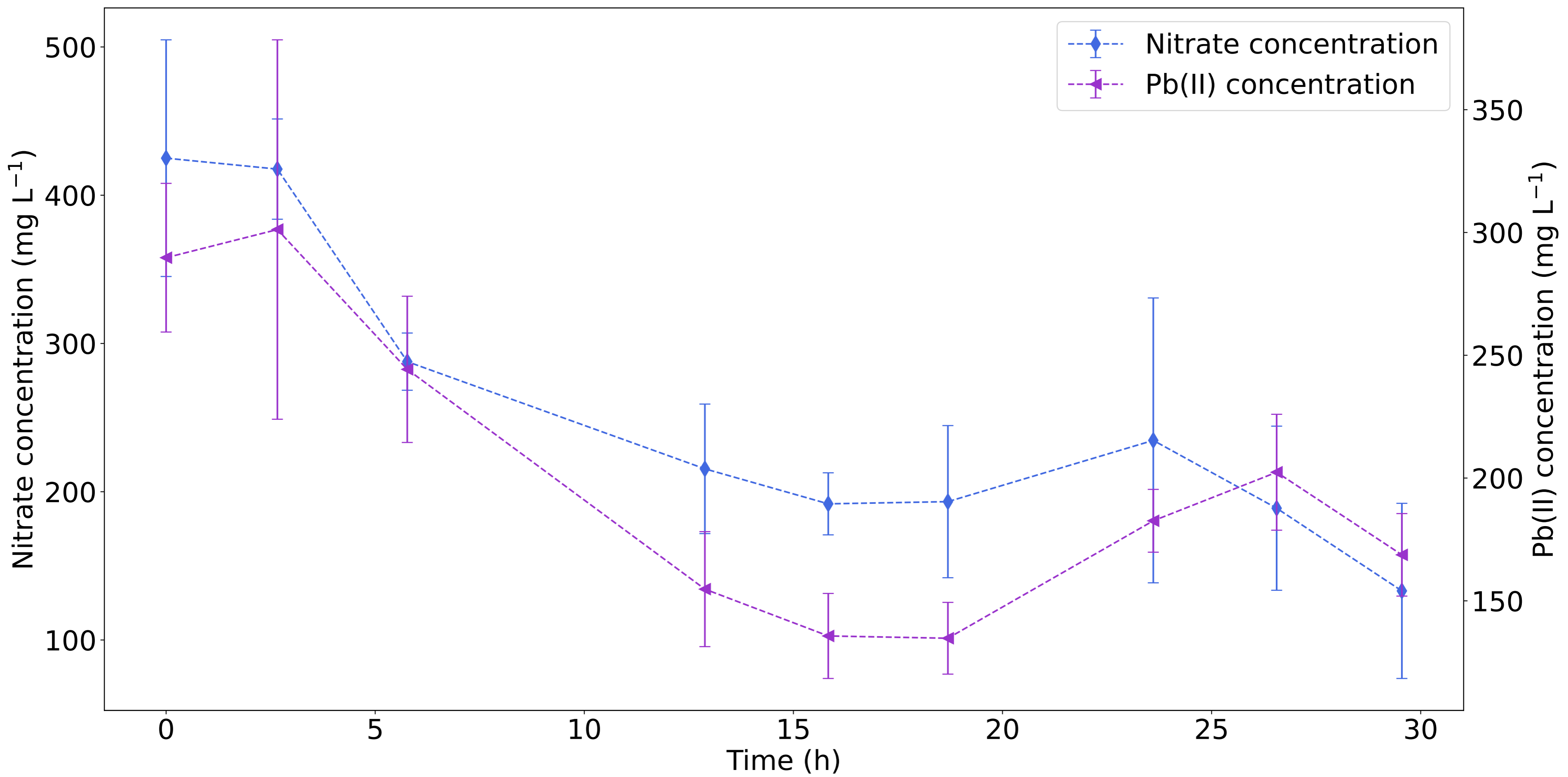

Supplement: Supplementary file 1 [file ijms-23-12255-s001.zip › Definitions/K500_N_Pb.pdf]

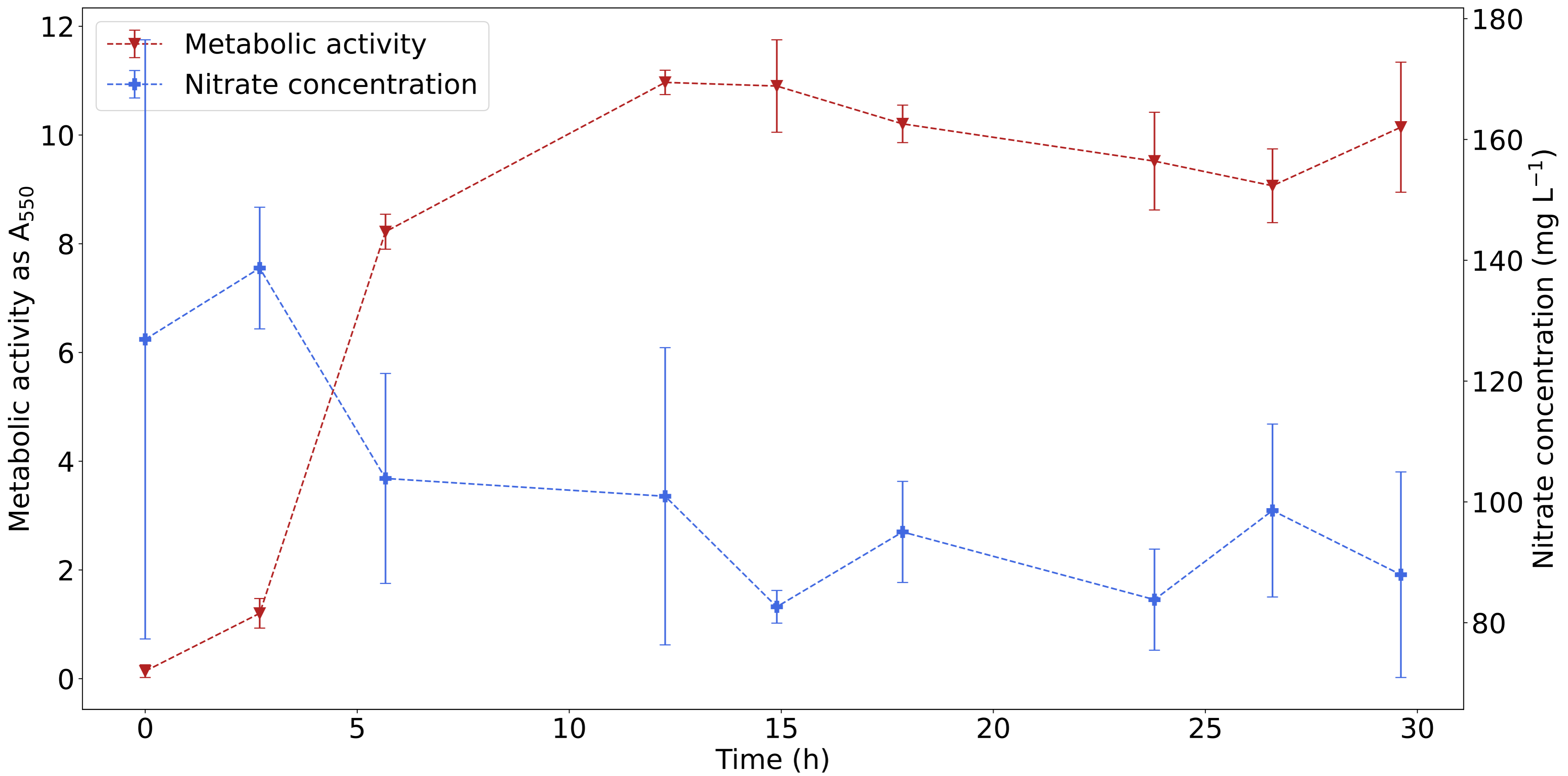

Supplement: Supplementary file 1 [file ijms-23-12255-s001.zip › Definitions/K80_MA_N.pdf]

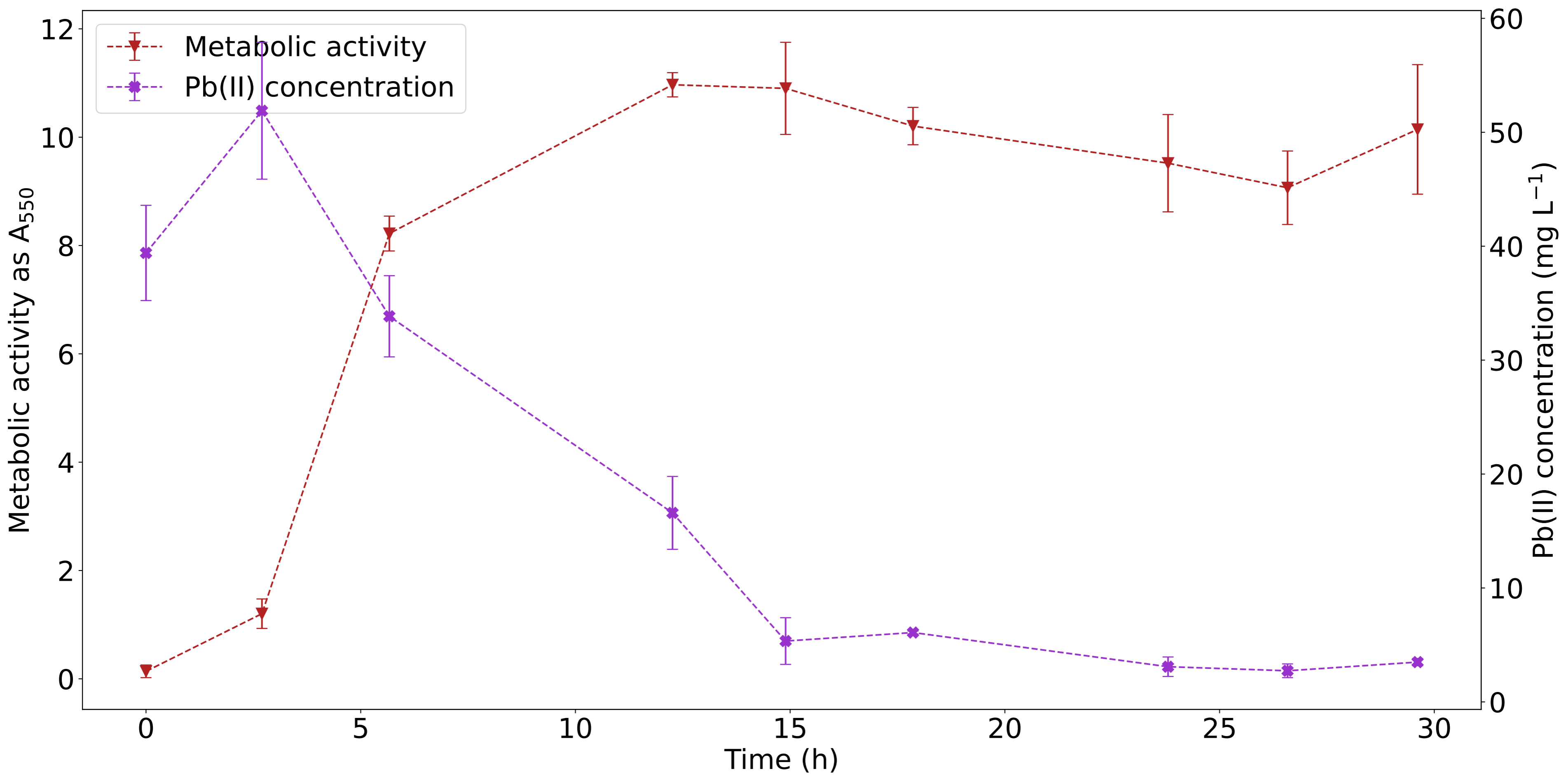

Supplement: Supplementary file 1 [file ijms-23-12255-s001.zip › Definitions/K80_MA_Pb.pdf]

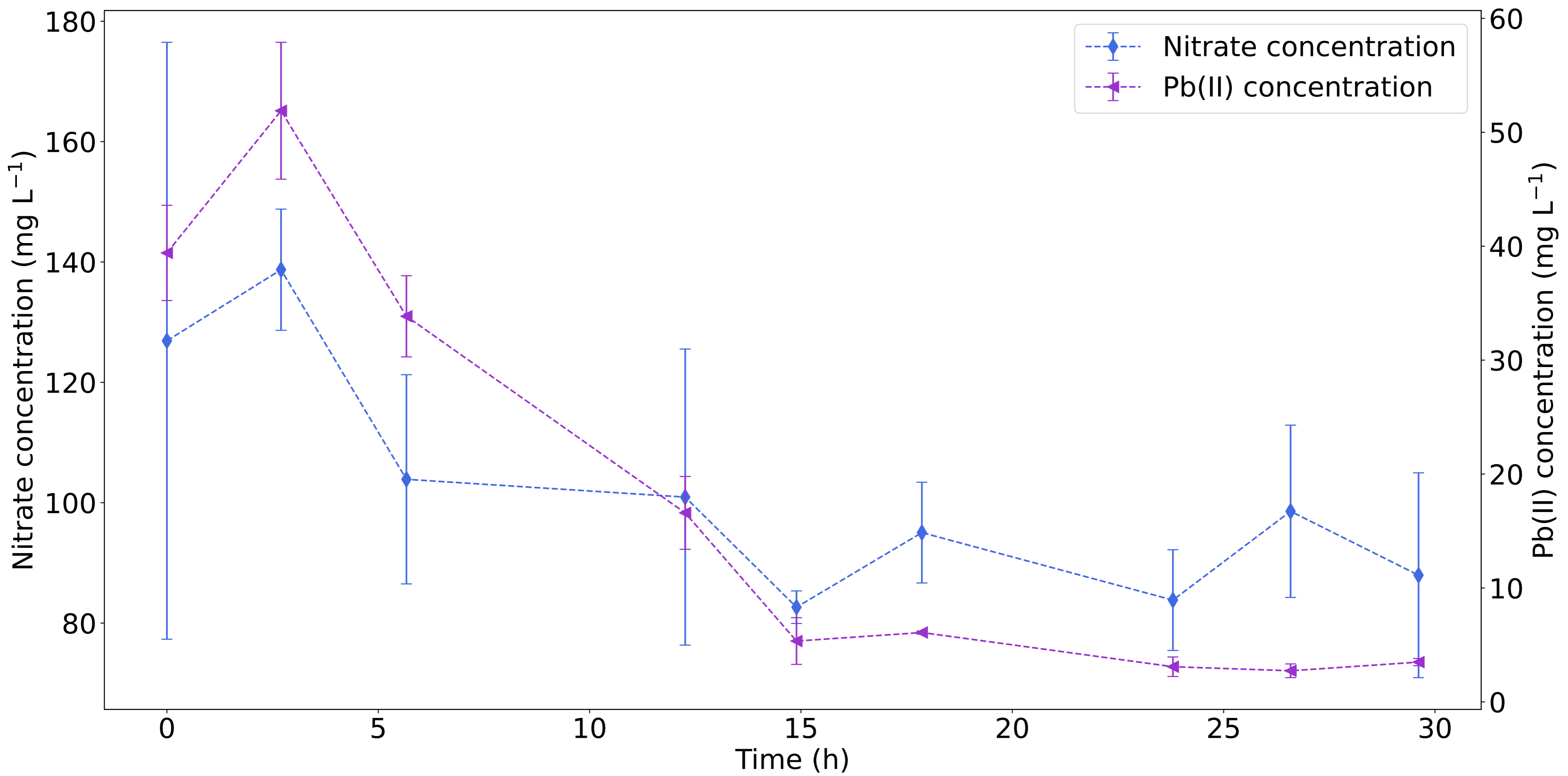

Supplement: Supplementary file 1 [file ijms-23-12255-s001.zip › Definitions/K80_N_Pb.pdf]

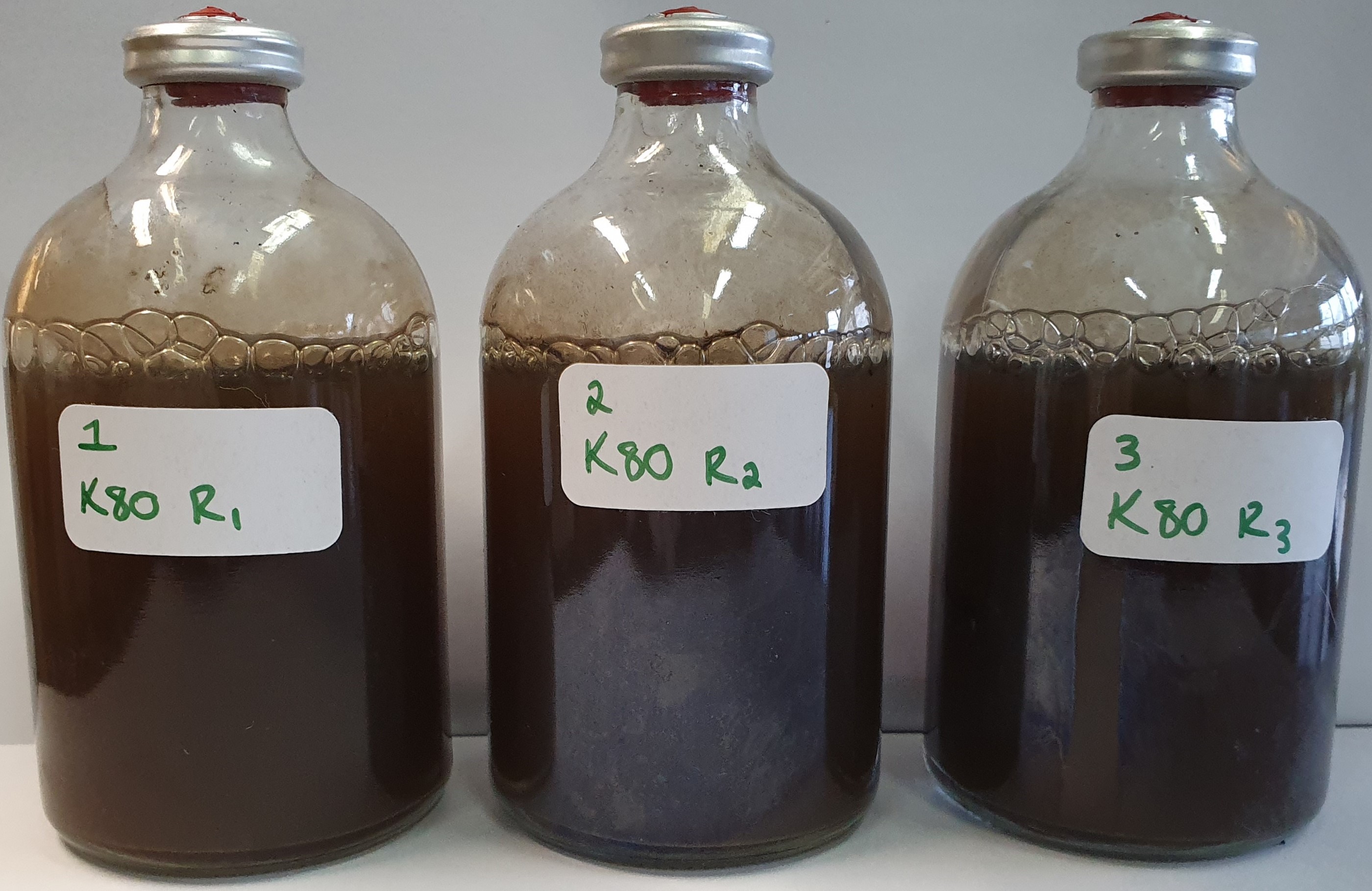

Supplement: Supplementary file 1 [file ijms-23-12255-s001.zip › Definitions/K80_TF.jpg]

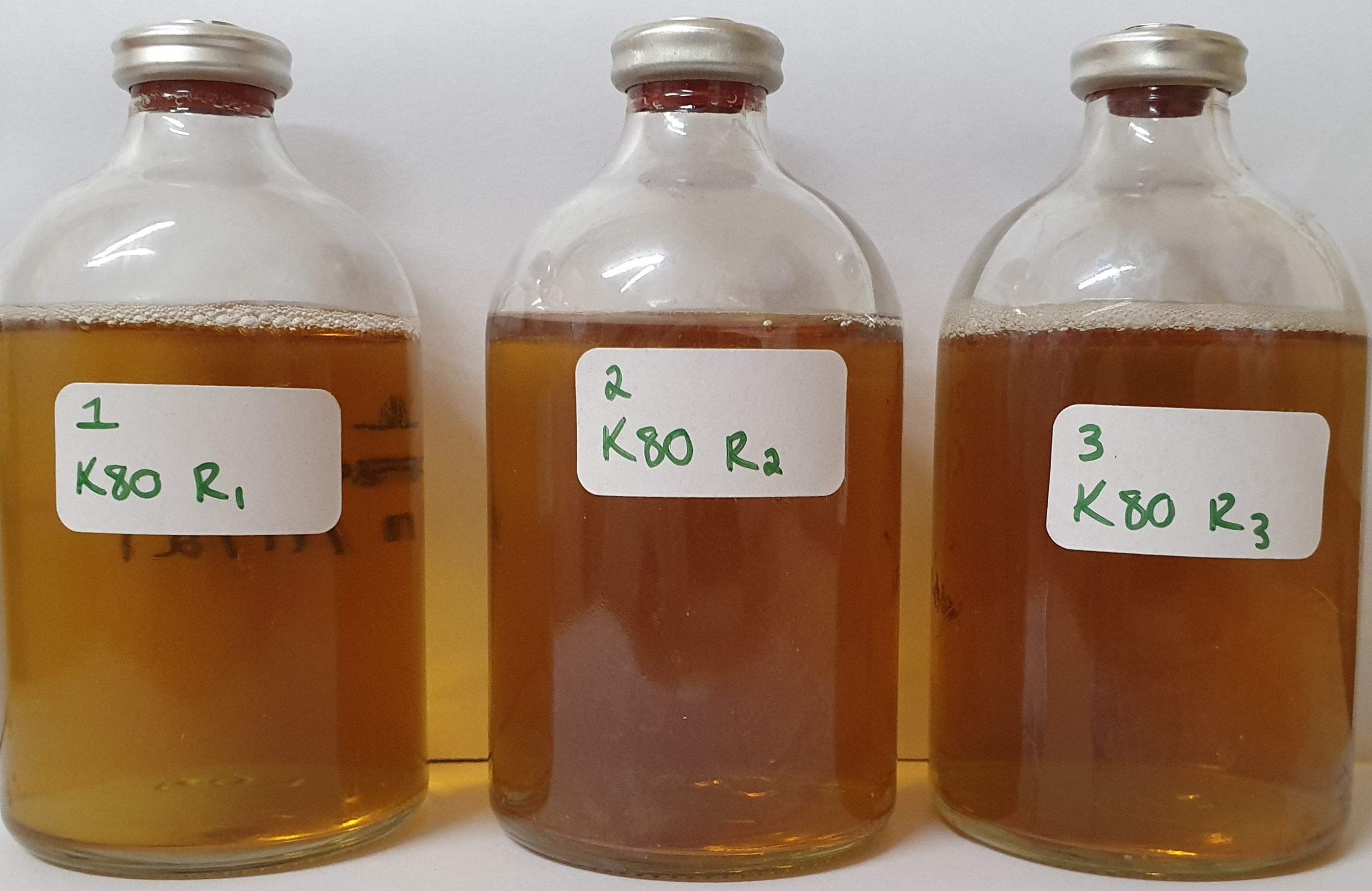

Supplement: Supplementary file 1 [file ijms-23-12255-s001.zip › Definitions/K80_TI.jpg]

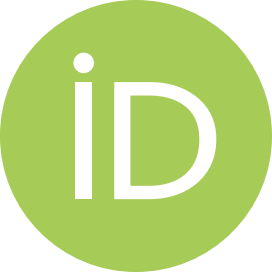

Supplement: Supplementary file 1 [file ijms-23-12255-s001.zip › Definitions/logo-orcid.pdf]

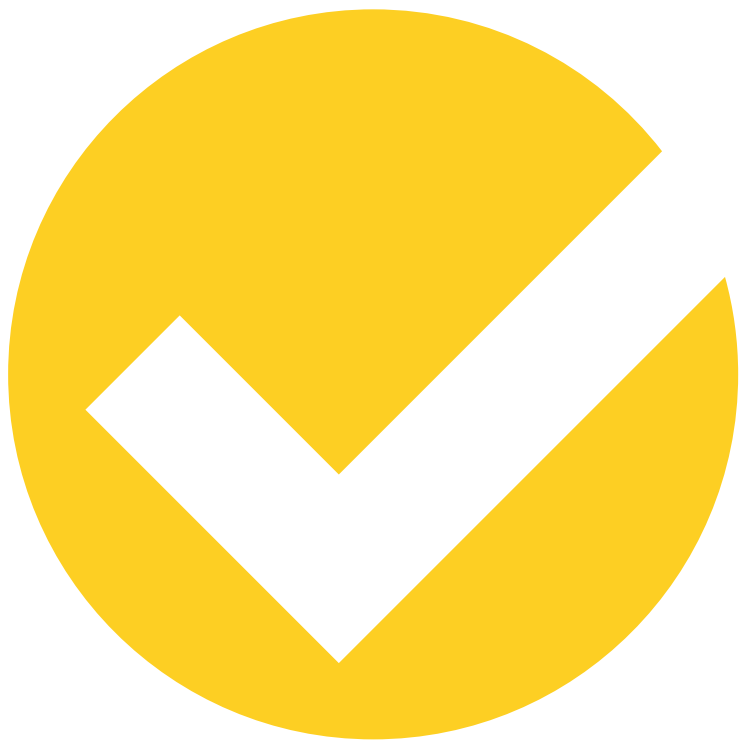

check for  
updates

Supplement: Supplementary file 1 [file ijms-23-12255-s001.zip › Definitions/logo-updates.pdf]

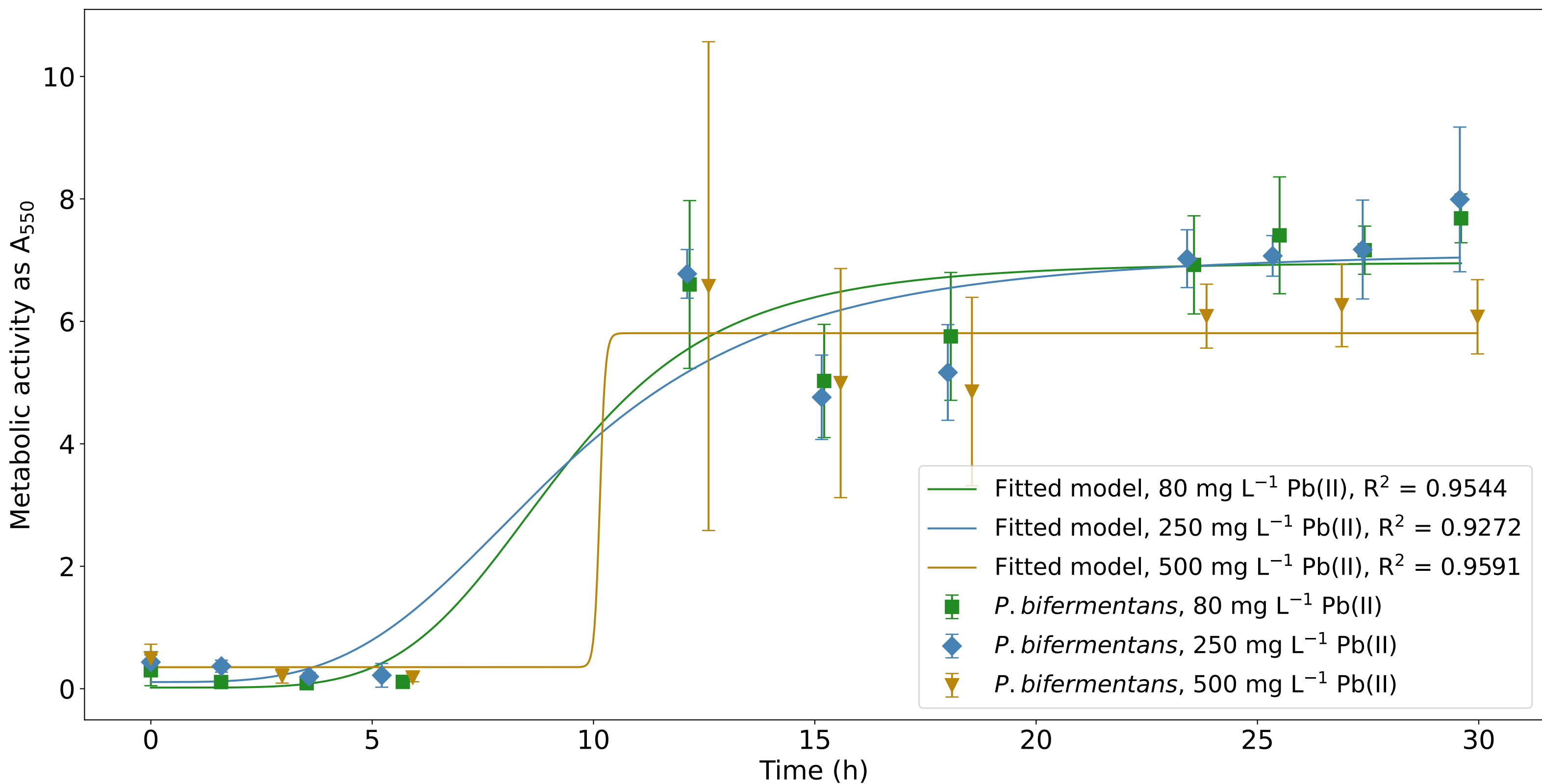

Supplement: Supplementary file 1 [file ijms-23-12255-s001.zip › Definitions/MA_C_Fitted.pdf]

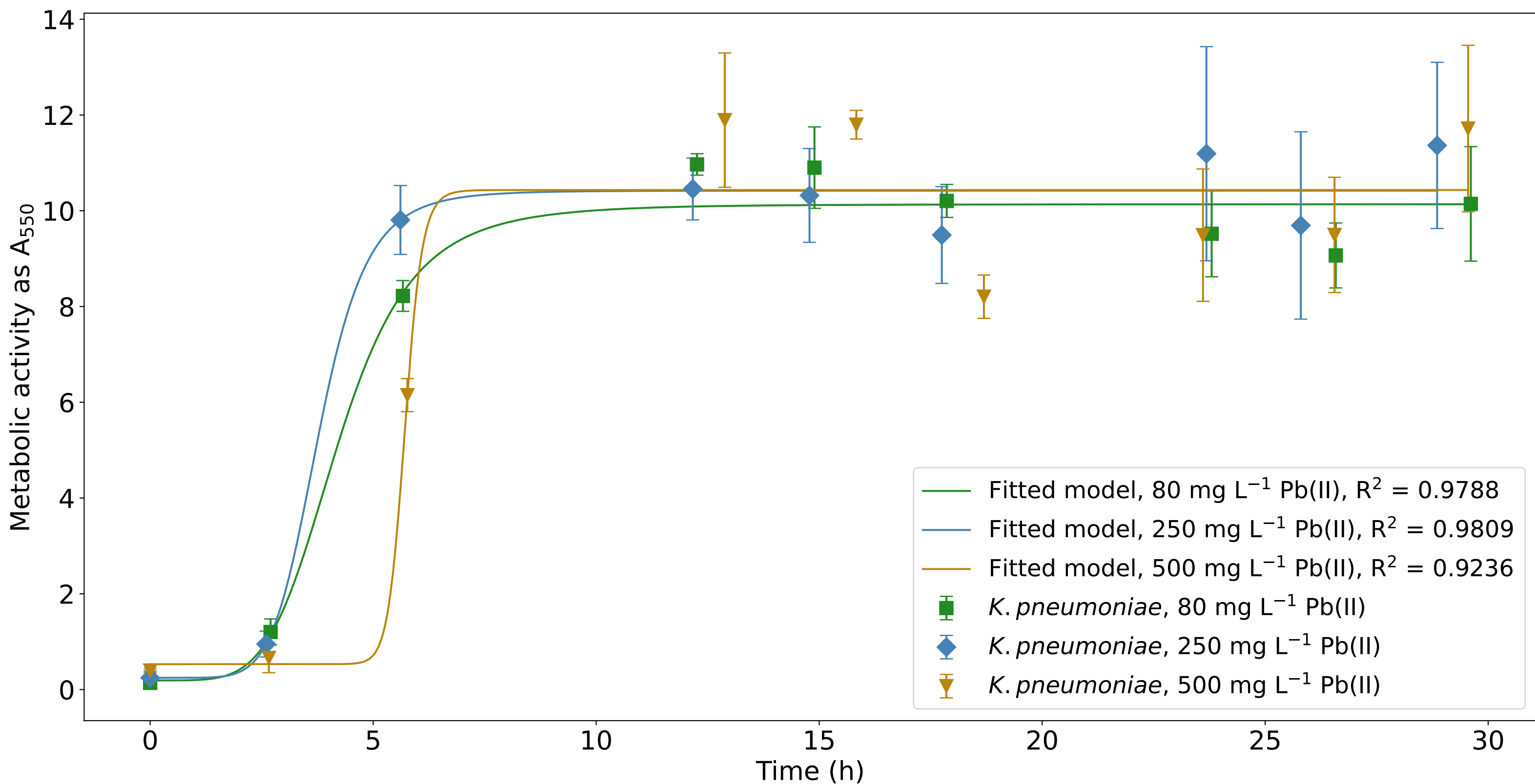

Supplement: Supplementary file 1 [file ijms-23-12255-s001.zip › Definitions/MA_K_Fitted.pdf]

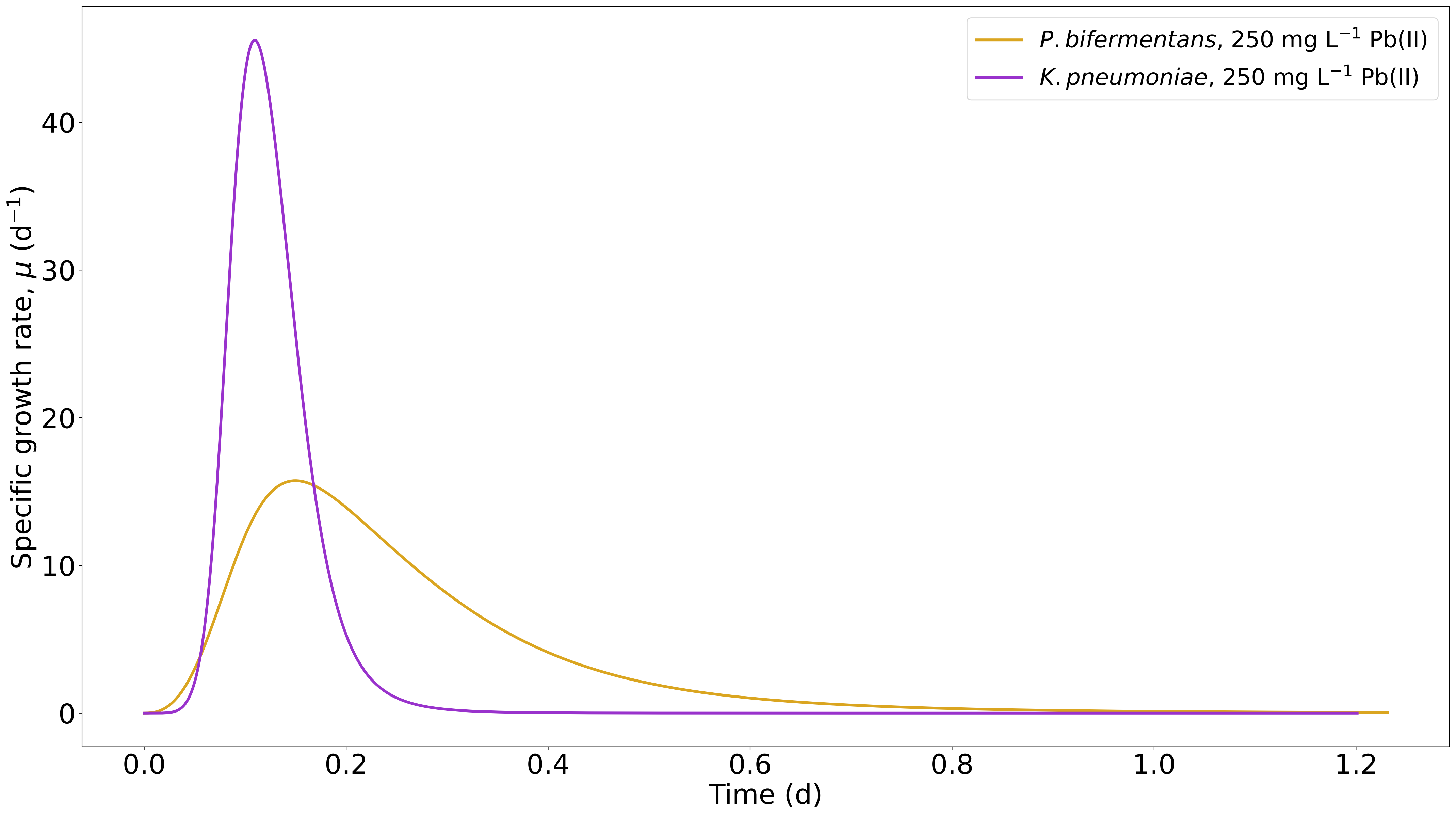

Supplement: Supplementary file 1 [file ijms-23-12255-s001.zip › Definitions/mu_250_n.pdf]

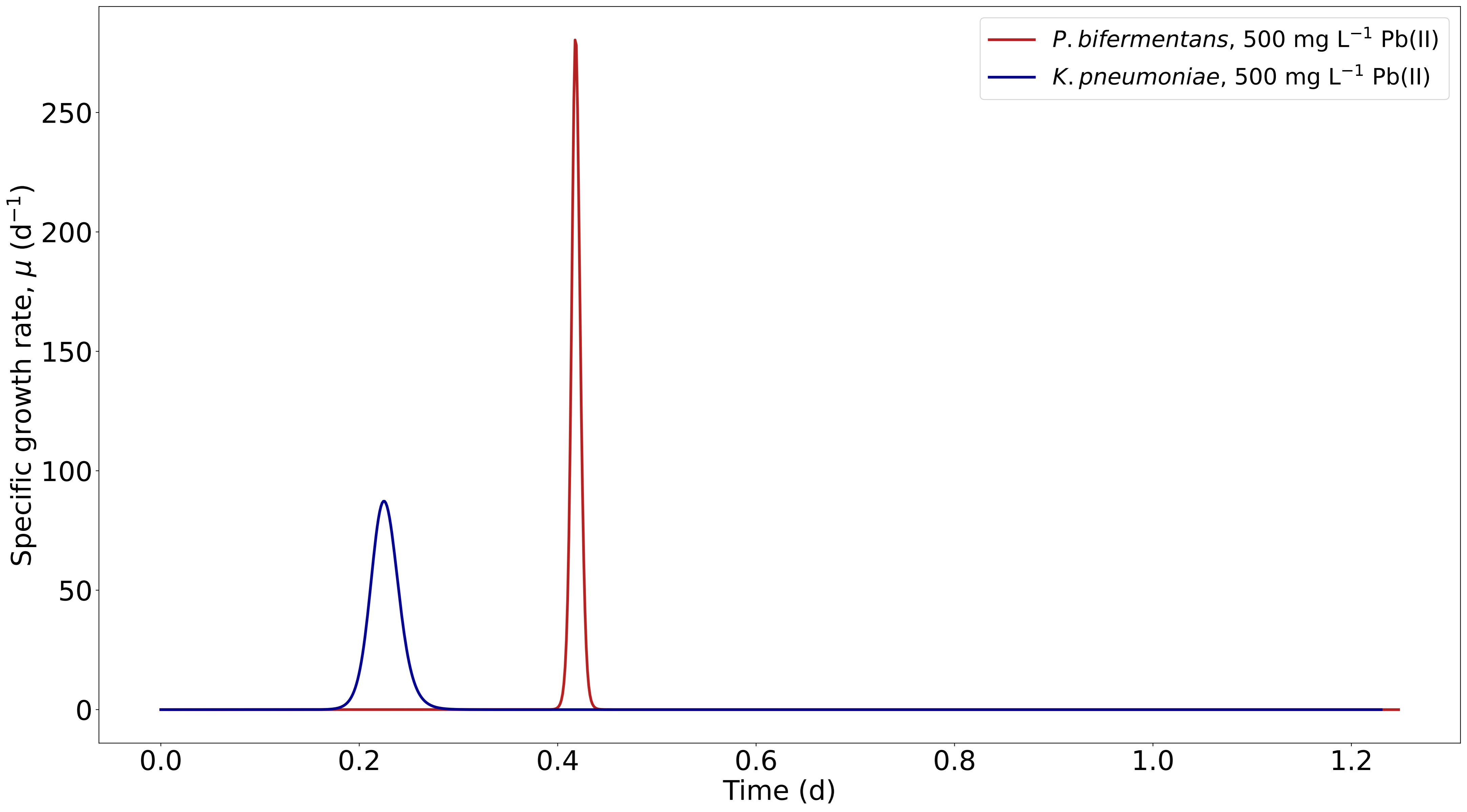

Supplement: Supplementary file 1 [file ijms-23-12255-s001.zip › Definitions/mu_500_n.pdf]

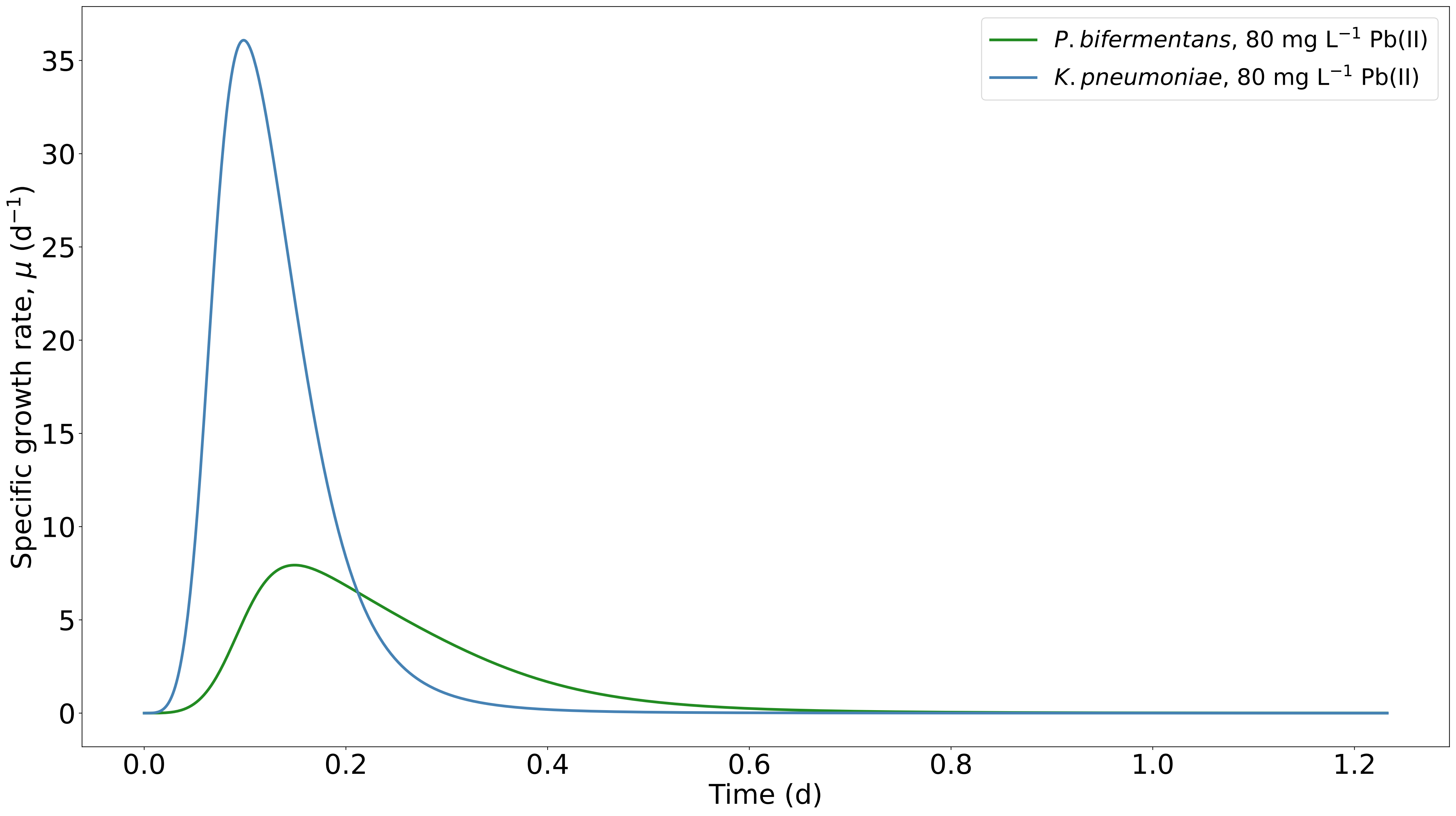

Supplement: Supplementary file 1 [file ijms-23-12255-s001.zip › Definitions/mu_80_n.pdf]

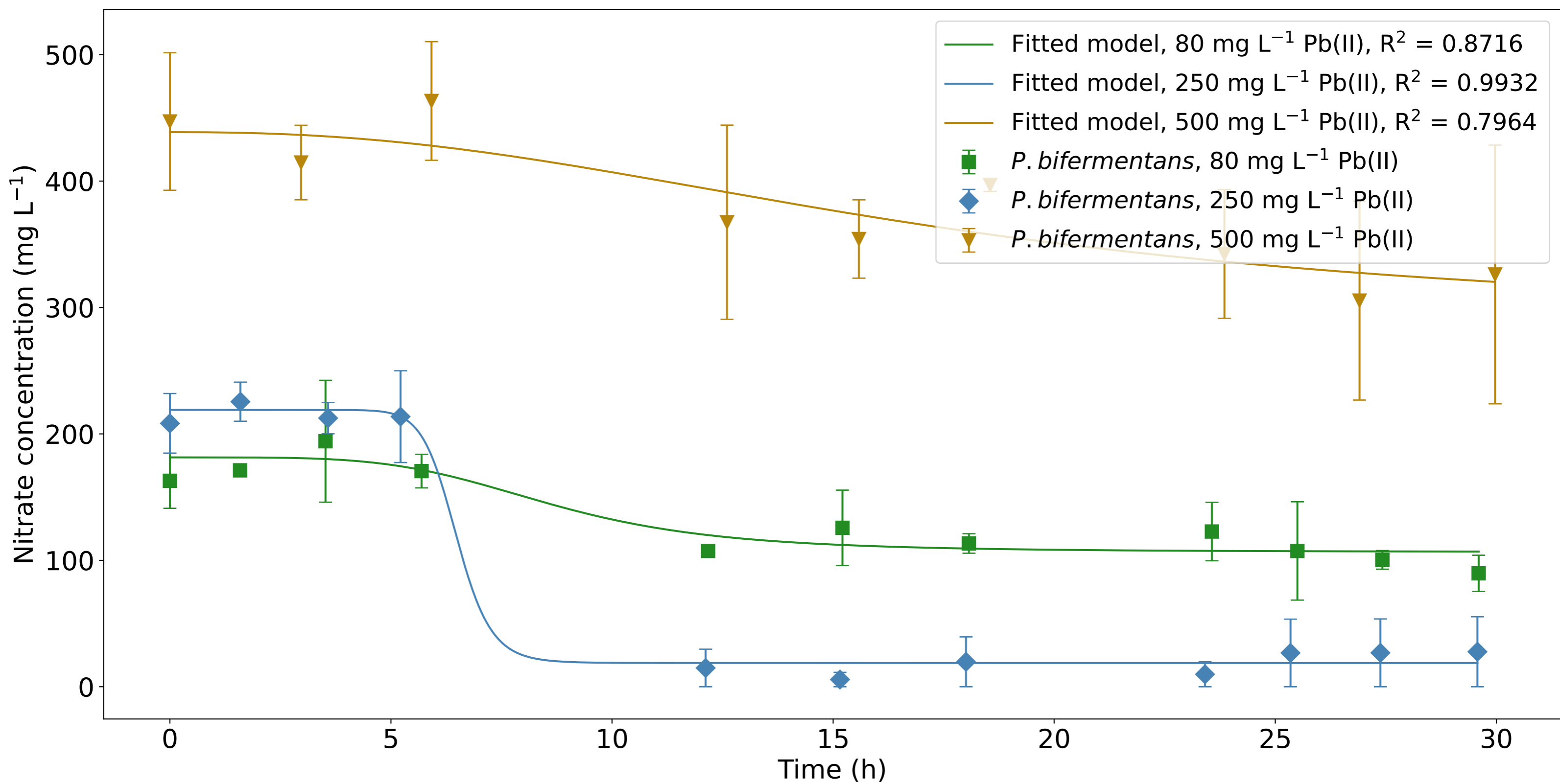

Supplement: Supplementary file 1 [file ijms-23-12255-s001.zip › Definitions/N_C_Fitted.pdf]

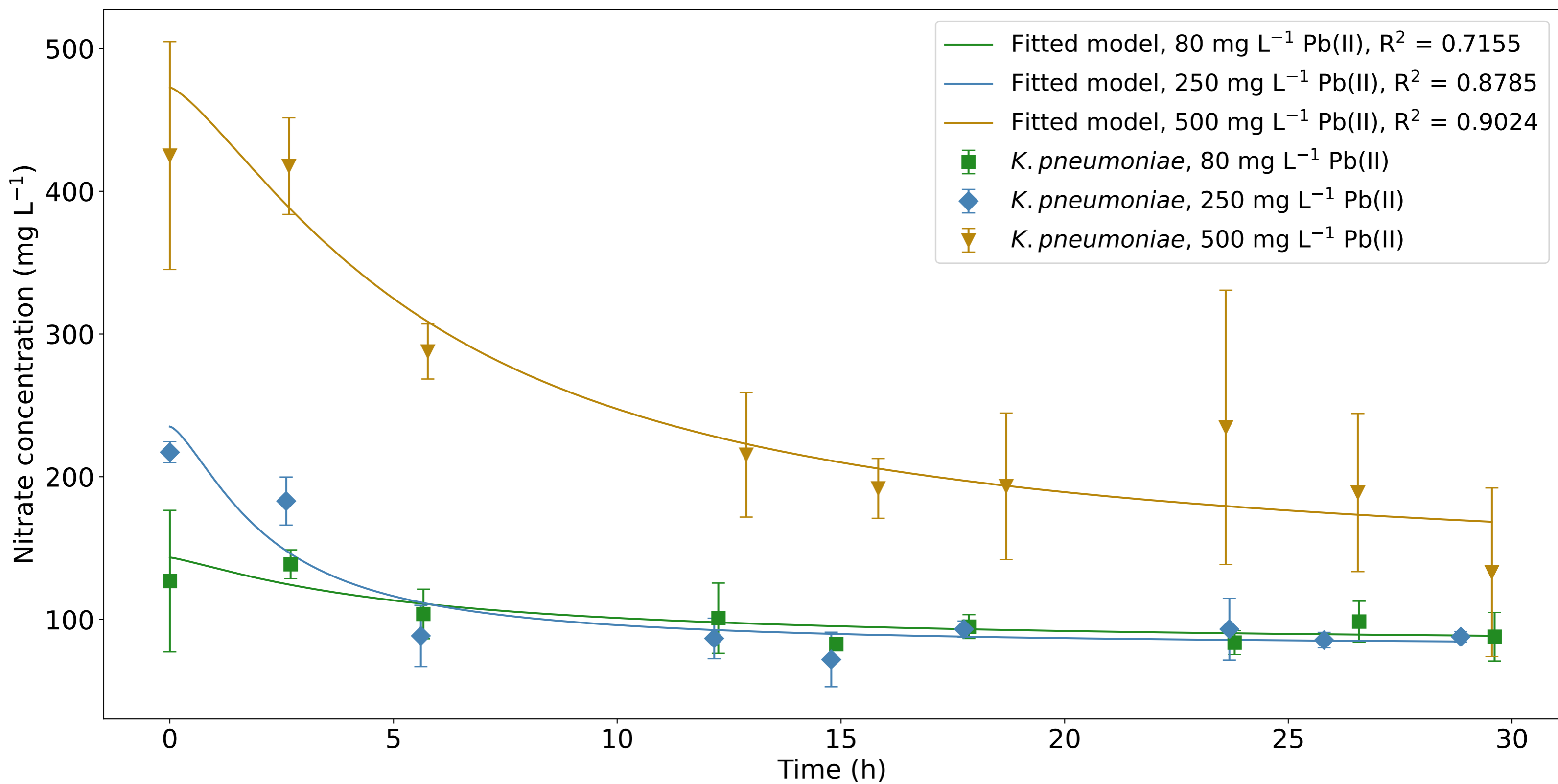

Supplement: Supplementary file 1 [file ijms-23-12255-s001.zip › Definitions/N_K_Fitted.pdf]

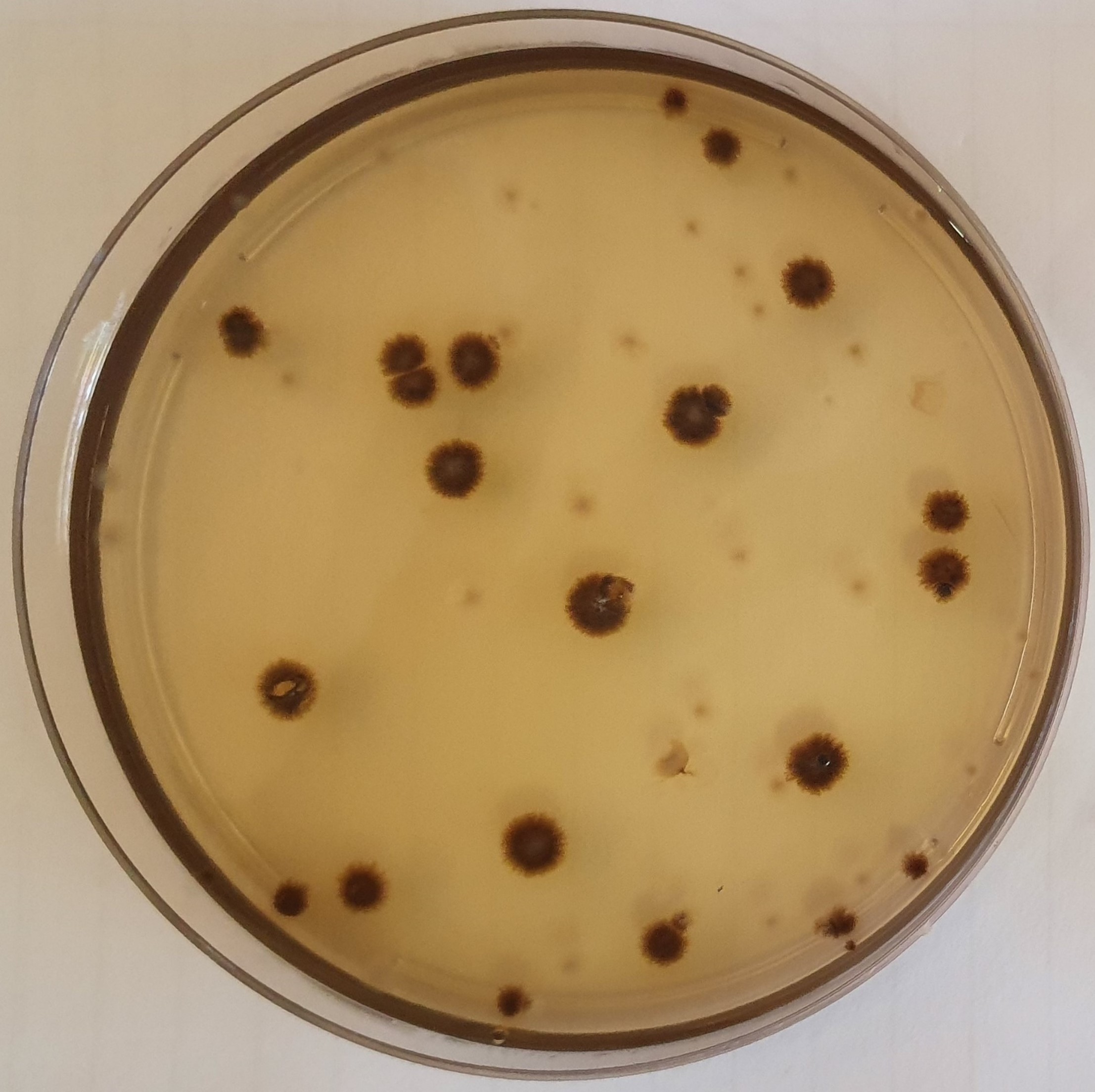

Supplement: Supplementary file 1 [file ijms-23-12255-s001.zip › Definitions/Para_1.jpg]

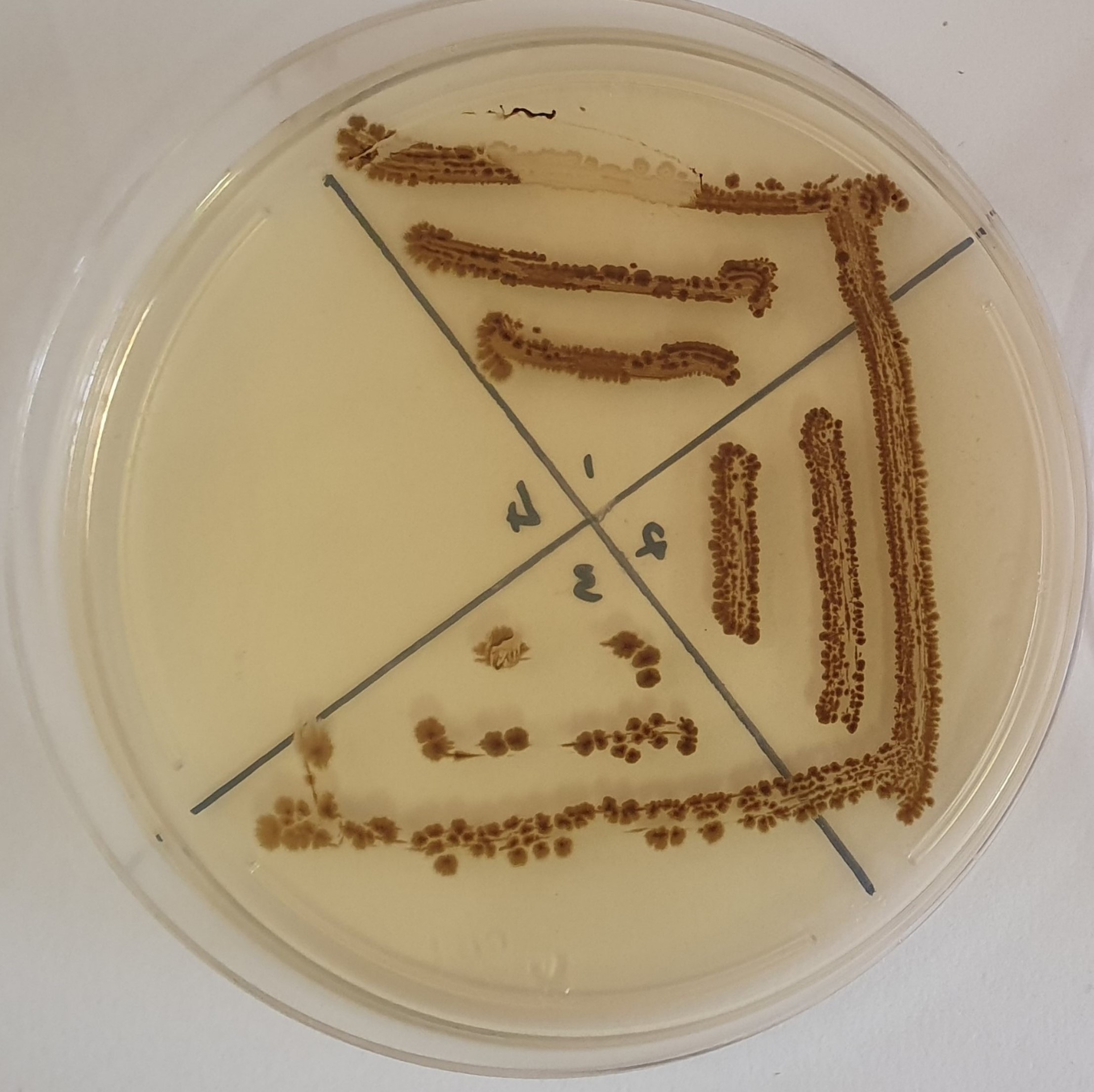

Supplement: Supplementary file 1 [file ijms-23-12255-s001.zip › Definitions/Para_2.jpg]

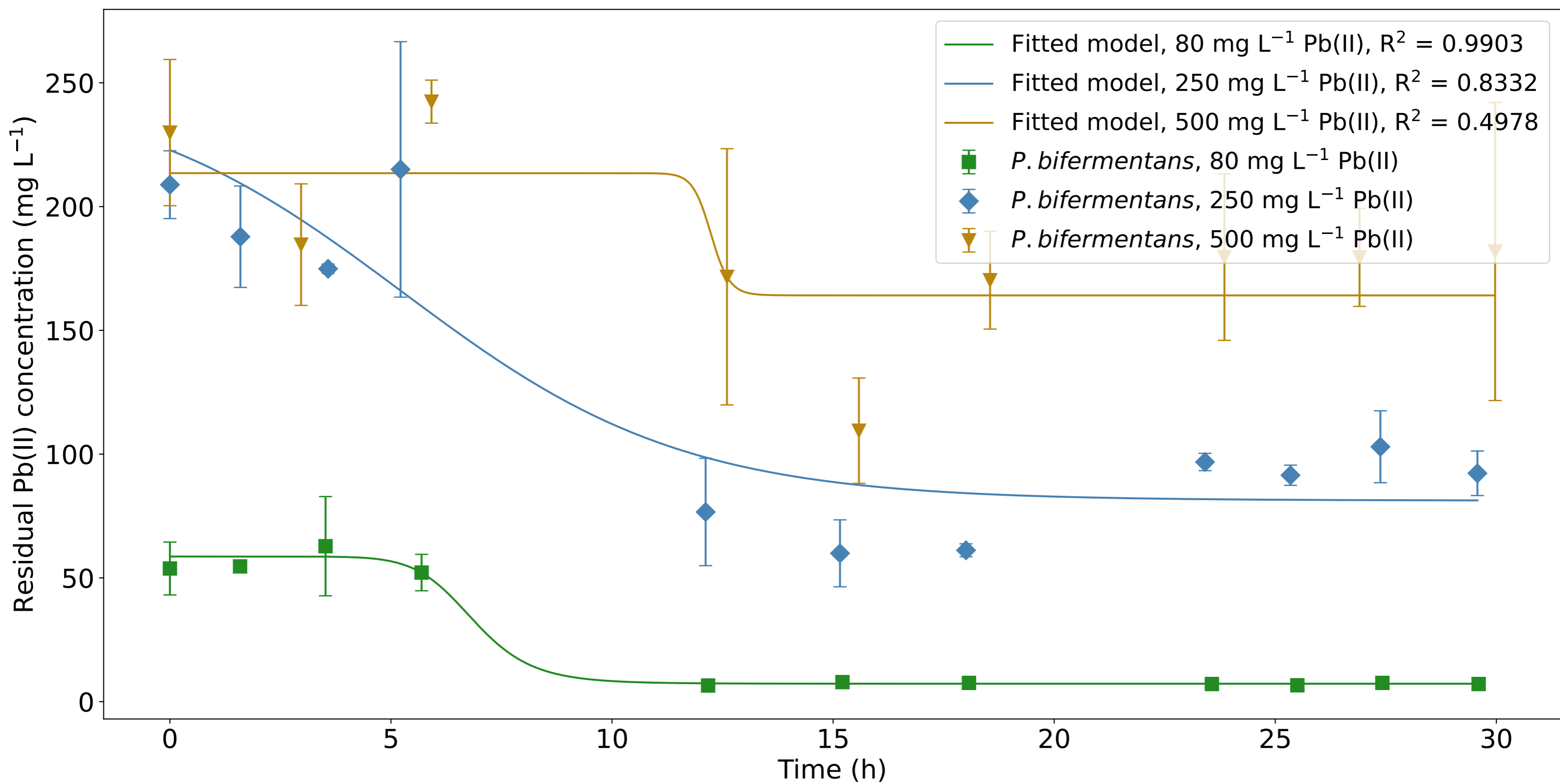

Supplement: Supplementary file 1 [file ijms-23-12255-s001.zip › Definitions/Pb_C_Fitted.pdf]

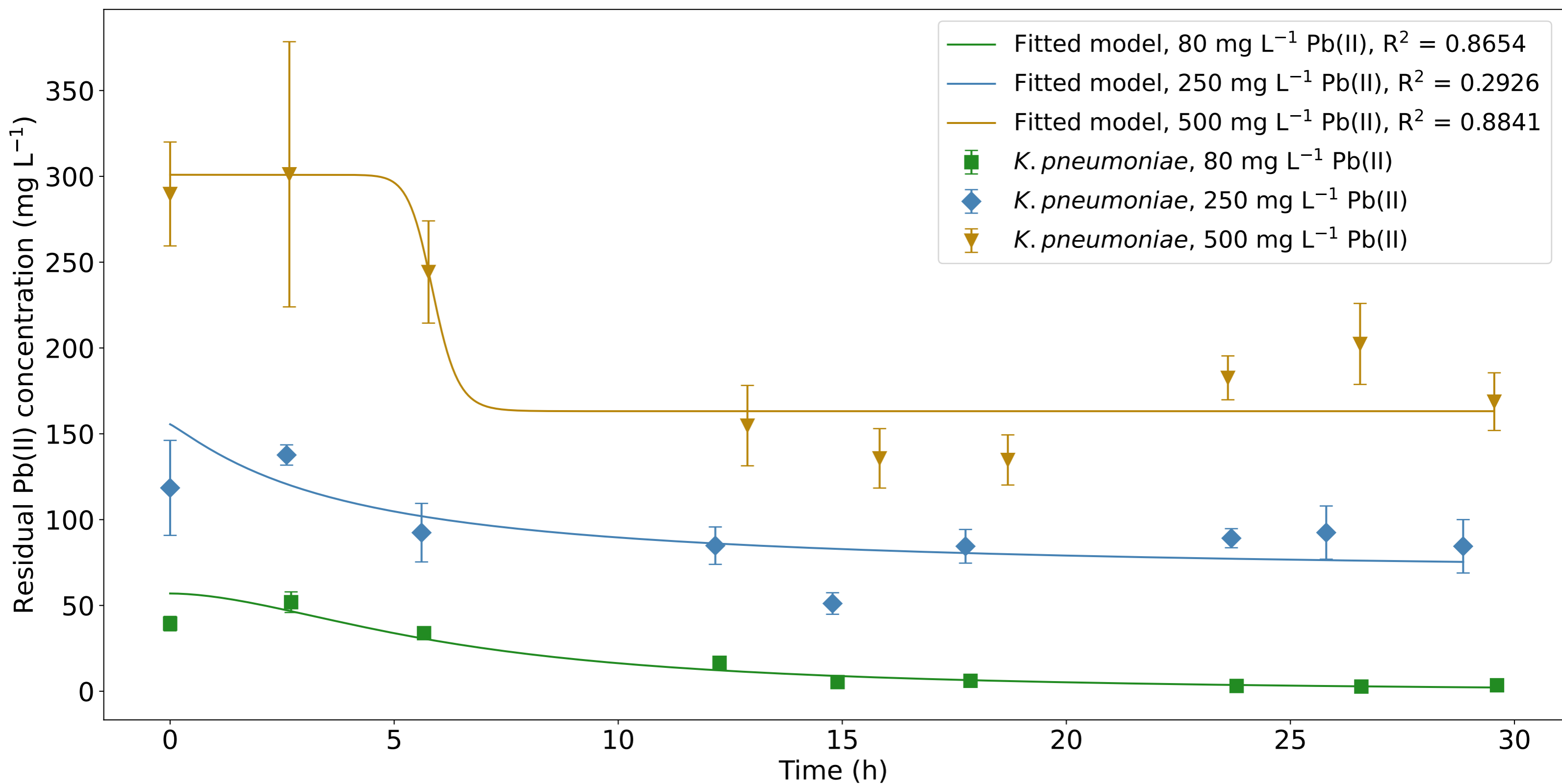

Supplement: Supplementary file 1 [file ijms-23-12255-s001.zip › Definitions/Pb_K_Fitted.pdf]

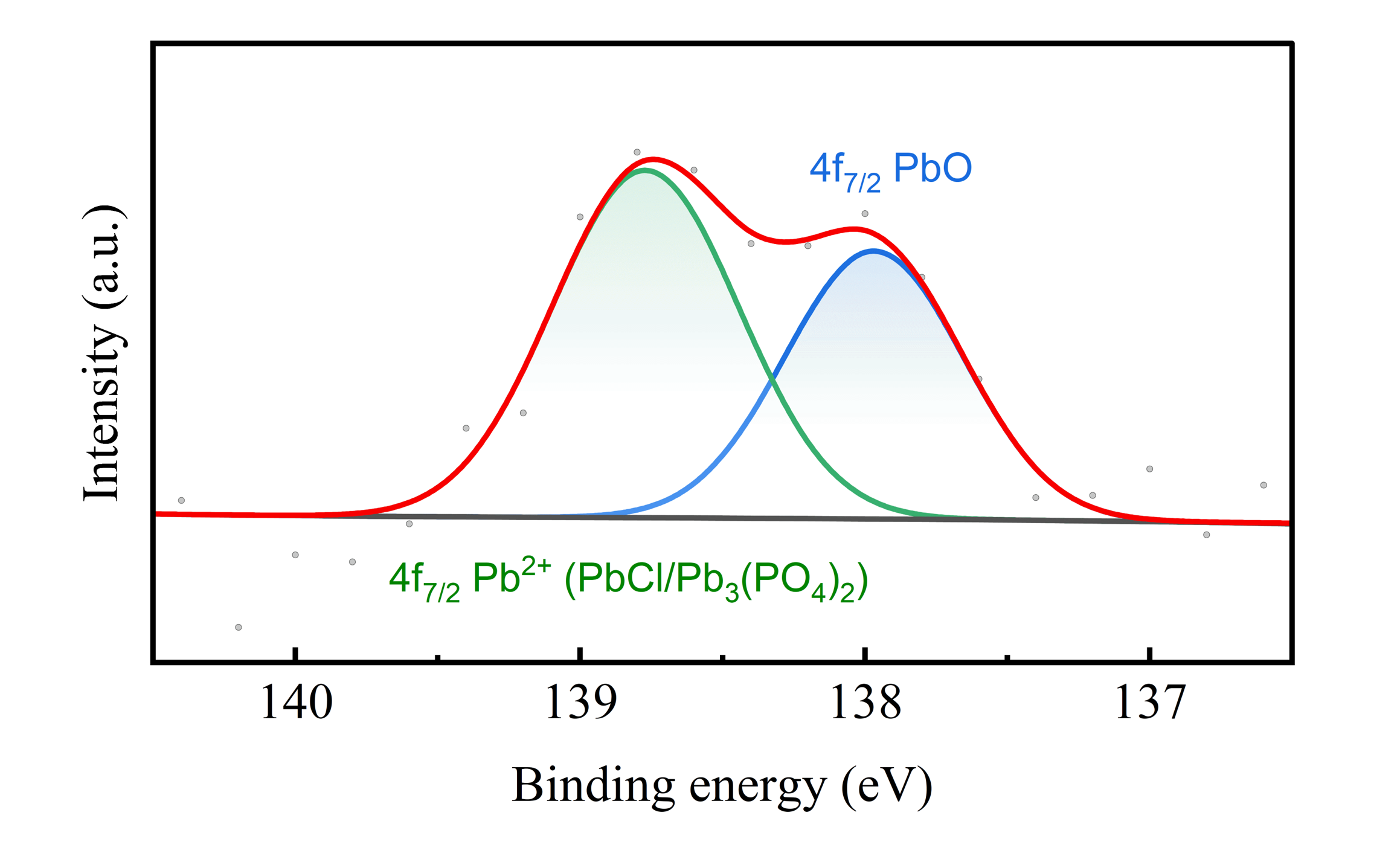

Supplement: Supplementary file 1 [file ijms-23-12255-s001.zip › Definitions/XPS_kleb.png]

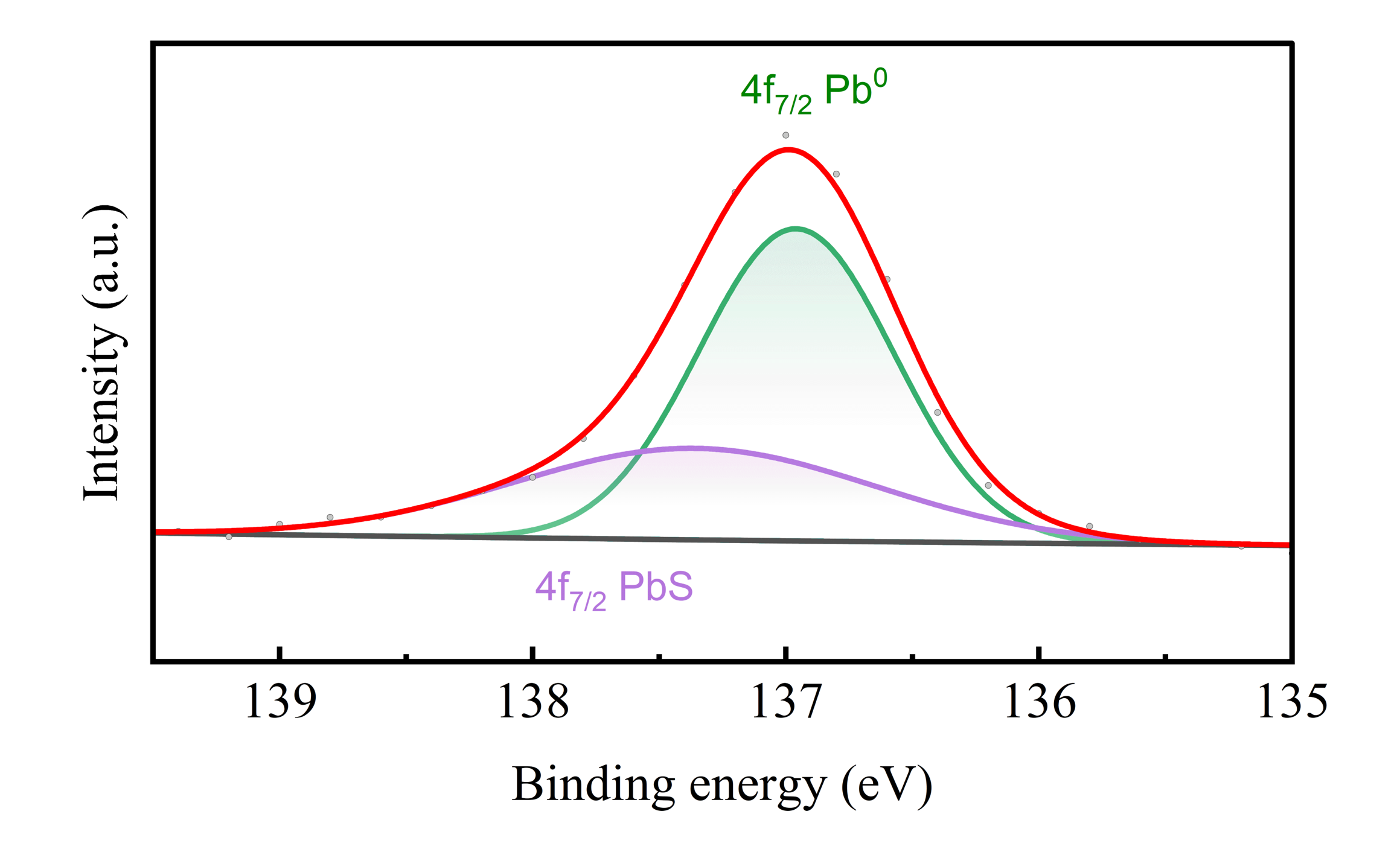

Supplement: Supplementary file 1 [file ijms-23-12255-s001.zip › Definitions/XPS_para.png]
